# A Response Surface Model to Predict and Experimentally Tune the Chemical, Magnetic and Optoelectronic Properties of Oxygen‐Doped Boron Nitride**

**DOI:** 10.1002/cphc.202100854

**Published:** 2022-05-19

**Authors:** Ravi B. Shankar, Elan D. R. Mistry, Daphné Lubert‐Perquel, Irena Nevjestic, Sandrine Heutz, Camille Petit

**Affiliations:** ^1^ Barrer Centre Department of Chemical Engineering Imperial College London South Kensington Campus, Exhibition Road London SW7 2AZ United Kingdom; ^2^ Department of Chemistry Imperial College London South Kensington Campus, Exhibition Road London SW7 2AZ United Kingdom; ^3^ London Centre for Nanotechnology and Department of Materials Imperial College London South Kensington Campus, Prince's Consort Road London SW7 2BP United Kingdom; ^4^ Current address: Department of Chemical Engineering University College London London WC1E 7JE United Kingdom; ^5^ Current address: National High Magnetic Field Laboratory Tallahassee FL 32306 U.S.A.; ^6^ Department of Physics University of Florida Gainesville FL 32611 U.S.A.

**Keywords:** band gap, boron nitride, design of experiments, doping, EPR spectroscopy

## Abstract

Porous boron nitride (BN), a combination of hexagonal, turbostratic and amorphous BN, has emerged as a new platform photocatalyst. Yet, this material lacks photoactivity under visible light. Theoretical studies predict that tuning the oxygen content in oxygen‐doped BN (BNO) could lower the band gap. This is yet to be verified experimentally. We present herein a systematic experimental route to simultaneously tune BNO's chemical, magnetic and optoelectronic properties using a multivariate synthesis parameter space. We report deep visible range band gaps (1.50–2.90 eV) and tuning of the oxygen (2–14 at.%) and specific paramagnetic OB_3_ contents (7–294 a.u. g^−1^). Through designing a response surface *via* a design of experiments (DOE) process, we have identified synthesis parameters influencing BNO's chemical, magnetic and optoelectronic properties. We also present model prediction equations relating these properties to the synthesis parameter space that we have validated experimentally. This methodology can help tailor and optimise BN materials for heterogeneous photocatalysis.

## Graphical abstract

**A response surface model** to synthesize O‐doped boron nitride with varying band gaps, O content and OB_3_ content is reported and experimentally validated.

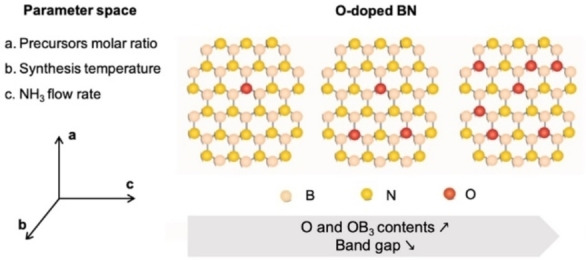

## Introduction

The surface chemistry and optoelectronic properties play an important role in the behaviour and performance of semiconductors. Engineering the band structures in semiconducting materials is of utmost importance across several fields in solid‐state physics. These include, but are not restricted to, heterogeneous photocatalysis, sensing and single photon quantum emission.[^1^, ^2^, ^3^] Chemical modification, *via* atomistic doping, has been utilised to tailor the photochemistry of semiconductors.[^4^, ^5^, ^6^, ^7^, ^8^] Indeed, this has been true for hexagonal boron nitride (BN), the isoelectronic analogue of graphene, which has been recently employed for the applications listed above.[^9^, ^10^, ^11^, ^12^, ^13^, ^14^] A handful of elements have been used to dope both amorphous and crystalline forms of BN, such as carbon,[^5^, ^15^, ^16^, ^17^] oxygen,[^18^, ^19^] boron,^[9]^ sulphur,[^20^, ^21^] and silicon,^[22]^ as well as metals, such as molybdenum,^[23]^ nickel^[24]^ and copper.^[25]^ In this study, we focus our attention on oxygen doping in BN and particularly porous boron BN, a combination of hexagonal (hBN), turbostratic (tBN) and amorphous BN (aBN). Oxygen doping in BN involves the preferential substitution of nitrogen atoms with oxygen atoms within the lattice, leading to boron oxynitride (BNO). Preferential substitution of nitrogen over boron atoms has been reported in the literature, with the latter creating an unstable state due to a high degree of structural deformation.^[26]^ The embedment of oxygen atoms into the BN lattice has proven effective in boosting the specific surface area and porosity, which has led to enhanced adsorption capacities during molecular separations and gas storage.[^27^, ^28^, ^29^, ^30^] Computational and experimental work has shown that oxygen doping can introduce semiconducting and magnetic properties in BN materials.[^18^, ^19^, ^26^, ^31^] This is particularly relevant given the recent interest in employing BN for photocatalysis.[^9^, ^10^, ^11^]

Through a computational DFT study, Weng *et al*.^[18]^ showed that doping, and tuning the content of, oxygen can narrow the optical band gap of BN to induce semiconductor behaviour. DFT simulations predicted a theoretical trend of band gaps in the range of 1.7–4.6 eV, corresponding to oxygen contents between 5–25 at. %. However, these trends remained theoretical predictions, which must be validated experimentally. Furthermore, understanding how to experimentally tune the oxygen content, and how it impacts the band gap, is critical to using BN‐based materials in optoelectronic applications.

Taking this into consideration, the primary outcomes of this study are threefold. Firstly, we designed a systematic route to tune the chemistry, namely oxygen content, and band gap of BNO over a wide range using multiple independent synthesis variables. In doing so, we experimentally tested and verified the DFT predictions of Weng *et al*.,^[18]^ and showed that higher oxygen contents can indeed lead to lower band gaps in BN materials. Secondly, we applied a design of experiments (DOE) approach, using the experimental data, with the aims of: (i) identifying the key synthesis parameters influencing the chemical, magnetic and optoelectronic properties of BNO and (ii) predicting the aforementioned properties for different synthesis conditions using model equations. As part of the DOE approach outlined herein, we conducted in‐depth statistical analyses to identify potential outliers, influential data points and evaluate the goodness of fit of the model predictions to the experimental data (statistical model). These tests are an essential component of the DOE process to avoid an ad‐hoc or skewed modelling approach and ensure accurate predictions of the material properties. Finally, we experimentally validated the DOE equation predictions for the oxygen content and band gap of BNO for contrasting synthesis conditions. The underlying highlight of the DOE strategy employed herein is that the methodology can be extended to other forms of doped BN synthesised *via* a bottom‐up approach. Such approach has for instance been used to tune the optoelectronic properties of BN using elements such as C and S.[^5^, ^32^] Hence, we envisage that such a model would be a valuable tool for the community in facilitating systematic tailoring of the chemical, magnetic and optoelectronic properties of BN materials towards given reactions.

## Methodology

### Synthesis of BNO

In a typical synthesis, a reaction mixture (total of 60 mmol) of boric acid (H_3_BO_3_, ACS reagent, 99.0 %, Sigma‐Aldrich) and hexamethylenetetramine (HMTA) (C_6_H_12_O_6_, molecular biology grade, Sigma‐Aldrich) in varying molar ratios (boric acid:HMTA=1 : 2, 2 : 1 and 5 : 1) were added to 100 mL of deionised water at 90 °C under rapid stirring to form a boric acid‐HMTA complex in solution. The solution was allowed to evaporate overnight until the resulting white powder was collected and subsequently dried for 24 hours at 90 °C in a drying oven. The dried material was transferred to an alumina boat crucible (approx. 1.4 g), which was placed in a horizontal tubular furnace. The sample was initially maintained at ambient temperature for 30 minutes under pure ammonia flow, with the flowrate set to 250 mL min^−1^ to establish an ammonia rich atmosphere. Once this step was complete, the ammonia flow rate was set to a chosen flowrate (either 50 mL min^−1^, 150 mL min^−1^ or 250 mL min^−1^) and the sample was heated from ambient temperature to a set temperature (either 800 °C, 1000 °C or 1200 °C) with a ramp rate of 10 °C min^−1^. This steady‐state temperature was maintained for 3 hours, after which the samples were allowed to naturally cool to approximately 600 °C under the same ammonia flow rate. At this point, the ammonia flow was shut off and inert argon gas was flowed through at a rate of 100 mL min^−1^ overnight until the furnace had cooled to room temperature. Upon completion of the synthesis, either light brown or yellow powders were obtained, which we refer to as BNO. In total, 27 samples were produced.

### Materials Characterisation

*Powder X‐ray Diffraction (XRD)* was performed using a PANalytical X'Pert Pro X‐ray diffractometer in reflection‐transmission mode with a spinning stage (2 revolutions/second). An anode voltage of 40 kV and emission current of 20 mA were chosen as the operating conditions using a monochromatic Cu−Kα radiation source (λ=1.54178 Å). The X'Celerator silicon strip detector was used in the diffractometer.

*Fourier Transform‐Infrared Spectroscopy (FT‐IR)*. The samples were first ground to a powder using an agate mortar. Subsequently, the spectra were obtained in the range of 500–4000 cm^−1^ using a Perkin‐Elmer Spectrum 100 FT‐IR spectrometer equipped with an attenuated total reflectance (ATR) accessory. The spectra were collected, averaged 16 times, and corrected for the background noise.

*X‐ray Photoelectron Spectroscopy (XPS)* was employed to determine the elemental composition of the samples and the chemical states of the elements, using a Thermo Scientific K‐Alpha^+^ X‐ray Photoelectron Spectrometer equipped with a MXR3 Al Kα monochromated X‐ray source (hν =1486.6 eV). The samples were initially ground and mounted onto an XPS sample holder using a small rectangular piece of conductive carbon tape. The X‐ray gun power was set to 72 W (6 mA and 12 kV). Survey scans were acquired using 200 eV pass energy, 0.5 eV step size and 100 ms (50 ms×2 scans) dwell times. All of the high resolution core level spectra (B 1*s*, N 1s, C 1s, and O 1s) were obtained using a 20 eV pass energy and 0.1 eV step size. The results were analysed using the Thermo Avantage data analysis program. Any charging effect in the core level measurements was mitigated by using a dual‐beam flood gun that uses the combination of low energy electrons and argon ions. To determine the standard deviation in the measured oxygen content and atomic composition, three repeat measurements were conducted for two BNO samples.

*Electron Paramagnetic Resonance (EPR) spectroscopy* experiments were acquired using a Bruker Elexsys E580 CW EPR spectrometer operating at X‐band frequencies (9–10 GHz/0.3 T), equipped with a Bruker ER4118‐X MD5 resonator. All spectra were recorded at room temperature in air atmosphere in 4 mm EPR suprasil tubes. Spectra were acquired using 0.2 mW of microwave power with field modulation of 100 kHz frequency and 2G amplitude in the detection sequence.

*UV‐Vis Diffuse Reflectance (UV‐Vis DR) Spectroscopy* was conducted using a Shimadzu UV‐2600 true optical double beam UV‐Vis spectrophotometer equipped with an integrating sphere. The integrating sphere has an InGaAs detector with a detection range of 220–1400 nm. Spectral band width was set to 5 nm and barium sulphate (BaSO_4_) was used as a standard for the baseline corrections. Spectra were treated using Kubelka‐Munk function in order to eliminate any tailing contribution from the UV‐Vis DR spectra. Equation (1) was applied where R is the reflectance (%):

(1) $$F\left(R\right)=\frac{{\left(1-R\right)}^{2}}{2R}$$

The band gaps (E_G_) were estimated *via* extrapolation of the linear section of the Tauc plot of *[*F(R).hv]^*1/n*^ against photon energy (*hν*). We consider BNO as a direct band gap semiconductor based on literature (*n*=0.5, exponent of the Tauc plot that relates to the nature of the transition).^[33]^ The absorption onset was determined by a single tangent method and the crossing with the X‐axis is taken as the value. To determine the standard deviation in the measured band gaps, three repeat measurements were conducted for three different BNO samples.

#### DOE and Response Surface Design Process

Using the experimental data collected for all 27 BNO samples, we employed a combined DOE and response surface approach using the JMP software.^[34]^ JMP is a collection of software used for statistical analysis developed by SAS. It is a data analytics tool utilising complicated statistics to help understand complex relationships between data samples. The aims of our approach were to: (i) ascertain the key synthesis parameters influencing the chemical, magnetic and optoelectronic properties of BNO and (ii) develop model equations to predict the aforementioned properties for different synthesis conditions. The former was achieved by fitting a response surface design, which mapped each output variable to the synthesis parameter space. The constructed model effects utilised included second order polynomials, cross‐product, and one‐way interactions with response surface considerations. Role variables included the oxygen content, the specific paramagnetic OB_3_ intensity and the band gap. The response surface was then subsequently used to obtain the model predictions equations expressing the oxygen content, specific content of paramagnetic OB_3_ sites and the band gap as a function of the synthesis parameter space. The model is purely statistical and therefore does not carry any physical or chemical assumptions regarding the synthesis mechanism of porous boron nitride.

**Figure 1:**
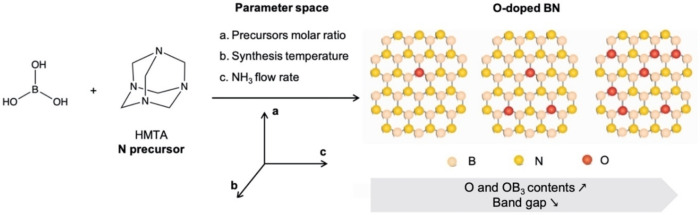
Synthesis route and parameter space used to tune the chemistry, magnetic and optoelectronic properties of oxygen‐doped BN (BNO).

A flowchart illustrating the DOE and response surface approach applied herein is presented in Figure S1 to guide the reader stepwise through the process. The complete results for all of the statistical tests and analyses conducted in the DOE process is presented in the Supporting Information. These statistical tests are inherent to the DOE/response surface design process and were conducted thoroughly to ensure a rigorous modelling approach was adopted herein. The first stage of the process entails the collection and analysis of the experimental trends in the material properties of interest (in our case the oxygen content, specific paramagnetic OB_3_ content, and the band gap) against the synthesis parameter space. The three desired output variables are each mapped against three independent variables (synthesis temperature, ammonia flowrate and starting ratio of precursors – see Figure 1), and each independent variable spans a wide range of values. As such, this necessitates multivariate analysis, which involves the observation and analysis of more than one statistical outcome variable at a time.^[35]^ The selection of the synthesis parameters is based on our experience from previous studies focused on boron nitride.[^10^, ^36^, ^37^]

#### Goodness of Fit Metrics for Experimental Data and Model Predicted Values

The goodness of fit and error between the model and experimental values for oxygen content and band gap was quantified through the coefficient of determination (R^2^) and root mean square deviation (RMSD), respectively. The RMSD is defined as the square root of the mean square error (MSE). The RMSD and MSE are measures of accuracy between model and observed values. The closer the MSE and RMSD to zero, the smaller the residuals and stronger the fit to experimental data.^[38]^

## Results and Discussion

### Experimental Characterisation

We synthesised 27 oxygen‐doped BN (BNO) samples using an adapted version of the synthesis outlined by Weng *et al*.,^[18]^ which relies on a bottom‐up pyrolytic synthesis at elevated temperatures using a mixture of boric acid and hexamethylenetetramine (HMTA) in the presence of ammonia (Supporting Information).^[18]^ The parameter space that we considered included: (i) the synthesis temperature, (ii) the ammonia flowrate, and (iii) the starting molar ratio of the boric acid and HMTA precursors. These parameters were specifically chosen for the following reasons. The synthesis temperature influences the decomposition endpoint of the precursors and the extent of reaction to form B−N bonds. Ammonia is commonly used as an additional nitrogen source and strong reducing agent to induce the formation of B−N bonds and eliminate carbon.[^39^, ^40^] The residence time of ammonia in the reactor, which governs how much it interacts with the precursors, is influenced by its flowrate. Hence, the flowrate could influence the proportion of B, N, C and O atoms in the final product. Boric acid and HMTA serve as the boron/oxygen and nitrogen precursor, respectively. An excess of boric acid implies a larger starting concentration of oxygen in the reaction mixture, which could result in a larger oxygen content in the resulting product.

**Figure 2:**
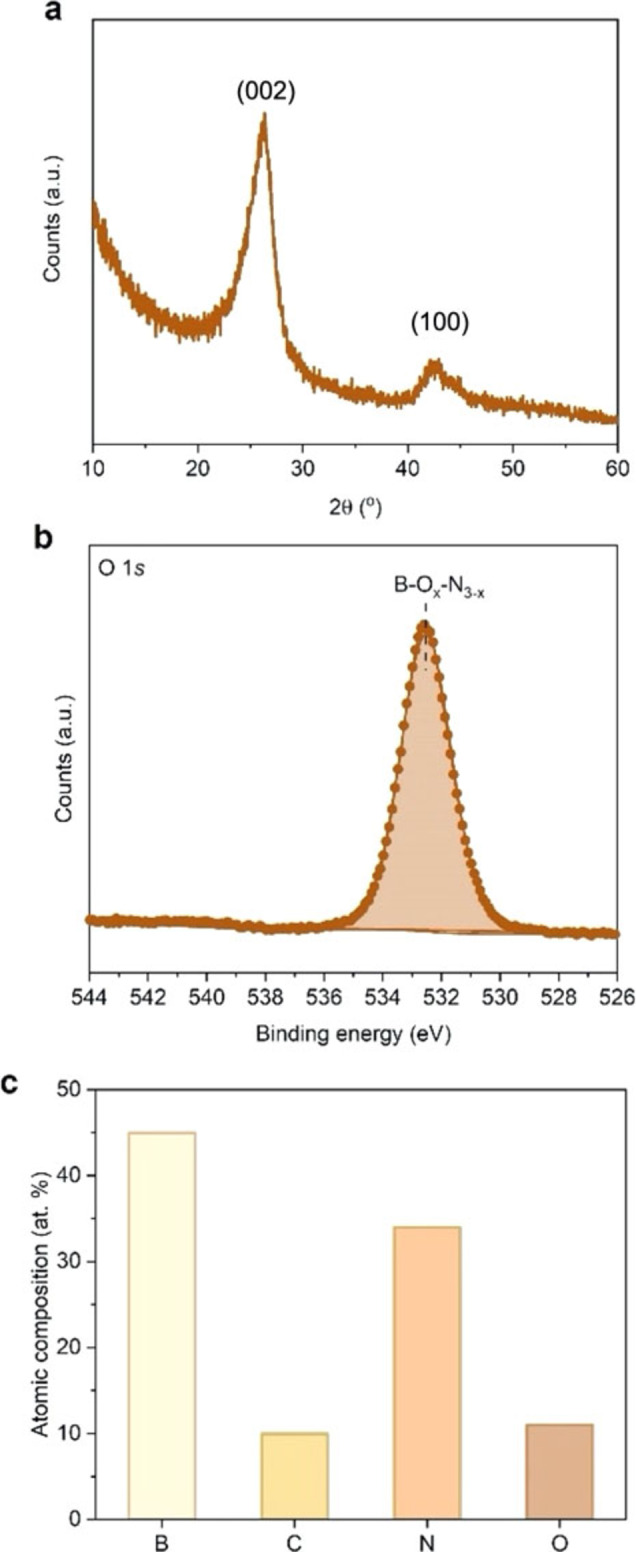
Experimental characterisation of a representative BNO sample synthesised at 800 °C, 2 : 1 molar ratio and 50 mL min^−1^. (a) Powder XRD pattern of the representative BNO sample, (b) High resolution O 1s core level spectrum for the representative BNO sample with the boron oxynitride (B−O_x_−N_3–x_) species highlighted, (c) Atomic composition of the representative BNO sample as obtained from XPS analysis.

**Figure 3:**
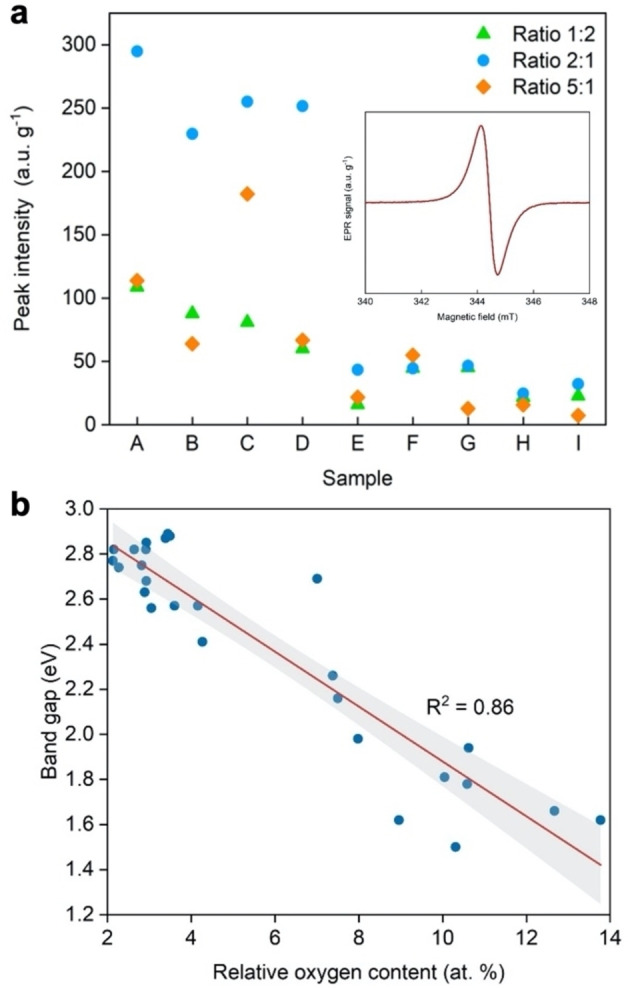
(a) Peak intensities derived from room temperature X‐band EPR spectra for all BNO samples investigated in this study (see Table S3 for sample names). Inset: Paramagnetic signature of the representative BNO sample obtained using X‐band EPR spectroscopy at 298 K with radical peak originating from isolated OB_3_ sites; (b) Scatter plot of the band gap against the oxygen content for all BNO samples investigated in this study. Least squares linear regression was applied to obtain the line of best fit (red) and the 95 % confidence intervals (grey shaded area). The coefficient of determination (R^2^) illustrating the goodness of the linear fit is shown in each plot.

We present the experimental characterisation of the structural, chemical, magnetic and optoelectronic properties of a representative BNO sample in Figures 2 and 3. A detailed characterisation for all 27 BNO samples synthesised in this study is presented in the Supporting Information (Figures S2–S30). To gain insight into the morphology and structure of BNO, we first collected powder XRD patterns (Figure 2a and Figure S2). The XRD patterns confirm the turbostratic nature of the materials with only broad peaks at 2θ values of 26° and 44°, corresponding to the (002) and (100) planes, respectively.^[41]^ We note that prior studies from our group provide corroborating details on the structural features of the material.[^10^, ^36^, ^37^] We characterised the chemical structure and composition in BNO using FT‐IR spectroscopy and XPS (Figure 2 and Supplementary Figures 3–30). All the samples exhibit the two characteristic IR bands of BN at ∼1380 cm^−1^ (in‐plane B−N transverse stretching) and ∼800 cm^−1^ (out‐of‐plane B−N−B bending).^[42]^ We also observed a distinct B−O band at ∼1000 cm^−1^, attributed to in‐plane substituted oxygen atoms (Figure S2).[^10^, ^18^] Some of the samples exhibit a weak band at ∼3400 cm^−1^, which suggests the presence of edge‐conjugated hydroxyl groups, arising from nanosheet edge functionalisation.^[43]^ A few samples, mostly those obtained at low or moderate temperature, show a weak band at ∼2900 cm^−1^, corresponding to the C−H bond, suggesting the presence of residual carbon from the HMTA precursor (Figure S3). To gain further insight into the atomic surface composition and chemical states of the elements, we collected high resolution core level spectra through X‐ray photoelectron spectroscopy (XPS). The high resolution O 1s core level spectrum and atomic composition for the representative BNO sample is shown in Figures 2b and 2c, respectively. The peak centred at 533.1 eV in Figure 2b is attributed to boron oxynitride (B−O_*x*_−N_*3‐x*_) species, which stems from the in‐plane substitution of oxygen atoms in the BN lattice, as we have described in a prior studies.[^9^, ^10^] The analysis indicates a significant proportion of oxygen (10.6±0.6 at. %) in the BNO sample (Figure 2c). A few samples, mostly those obtained at low temperature and where boric acid (i. e. oxygen precursor) was not in excess, exhibit comparatively similar carbon contents, which we link to residual carbon atoms from the HMTA precursor and the possible presence of adventitious carbon impurities on the surface (Figure 2c). However, when higher temperatures (>1000 °C) and a significant excess of boric acid:HMTA (5 : 1) were used, the residual carbon content is comparatively much lower than the oxygen content.

The survey spectra and complete deconvoluted B 1s, N 1s and O 1s core level spectra for all of the samples synthesised in this study are presented in Figures S3–S30. The atomic compositions of all samples are presented in Table S1. The standard errors of the XPS measurement obtained through repeat measurements for two BNO samples within the sample set are presented in Table S2. The formation of BN is confirmed through the fitted core level spectra of B 1s and N 1s (Figures S3–S30) which show the presence of B−N bonds (191.0 eV for B 1s and 398.5 eV for N 1s).[^44^, ^45^] We also observe shake‐up satellite peaks in the B 1s and N 1s core level spectra for BNO (Figures S3–S30), which evidences the presence of an sp^2^‐hybridised BN phase.^[46]^ We were able to tune the oxygen content over a wide range of 2–14 at. %. It should be noted that excessive oxygen doping (>20 at. %) can lead to buckling and mechanical instability in the BNO sheet.^[18]^ Literature studies have shown that boron substitution with oxygen atoms leads to both thermodynamic and mechanical instability,[^18^, ^26^, ^31^] hence the doped oxygen atoms preferentially substitute nitrogen atoms.

The presence of the boron oxynitride (B−O_*x*_−N_*3‐x*_) species observed in Figure 2b can give rise to the formation of isolated OB_3_ sites. In a previous study,^[9]^ we showed that these isolated OB_3_ sites induce a paramagnetic radical signal through the introduction of an unpaired electron from the incoming oxygen atom to the system, which contributes to band gap narrowing. The paramagnetic signature of the BNO species, measured using X‐band electron paramagnetic resonance (EPR) spectroscopy under ambient conditions, is shown in Figure 3a (inset). The intensity of the EPR signal is proportional to the radical content and the quantity of the isolated OB_3_ chemical states in the BNO material. The complete EPR spectra for all 27 BNO samples are presented in Figure S31 while the OB_3_ peak intensities are displayed in Figure 3a and summarised in Table S3. Akin to the oxygen content, we were able to tune the magnitude of the paramagnetic signal over a wide range (7–294 a.u. g^−1^), which indicates contrasting proportions of isolated OB_3_ sites across the 27 BNO samples. More precisely, Figure 3a shows that to maximise OB_3_ content, the lower temperature and flow rate should be used, regardless of precursor ratio. However, using a 2 : 1 starting ratio provides nearly three‐fold increase in OB_3_ content compared with the other mixtures studied.

Next, we probed the optoelectronic properties of BNO through UV‐Vis diffuse reflectance spectroscopy (UV‐Vis DRS). The absorption spectra and tabulated band gaps for all BNO samples are presented in Figures S32–S34 and Table S3. The standard errors in the UV‐Vis measurements obtained through repeat measurements for three different BNO samples within the sample set are presented in Table S4. The lowest band gap observed amongst the sample set was 1.50 eV (corresponding to synthesis conditions of 800 °C, 2 : 1 molar ratio, 250 mL NH_3_ min^−1^). To date, this is the lowest band gap achieved experimentally in BN materials without the use of external atomistic dopants (*i. e*., elements outside of the constituent B, N and O atoms).[^5^, ^47^]

We tested experimentally the theoretical predictions of Weng *et al*.^[18]^ through a scatter plot of the band gaps in BNO related to their oxygen content in Figure 3b. The experimental data was regressed with a least squares line of best fit with a 95 % confidence interval. The DFT simulations of Weng *et al*. predicted that band gaps as low as 1.70 eV could theoretically be achieved through tuning the oxygen content in BN. This is verified experimentally in Figure 3b, with experimental band gaps as low as 1.50 eV achieved. A good correlation between the band gap and oxygen content is observed, with a coefficient of determination (R^2^) of 0.86. However, we notice that some samples with the same oxygen content exhibit different band gaps. This suggest that besides the O content, other factors or a combination of factors may influence the band gap. Among them, one can cite the varying proportion of different chemical states of oxygen (e. g. OB_3_ sites) and carbon.

### Stage 1: Experimental Trends across Multi‐Dimensional Sample Set

**Figure 4:**
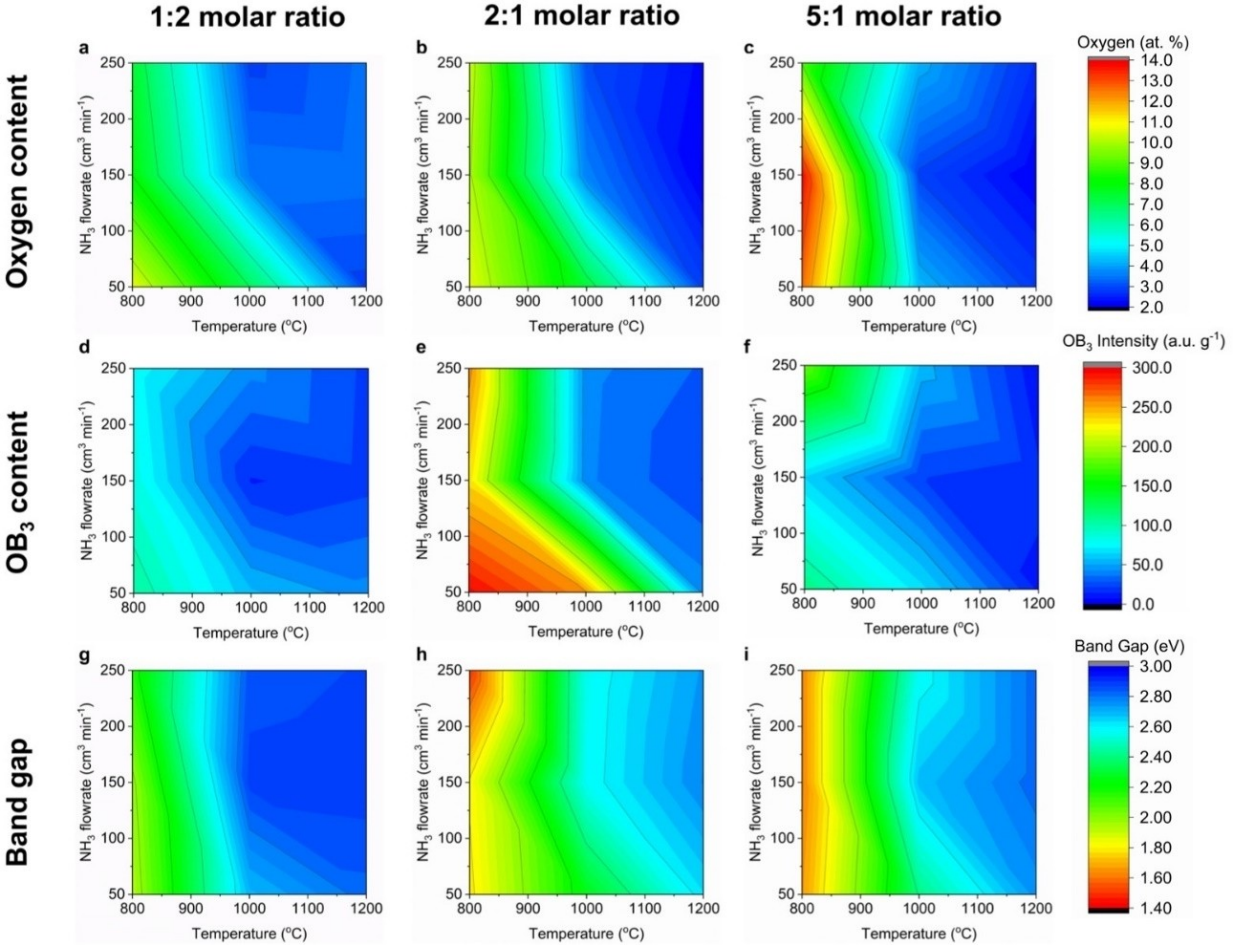
Heat maps depicting the variation in the oxygen content, specific paramagnetic OB_3_ content and band gap across the multivariable synthesis parameter space. The red and blue regions correspond to higher and lower values of each material property, as indicated by the colour scale. A uniform colour scale was used for each output variable (oxygen content, specific paramagnetic OB_3_ content and band gap).

Having experimentally tested and verified the theoretical prediction of Weng *et al*.^[18]^ relating to band gap tuning in BN via oxygen doping, we shift our attention to the second objectives of our study, *i. e*., identifying the key synthesis parameters influencing the chemical, paramagnetic and optoelectronic properties of BNO and predicting the aforementioned properties for different synthesis conditions using model equations. We present the experimental trends for the oxygen content, specific paramagnetic OB_3_ content and band gaps across all 27 BNO samples in Figure 4. Representing the data in this manner facilitates mapping, and identifying links between, the chemical, paramagnetic and optoelectronic properties of BNO across the whole multiple variable synthesis parameter space. Further, it allows one to readily identify the synthesis conditions required for a given response qualitatively. The heat maps show “high” (marked in red) and “low” (marked in blue) regions corresponding to high and low oxygen content and specific paramagnetic OB_3_ content, respectively. For the band gap, the high and low band gap regions are in blue and red, respectively. Across Figures 4a–4c, we observe that the regions of high oxygen content are generally located at lower synthesis temperatures (800–1000 °C) and lower flowrates (50–150 mL min^−1^), which lead to greater retention of oxygen atoms from the starting boric acid precursor. The carbon from HMTA is mostly to completely eliminated from the end product through the reaction with ammonia to form methane for temperatures greater than 800 °C.[^39^, ^40^]

Higher synthesis temperatures, for a given NH_3_ flowrate and starting ratio of precursors, lead to lower oxygen contents (Figures 4a–4c). This is shown by the pronounced drop in the oxygen content and horizontal transition to the blue region at approximately 1000 °C, which is attributed to increased conversion of the precursors to BN and elimination of oxygen from the starting precursor.^[48]^ Similarly, higher NH_3_ flowrates, for a given synthesis temperature and starting ratio of precursors, leads to lower oxygen contents. This is illustrated by the vertical transitions from the green to blue regions for temperatures greater than 950 °C. As predicted, higher flowrates of ammonia, an additional nitrogen source, assist in nitriding the boron/oxygen and nitrogen precursors by breaking the B−O covalent bond in boric acid. This reaction forms B−N bonds and leads to a lower oxygen content.^[49]^ The highest oxygen content (13.8 at. % O) is located in the heat map associated to the BNO samples synthesised with a 5 : 1 molar ratio of boric acid:HMTA (Figure 4c). This is to be expected given a larger starting concentration of oxygen atoms in the reaction. Comparing Figures 4a–4c and 4d–4 f, the regions of high oxygen content somewhat align with the regions of high paramagnetic OB_3_ intensity. This is to be expected as a higher proportion of oxygen in the BNO system increases the likelihood of forming isolated OB_3_ states, leading to a higher intensity observed through EPR.

### Stage 2: Response Surface Design to Identify Significant Synthesis Parameters and Obtain Model Equations

As we have now evaluated the experimental data, we focus our attention to designing the response surface. A response surface maps the output variables to the independent variables in the synthesis parameter space based on the experimental data collected in Stage 1. This stage of the process is essential to satisfy the objectives of our study: (i) identifying the key synthesis parameters influencing the chemical, magnetic and optoelectronic properties of BNO and (ii) developing a model prediction tool to forecast the latter for different synthesis conditions. A response surface was fitted, as opposed to a full factorial design, to account for curvature within the experimental data set.[^50^, ^51^] To do so, we utilised synthesis parameter effects up to a second order polynomial (*i. e*., *T* ^*2*^, *F* ^*2*^ and *R* ^*2*^, where *T*, *F* and *R* denote synthesis temperature, flowrate and precursors ratio, respectively) and their combinatorics (*i. e*., *T⋅F*, *T⋅R* and *F⋅R)*. To identify the synthesis parameter(s) having the largest influence on the oxygen content, the magnitude of paramagnetic OB_3_ sites and the band gap in BNO, we used the response surface to construct effects summaries at the 5 % significance level (α=0.05) (Figure S36). An effects summary provides insight into how influential an independent variable is on the outcome of a measured variable.^[52]^ This is determined through a hypothesis test, which examines the statistical significance/influence of each synthesis parameter on the output variables. The details of how we performed the hypothesis test are provided in the Supporting Information.

The effects summaries for the oxygen content, specific paramagnetic OB_3_ content and band gap, before and after removal of statistically insignificant parameters, are presented in Figure S36 along with the tabulated *p*‐values for the oxygen content, specific paramagnetic OB_3_ content and band gap in Tables S5–S7. The synthesis temperature (*T*) was found to have the most statistically significant influence across the oxygen content, specific paramagnetic OB_3_ content and band gap.

Using the response surface fitted to the experimental data, we obtained model prediction equations relating the oxygen content, specific paramagnetic OB_3_ content and band gap to the synthesis parameter space. The model prediction equations for each material property take the general form as shown below.

(2) $$Oxygen/{OB}_{3}/Band\;gap=A+B\left(F\right)+C\left(R\right)+D\left(T\right)+E\left(R^{2}\right)+F\left(T^{2}\right)+G\left(R\cdot T\right)\;\mathrm{Equation}$$

Here, A−G denote coefficients that take different values depending on whether one considers the oxygen content, specific paramagnetic OB_3_ content or band gap. The coefficients are tabulated in Table 1 below.

**Table 1:** Summary of the coefficients (to 3 significant figures) for the model prediction equation (Equation (2)) of the oxygen content, specific paramagnetic OB_3_ content and band gap.

Material property	A	B	C	D	E	F	G
O content	73.9	−8.87×10^−3^	2.28	−0.123	3.19×10^−2^	−5.48×10^−5^	−1.98×10^−3^
Paramagnetic OB_3_ content	929	−0.109	106	−1.53	−17.7	6.09×10^−4^	7.65×10^−3^
Band gap	−7.23	4.17×10^−4^	−0.465	1.80×10^−2^	4.32×10^−2^	−8.00×10^−6^	1.75×10^−4^

**Figure 5:**
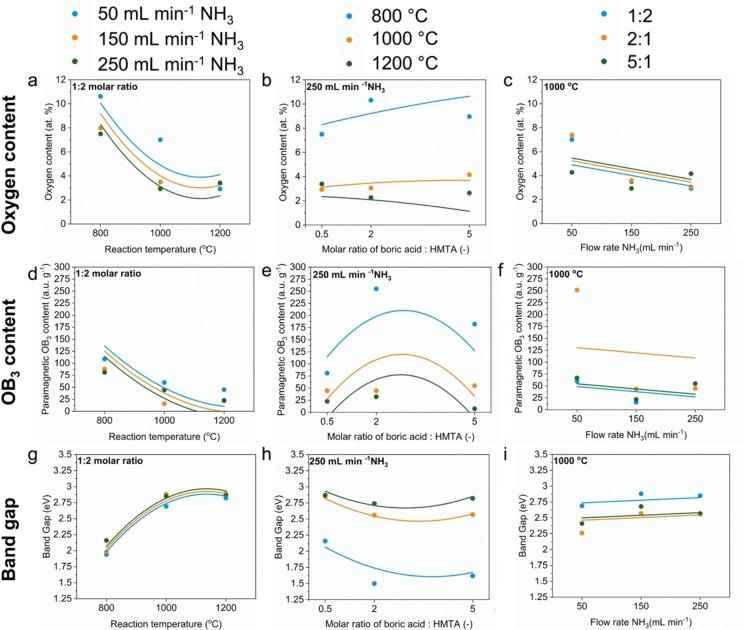
Prediction profiler comparing the model predicted values for the oxygen content, specific paramagnetic OB_3_ content and band gap, as a function of the synthesis parameters, with the experimental data. The model predicted values (lines) and experimental data (symbols) for each synthesis parameter value are superimposed with the same y‐axis increment scale to allow direct comparisons.

Using these equations, one could predict the chemical, paramagnetic and optoelectronic properties of BNO prior to synthesising a BNO material. This tool would facilitate precise tailoring of BNO materials for a given application and avoids unnecessary ad‐hoc trials to identify the required synthesis conditions. To qualitatively assess the goodness of fit of the model equations to the experimental data, we present a prediction profiler in Figure 5. The profiler superimposes the model‐predicted trends for the oxygen content, specific paramagnetic OB_3_ content and band gap (curved lines) over the discrete experimental values (bar graphs). The profiles are presented for a single combination of synthesis parameters; similar profiles of all other combinations can be obtained using the model prediction equations and the experimental data presented in Table S3. Qualitatively, the model equations show good agreement with the experimental values, particularly for the band gap. More discrepancies are observed for some data points of the oxygen content and OB_3_ content.

### Stage 3: Multivariate Outlier Analysis

In Stage 2, we designed a response surface, through which we identified the most statistically significant synthesis parameters on the output variables and obtained the model predictions equations for the oxygen content, specific paramagnetic OB_3_ content and band gap. We now move to Stage 3 of the DOE process (Figure S1), involving the identification of outliers. An outlier is defined as a data point, whose response (value in *y*) lies near three standard deviations from the mean of the responses (*y* values) of the other data points.[^53^, ^54^] This is an important step to assess whether experimental data points, either individual or a collection, do not disproportionately influence the response surface design or the model predictions. For this purpose, we apply the studentised residuals test. The details of how we performed this test are provided in the Supporting Information. The results for the studentised residuals tests for the oxygen content, specific paramagnetic OB_3_ content and band gap are presented in Figure S37. Across the entire sample set, no points are above or beyond the UCL or LCL, respectively (red dashed lines in Figure S37). Hence, no points were classified as outliers or deemed to have a significant influence on the latter stages of the DOE process.

### Stage 4: Identifying Influential Data Points

Prior to making and testing model predictions, it is important to conduct further quantitative statistical tests to: (i) identify influential data points that might induce discrepancy between the model predicted and experimental values and (ii) assess the goodness of fit of the model predictions with the experimental values. Influential data points are those that, if removed from the experimental data set, would significantly skew the predicted responses. This is not to be confused with outliers, which we have previously defined. An outlier has the *potential* to be influential but may not necessarily be so. To identify influential data points, we apply the Cook's distances (Cook's D) test. This test identifies whether individual data points with large residuals do not alter the accuracy of the model predictions. The details of how we performed this test are provided in the Supporting Information. The histograms illustrating the distribution of the Cook's D values for the oxygen content, specific paramagnetic OB_3_ content and band gap are shown in Figure S38. The largest Cook's D values for the oxygen content, specific paramagnetic OB_3_ content and band gap are 0.24, 0.29 and 0.34, respectively. This shows that there are no influential data points that would significantly affect the model predictions.

### Stage 5: Assessing the Goodness of Fit of Model Predictions to Experimental Data

We next assess the goodness of fit of the model predictions (Stage 5, Figure S1) by analysing the residuals for each output variable (oxygen content, specific paramagnetic OB_3_ content and band gap). The residuals are the difference between the model and the experimental values for each output variable. The model responses versus the experimental values for oxygen content, specific paramagnetic OB_3_ content and band gap are presented in Figure S39. Least squares regression was applied to obtain the line of best fit in each case. The data is presented as leverage plots, which are scatter plots that consider the influence of individual data points on the line of best fit. The model responses fit the experimental data well for the oxygen content and the band gap, but not the specific paramagnetic OB_3_ content (oxygen: R^2^=0.90, RMSD=1.28, specific paramagnetic OB_3_ content: R^2^=0.68, RMSD=54.88 and band gap: R^2^=0.96, RMSD=0.10).

When developing the model prediction equations obtained from the response surface, the underlying aim is to ensure that the data points lie as close to the regressed line of best fit as possible. This reduces the error between the predicted model response and the experimental values. In this regard, it is desirable that the residuals follow a normal distribution as this minimises the distance of the predicted model response to the line of best fit.^[55]^ To examine whether the residuals from our model equations follow a normal distribution, we first consider the normal quantile‐quantile (Q−Q) plots for the output variables (Figure S40), and then conduct a Shapiro‐Wilk test. The details of how we obtained the Q−Q plots and conducted the Shapiro‐Wild test are provided in the Supporting Information. Therefore, this shows that the model equations obtained from the response surface have been optimised to minimise the error between the predicted responses and experimental values. The normal distribution of the residuals is presented in the histograms in Figure S41.

### Stage 6: Model Predictions and Validation with Experiments

**Figure 6:**
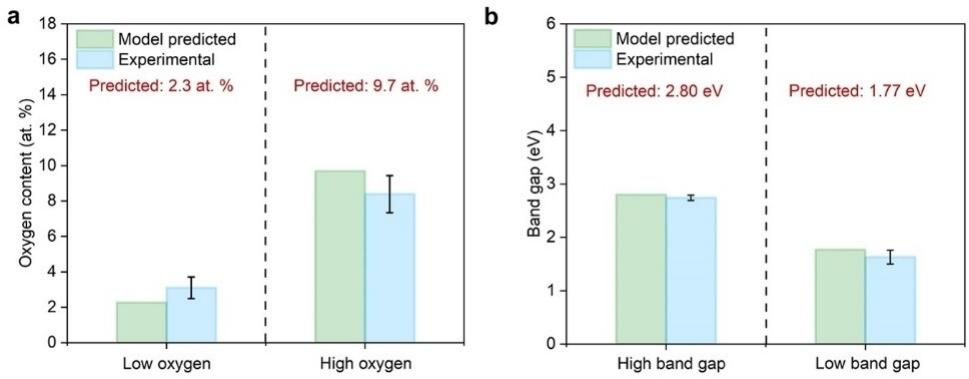
Comparison of model predictions for the high and low (a) oxygen content and (b) band gap BNO samples with the experimental values for validation. The synthesis conditions for the sample with low oxygen content and high band gap are: T=1129 °C, flow rate=167 mL min^−1^, molar ratio=5 : 1. The synthesis conditions for the sample with high oxygen content and low band gap are: T=814 °C, flow rate=136 mL min^−1^, molar ratio=2 : 1.

Having examined the goodness of fit of the model predictions equations with the experimental data, we proceed to the final stage of the DOE process (Stage 6, see Figure S1). This involves testing the model prediction equations and validating these predictions with subsequent experiments, which is the third and final objective of our study. To avoid imposing internal bias, we used a random number generator to simulate user‐inputs for the desired oxygen content and band gap. Two extreme values of the oxygen content (2.3 at. % and 9.7 at. %) and band gap (2.80 eV and 1.77 eV) were chosen for model predictions and verification to illustrate the robustness and versatility. The corresponding predicted OB_3_ contents are 6.2 a.u. g^−1^ and 203.4 a.u g^−1^, respectively. Model predictions can only be accurately made within the range of each synthesis parameters. Predicting the properties of BNO for synthesis conditions outside those used to develop the response model and equations would require extrapolation, which naturally introduces error. The model prediction equations yielded the following synthesis conditions for each oxygen content and corresponding band gap: (2.3 at. % corresponding to 2.80 eV: 1129 °C, 167 mL min^−1^, 5 : 1 and 9.7 at. % corresponding to 1.77 eV: 814 °C, 136 mL min^−1^, 2 : 1). Three repeats of each BNO sample were synthesised using the conditions indicated by the model. The experimental oxygen content and band gap of all three repeat samples were measured using XPS and UV‐Vis DRS, respectively. The survey spectrum and fully deconvoluted high resolution B 1s, N 1s and O 1s to determine the chemical composition of the samples is presented in Figures S42 and S43. The absorption spectra used to determine the band gaps of the samples is presented in Figure S44. An average oxygen content and band gap with standard error was calculated and compared to the model predicted values, as shown in Figures 6a and 6b. The average oxygen contents of the low and high samples were determined to be 3.1±0.6 at. % and 8.4 at. ±1.0 at. %, which are in rather good agreement with the model predicted values (2.3 at. % and 9.7 at. %, respectively). The band gaps of the low and high oxygen content samples were determined to be 2.74±0.05 eV and 1.63±0.13 eV, which are also in good agreement with the model predicted values (2.80 eV and 1.77 eV, respectively). The slight deviation in the oxygen content predictions can be explained by considering the leverage plots comparing the model predicted values to experimental values in Figure S39. The confidence bands for the oxygen content model predictions are wider than those for the band gap. This shows that there is more uncertainty in the model predictions for the oxygen content than the band gap. This could be improved by either collecting mid‐range band gap data points or more data points at the extremities, namely higher oxygen content values. Overall, we note that while the experimental values for the oxygen content and band gap are very close to the predicted values, they are not within the predicted intervals. There are several possible reasons for this observation. The first one is that the model provides a 95 % confidence interval. The second is that the experimental errors on the estimation of the band gap and oxygen content are larger than what is predicted. The third is that additional factors influencing the reactions have not been considered in the model (e. g., how well mixed the precursors were) and have contributed to the errors observed. Nevertheless, the results in Figure 6 highlights the ability of the model equations presented herein to generally predict the oxygen content and band gap of BNO.

## Conclusions

In summary, our systematic study has experimentally tested and confirmed the theoretical DFT predictions of Weng *et al*.,^[18]^ *i.e*., the band gap of BNO materials can be varied and lowered by tuning the oxygen content, and in doing so the band gap. This provides scope to enhance the light harvesting, and hence performance, of BN‐based photocatalysts for solar fuels synthesis. Using the experimental data, we employed a response surface design and DOE approach. Through this approach, we identified the key synthesis parameters influencing the chemical, magnetic and optoelectronic properties of BNO. Further, we developed model prediction equations relating these properties to the multivariable synthesis parameter space. A DOE modelling approach was adopted with detailed statistical analyses and hypothesis tests conducted to identify outliers, influential data points and assess the goodness of fit of the model predictions to the experimental data. Using the model equations, predictions of the chemistry and optoelectronic properties of BNO were achieved and validated experimentally. The methodology used in this study can be extended to other forms of doped BN, i. e. doping with heteroatoms such as S and P. This provides scope for systematic tailoring of the photochemistry of BN materials towards given reactions, e. g. photocatalytic CO_2_ reduction and H_2_ generation.

## Supporting Information

Characterisation analyses (i. e. XRD, FTIR, XPS, DRS UV Vis) and DOE model statistics.

## Conflict of interest

The authors declare no conflict of interest.

1

## Supporting information

As a service to our authors and readers, this journal provides supporting information supplied by the authors. Such materials are peer reviewed and may be re‐organized for online delivery, but are not copy‐edited or typeset. Technical support issues arising from supporting information (other than missing files) should be addressed to the authors.

Supporting Information

## Data Availability

The data that support the findings of this study are available in the supplementary material of this article.
