# Supplementary material for: A Response Surface Model to Predict and Experimentally Tune the Chemical, Magnetic and Optoelectronic Properties of Oxygen‐Doped Boron Nitride
Source: Chemphyschem. 2022 May 19;23(13):e202100854. doi: 10.1002/cphc.202100854 (PMC9400848; doi:10.1002/cphc.202100854)
Supplement: Supplementary file 1 — Supporting Information [file CPHC-23-0-s001.pdf]

ChemPhysChem

Supporting Information

A Response Surface Model to Predict and Experimentally Tune the Chemical, Magnetic and Optoelectronic Properties of Oxygen-Doped Boron Nitride**

Ravi B. Shankar[+], Elan D. R. Mistry[+], Daphné Lubert-Perquel, Irena Nevjestic, Sandrine Heutz, and Camille Petit*

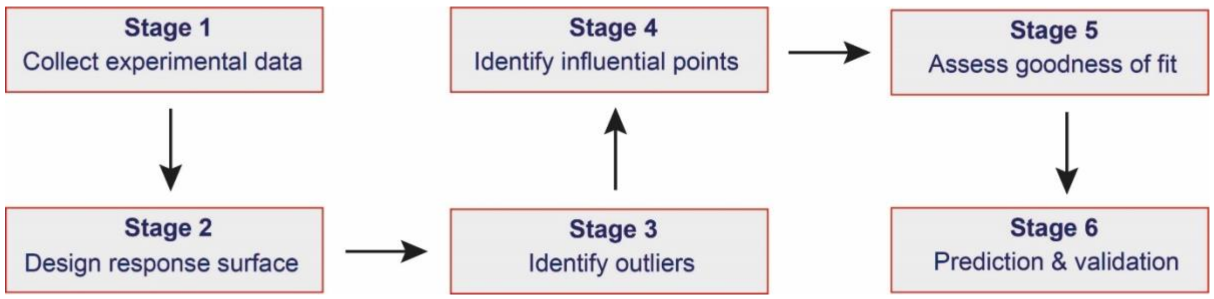

Figure S1. Flowchart illustrating the stages of the DOE and response surface design approach employed in this study.

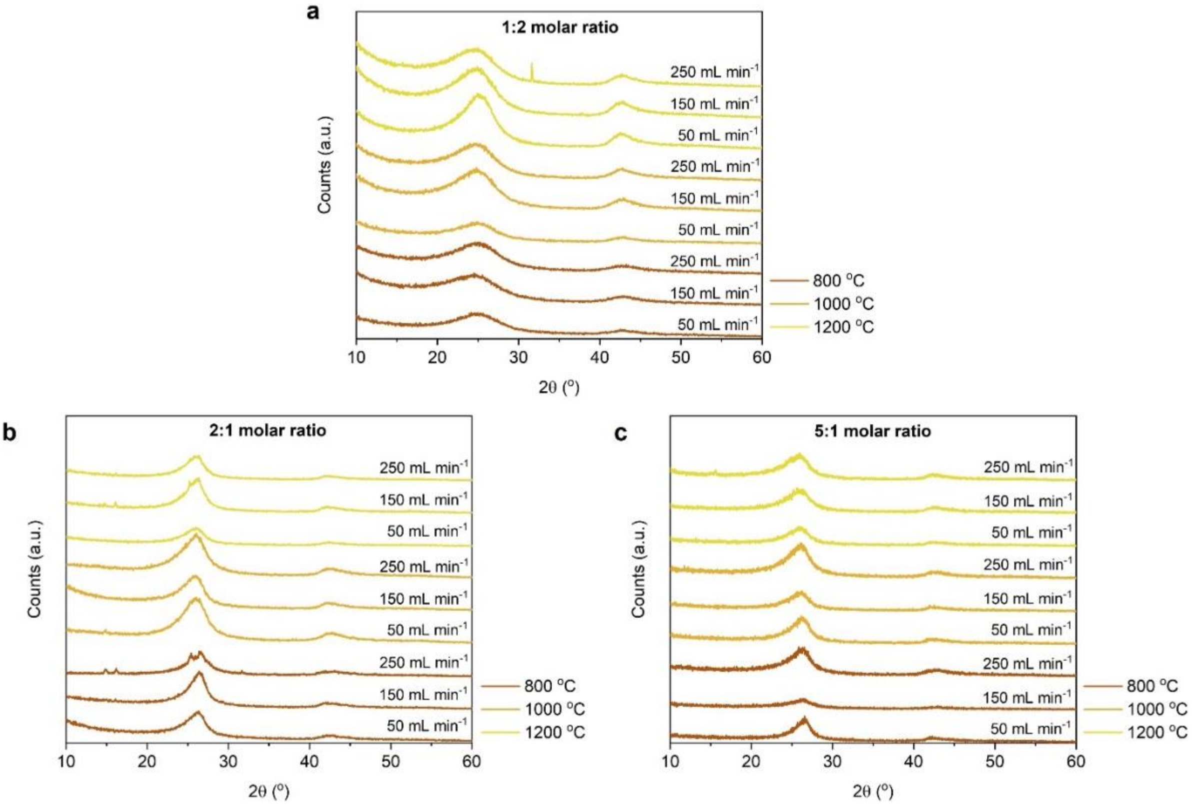

Figure S2. Powder XRD patterns for all the BNO materials investigated in this study.

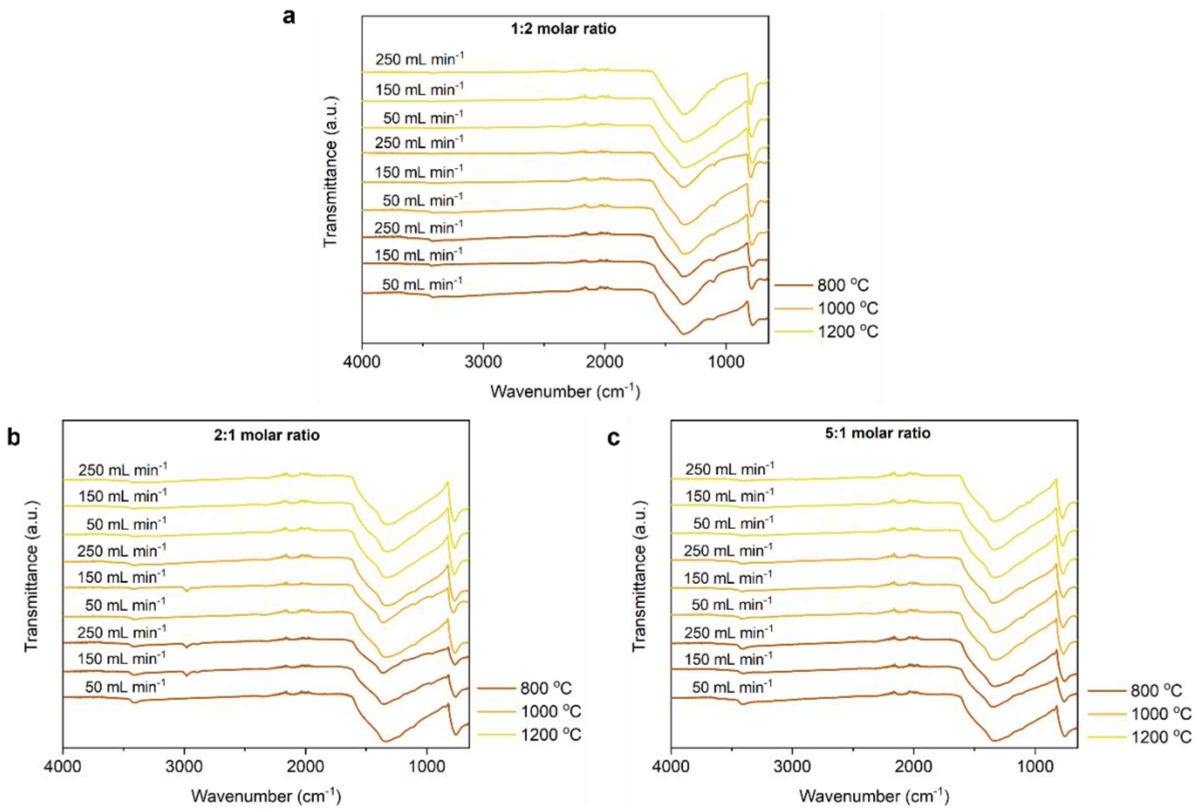

Figure S3. FT-IR spectra for all the BNO materials investigated in this study.

Table S1. Atomic compositions obtained through XPS analysis for all the materials investigated in this study.

Sample	Ratio (-)	Temperature (ºC)	Flowrate (mL min^{-1})	B (at. %)	C (at. %)	N (at. %)	O (at. %)
1	1:2	800	50	43.07	13.52	32.79	10.62
2	1:2	800	150	46.63	7.84	37.55	7.98
3	1:2	800	250	47.73	6.20	38.55	7.50
4	1:2	1000	50	48.70	5.62	38.67	7.01
5	1:2	1000	150	50.58	3.94	42.01	3.49
6	1:2	1000	250	50.57	4.10	42.40	2.93
7	1:2	1200	50	51.06	3.83	42.18	2.92
8	1:2	1200	150	49.50	6.37	40.69	3.44
9	1:2	1200	250	50.70	3.89	42.02	3.39
10	2:1	800	50	44.67	10.24	34.5	10.59
11	2:1	800	150	44.08	11.05	34.82	10.05
12	2:1	800	250	46.65	7.30	35.74	10.31
13	2:1	1000	50	47.28	7.95	37.40	7.38
14	2:1	1000	150	50.97	3.21	42.23	3.60
15	2:1	1000	250	51.14	2.90	42.91	3.05
16	2:1	1200	50	51.90	2.46	42.75	2.89
17	2:1	1200	150	52.36	1.98	43.52	2.13
18	2:1	1200	250	51.92	2.34	43.47	2.27
19	5:1	800	50	47.11	5.04	35.19	12.67
20	5:1	800	150	42.68	12.72	30.83	13.77
21	5:1	800	250	47.37	5.83	37.85	8.96
22	5:1	1000	50	51.41	2.54	41.8	4.27
23	5:1	1000	150	51.59	2.3	43.19	2.93
24	5:1	1000	250	51.51	2.18	42.15	4.16
25	5:1	1200	50	52.03	2.3	42.85	2.82
26	5:1	1200	150	52.29	1.98	43.57	2.15
27	5:1	1200	250	52.27	2.32	42.76	2.64

Table S2 Standard error in the oxygen content measurement using XPS through repeat measurements of a low, and high oxygen content sample.

Sample	O (at. %) (Run 1)	O (at. %) (Run 2)	O (at. %) (Run 3)	Standard deviation (at. %)
13	7.38	8.41	8.60	0.66
27	2.64	2.41	2.15	0.25

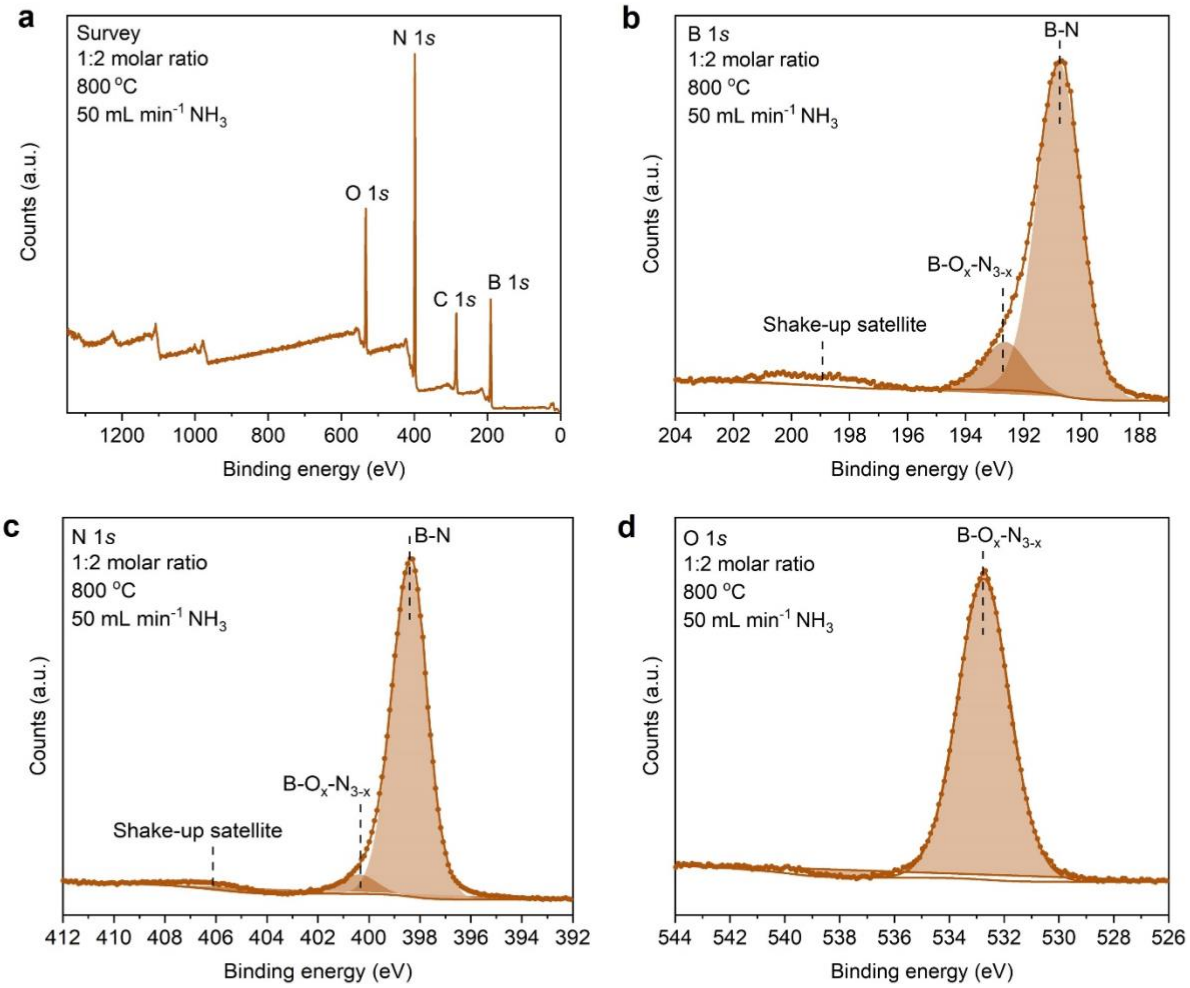

Figure S4. Chemical composition and states in BNO samples. (1:2 molar ratio, 800 °C, 50 mL min^{-1} NH_3) - Survey spectrum and high resolution XPS core level spectra for B 1*s*, N 1*s* and O 1*s* with the key characteristic peaks and corresponding binding energies highlighted.

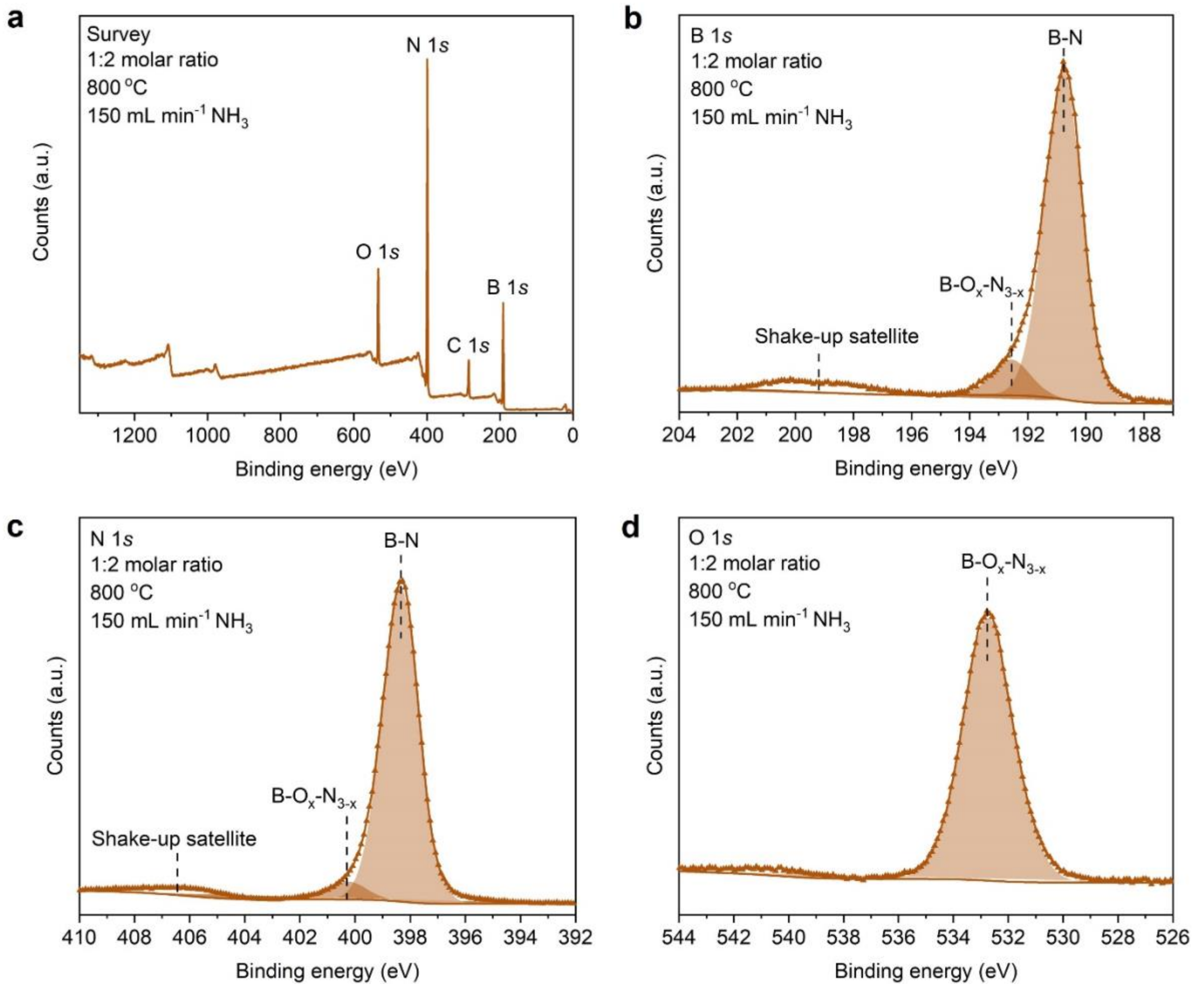

Figure S5. Chemical composition and states in BNO samples. (1:2 molar ratio, 800 °C, 150 mL min^{-1} NH_3) - Survey spectrum and high resolution XPS core level spectra for B 1*s*, N 1*s* and O 1*s* with the key characteristic peaks and corresponding binding energies highlighted.

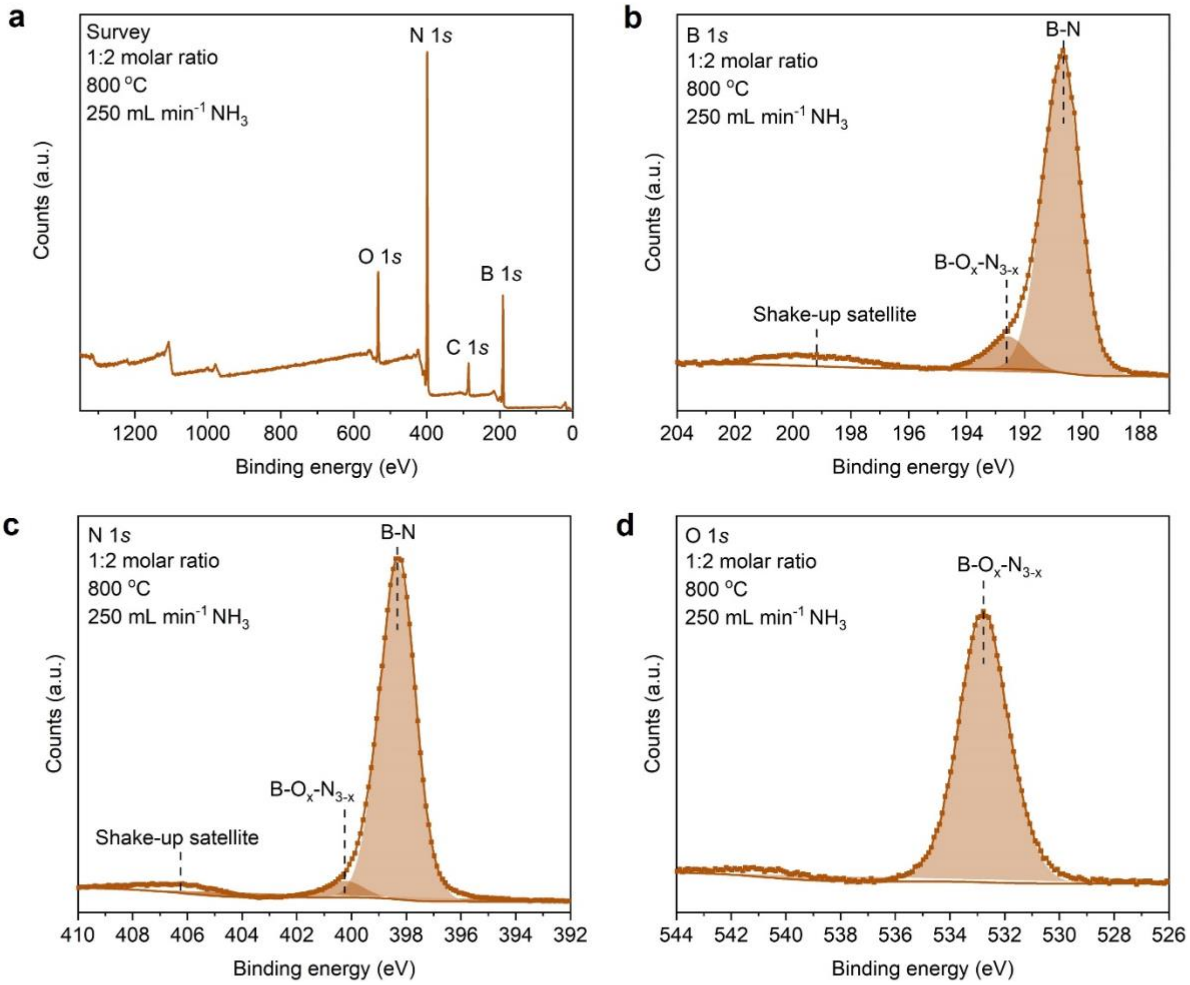

Figure S6. Chemical composition and states in BNO samples. (1:2 molar ratio, 800 ºC, 250 mL min^{-1} NH$_3$) - Survey spectrum and high resolution XPS core level spectra for B 1*s*, N 1*s* and O 1*s* with the key characteristic peaks and corresponding binding energies highlighted.

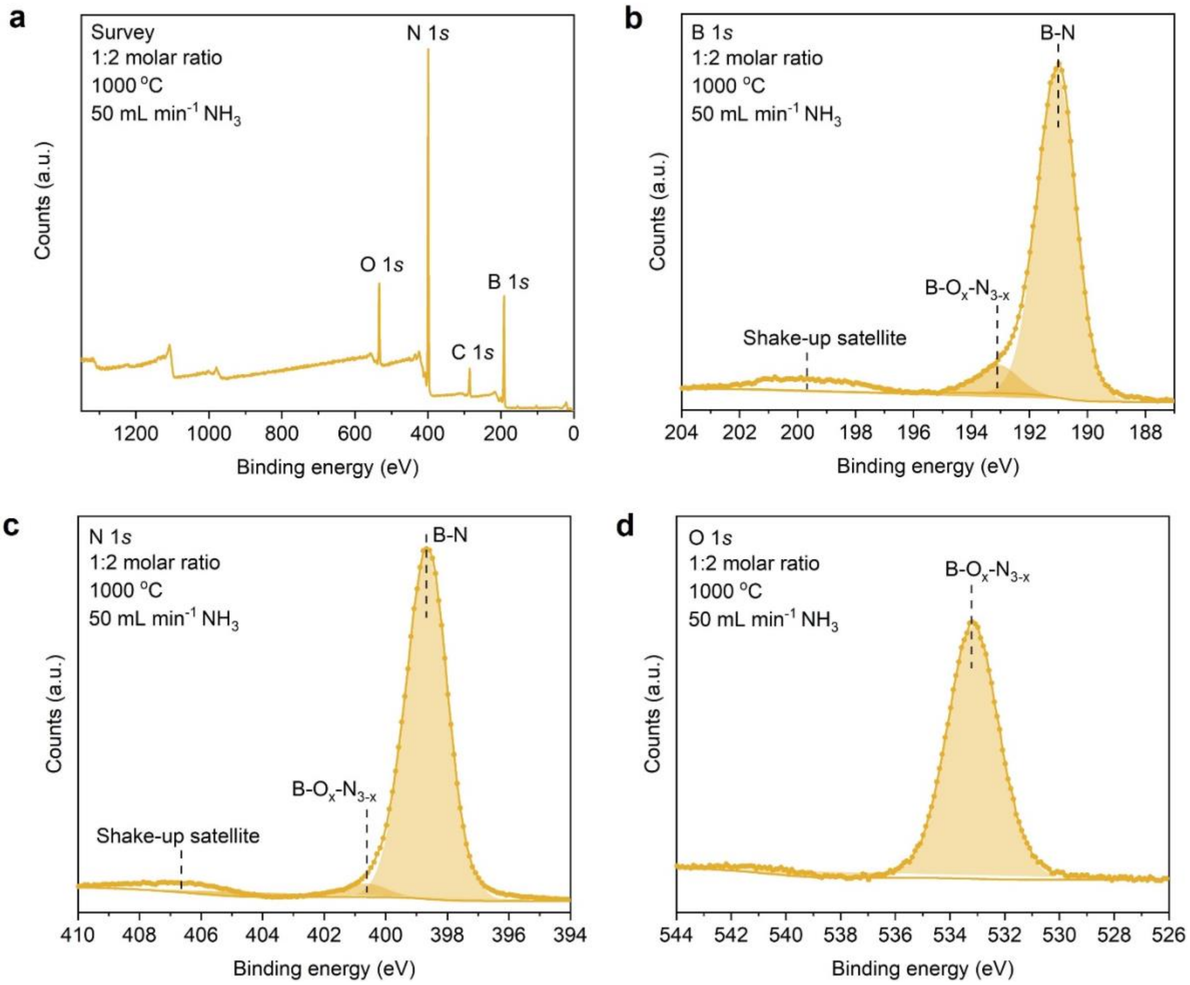

Figure S7. Chemical composition and states in BNO samples. (1:2 molar ratio, 1000 °C, 50 mL min^{-1} NH_3) - Survey spectrum and high resolution XPS core level spectra for B 1*s*, N 1*s* and O 1*s* with the key characteristic peaks and corresponding binding energies highlighted.

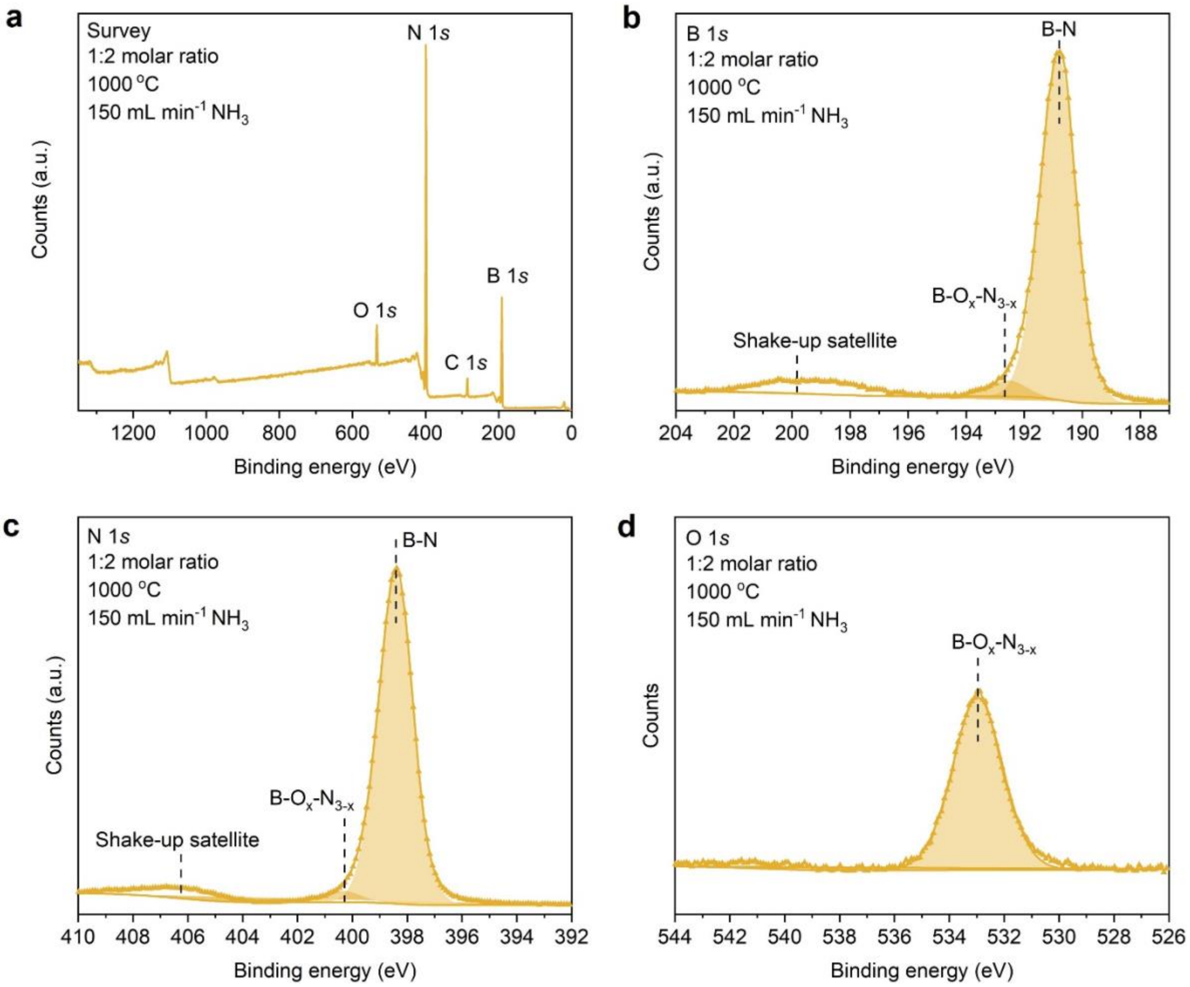

Figure S8. Chemical composition and states in BNO samples. (1:2 molar ratio, 1000 °C, 150 mL min^{-1} NH_3) - Survey spectrum and high resolution XPS core level spectra for B 1*s*, N 1*s* and O 1*s* with the key characteristic peaks and corresponding binding energies highlighted.

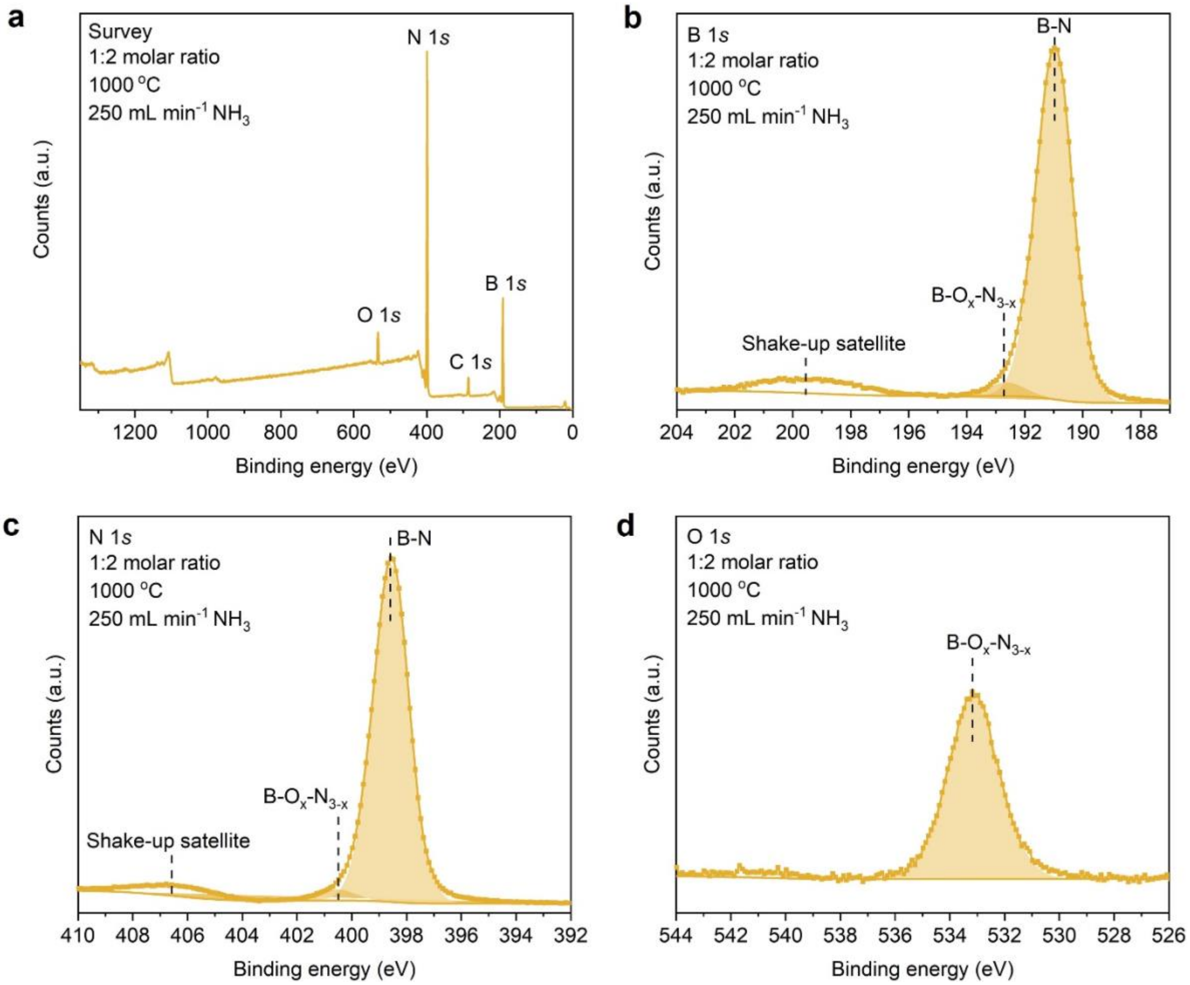

Figure S9. Chemical composition and states in BNO samples. (1:2 molar ratio, 1000 °C, 250 mL min^{-1} NH_3) - Survey spectrum and high resolution XPS core level spectra for B 1*s*, N 1*s* and O 1*s* with the key characteristic peaks and corresponding binding energies highlighted.

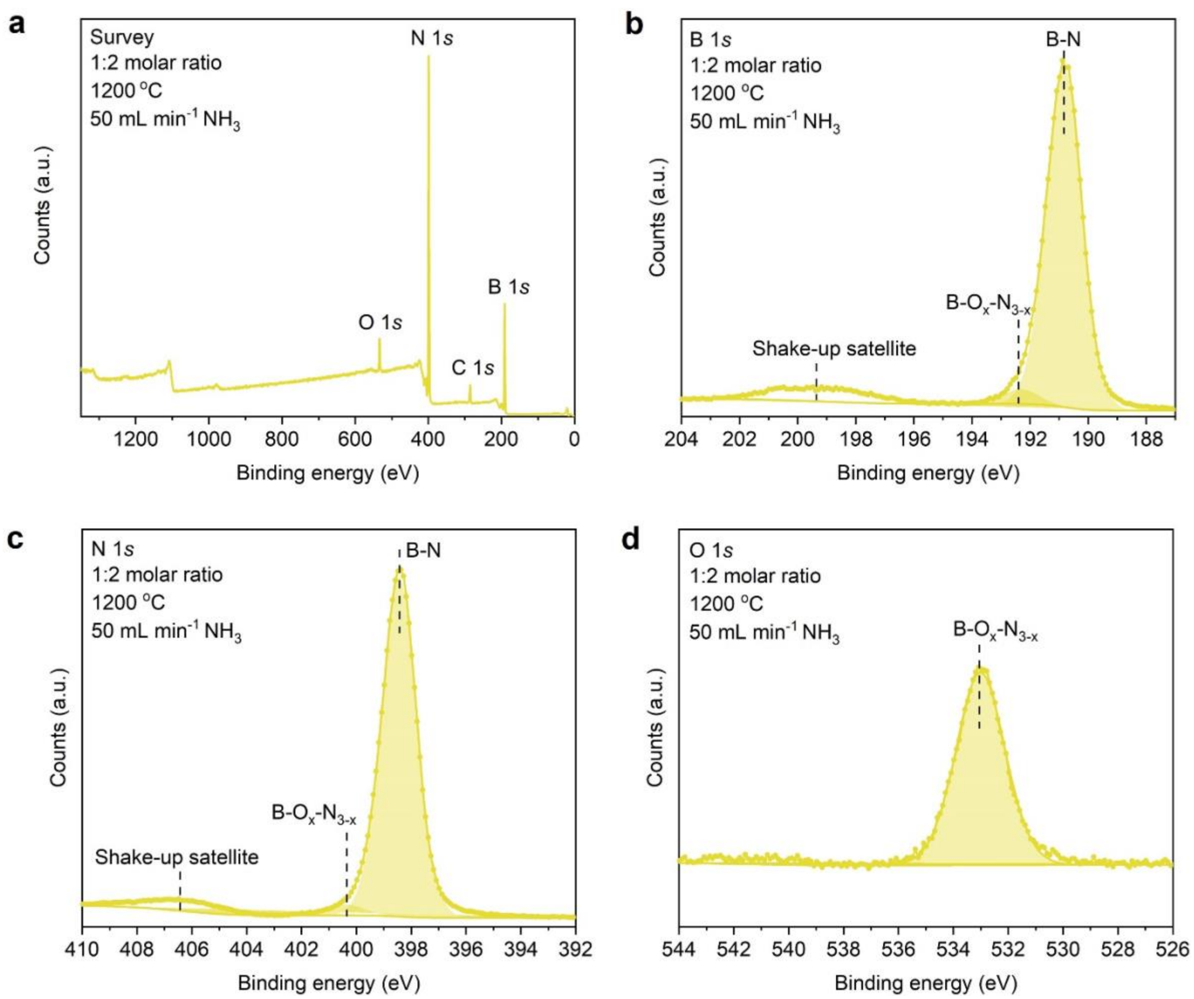

Figure S10. Chemical composition and states in BNO samples. (1:2 molar ratio, 1200 ºC, 50 mL min^{-1} NH_3) - Survey spectrum and high resolution XPS core level spectra for B 1*s*, N 1*s* and O 1*s* with the key characteristic peaks and corresponding binding energies highlighted.

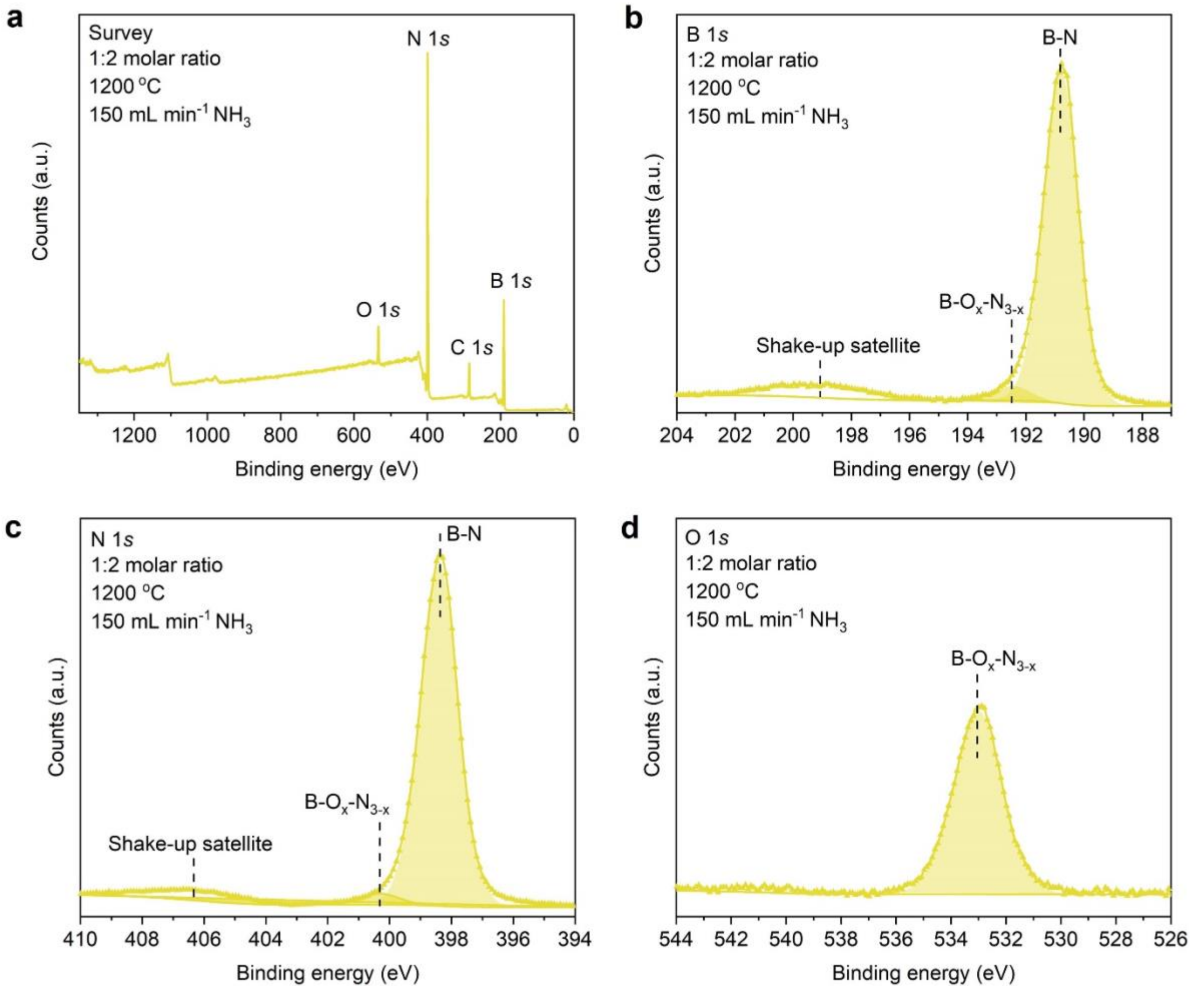

Figure S11. Chemical composition and states in BNO samples. (1:2 molar ratio, 1200 ºC, 150 mL min^{-1} NH_3) - Survey spectrum and high resolution XPS core level spectra for B 1*s*, N 1*s* and O 1*s* with the key characteristic peaks and corresponding binding energies highlighted for all the oxygen-doped BN materials investigated in this study.

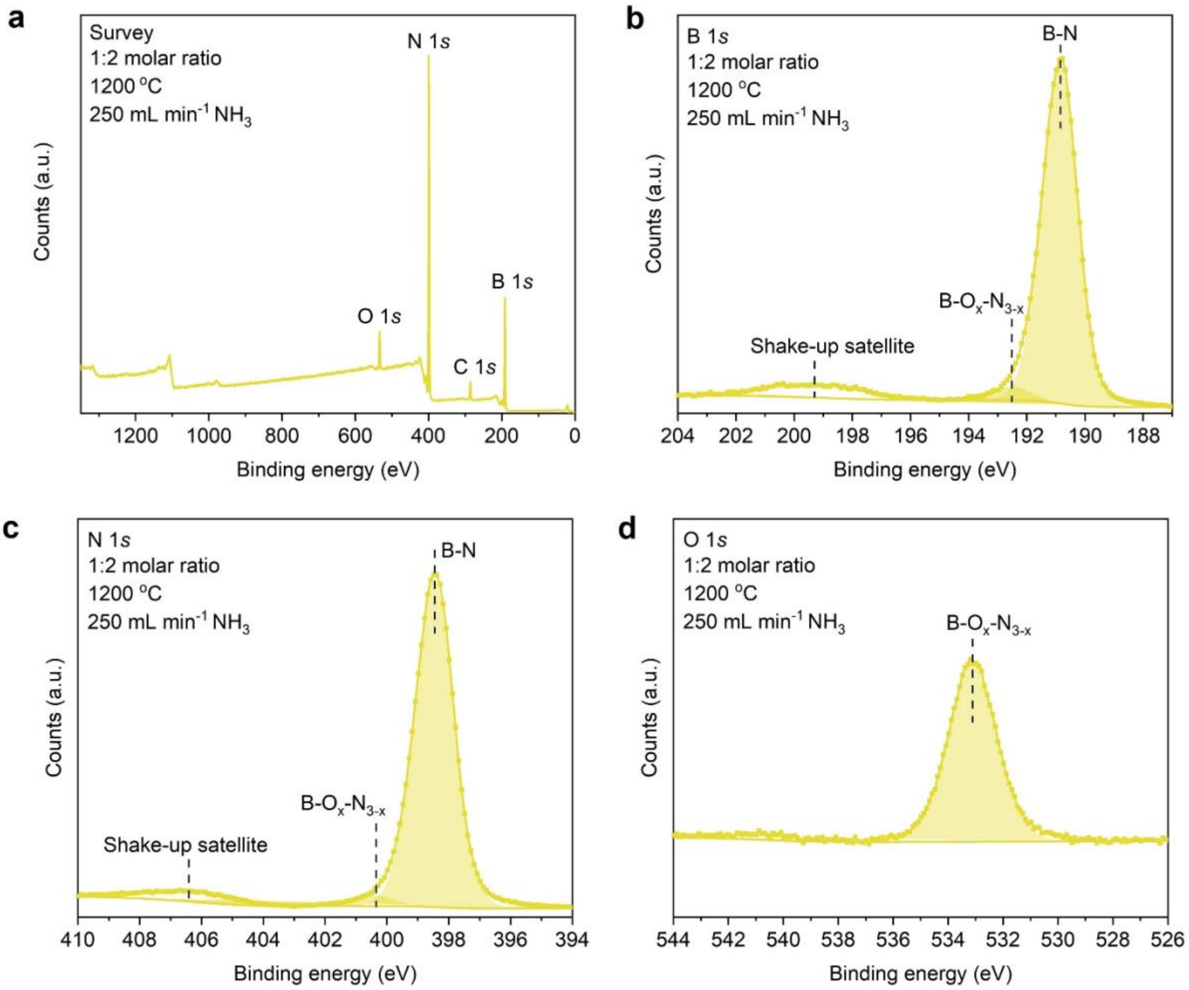

Figure S12. Chemical composition and states in BNO samples. (1:2 molar ratio, 1200 ºC, 250 mL min^{-1} NH_3) - Survey spectrum and high resolution XPS core level spectra for B 1*s*, N 1*s* and O 1*s* with the key characteristic peaks and corresponding binding energies highlighted for all the oxygen-doped BN materials investigated in this study.

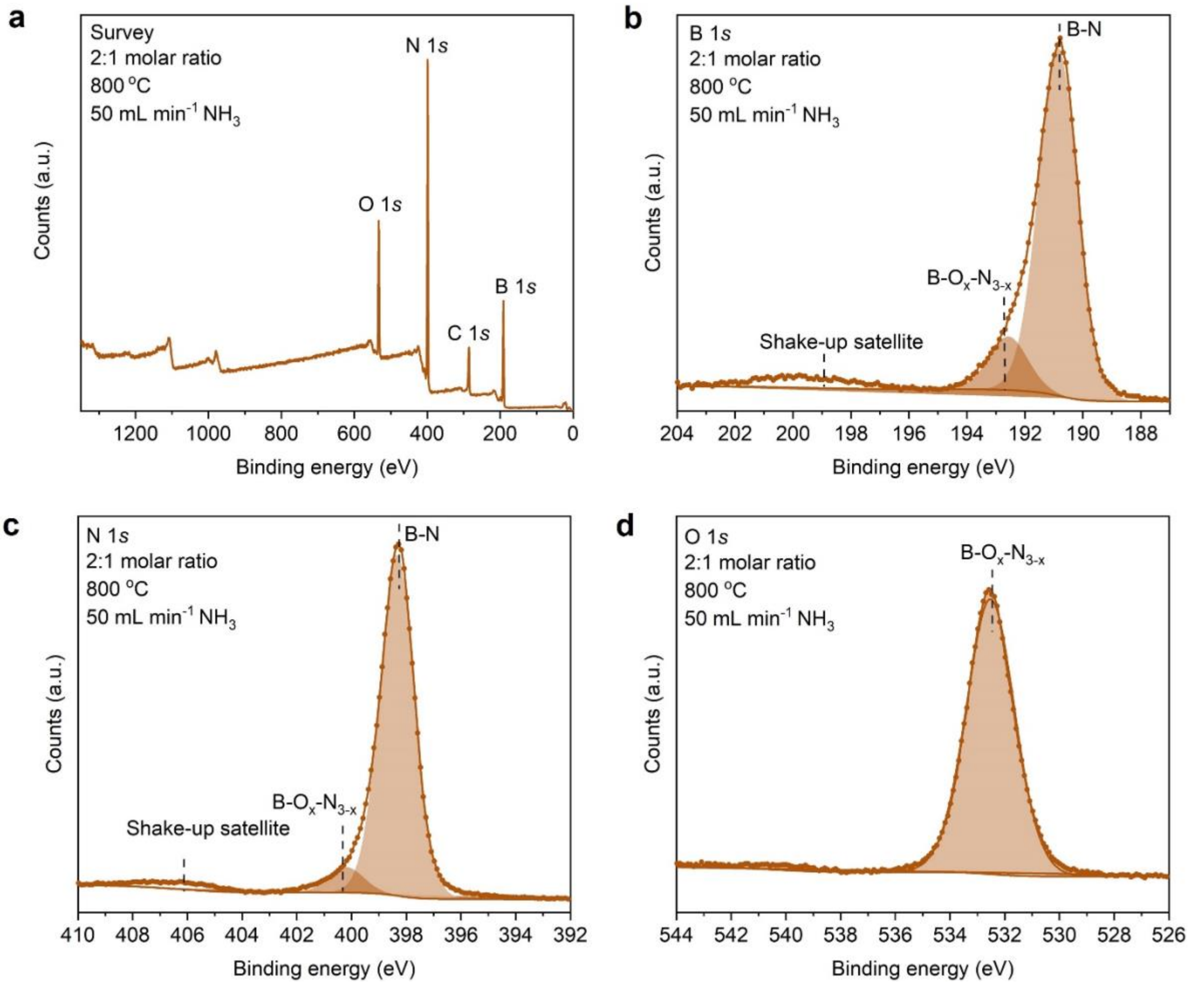

Figure S13. Chemical composition and states in BNO samples. (2:1 molar ratio, 800 ºC, 50 mL min^{-1} NH_3) - Survey spectrum and high resolution XPS core level spectra for B 1*s*, N 1*s* and O 1*s* with the key characteristic peaks and corresponding binding energies highlighted for all the oxygen-doped BN materials investigated in this study.

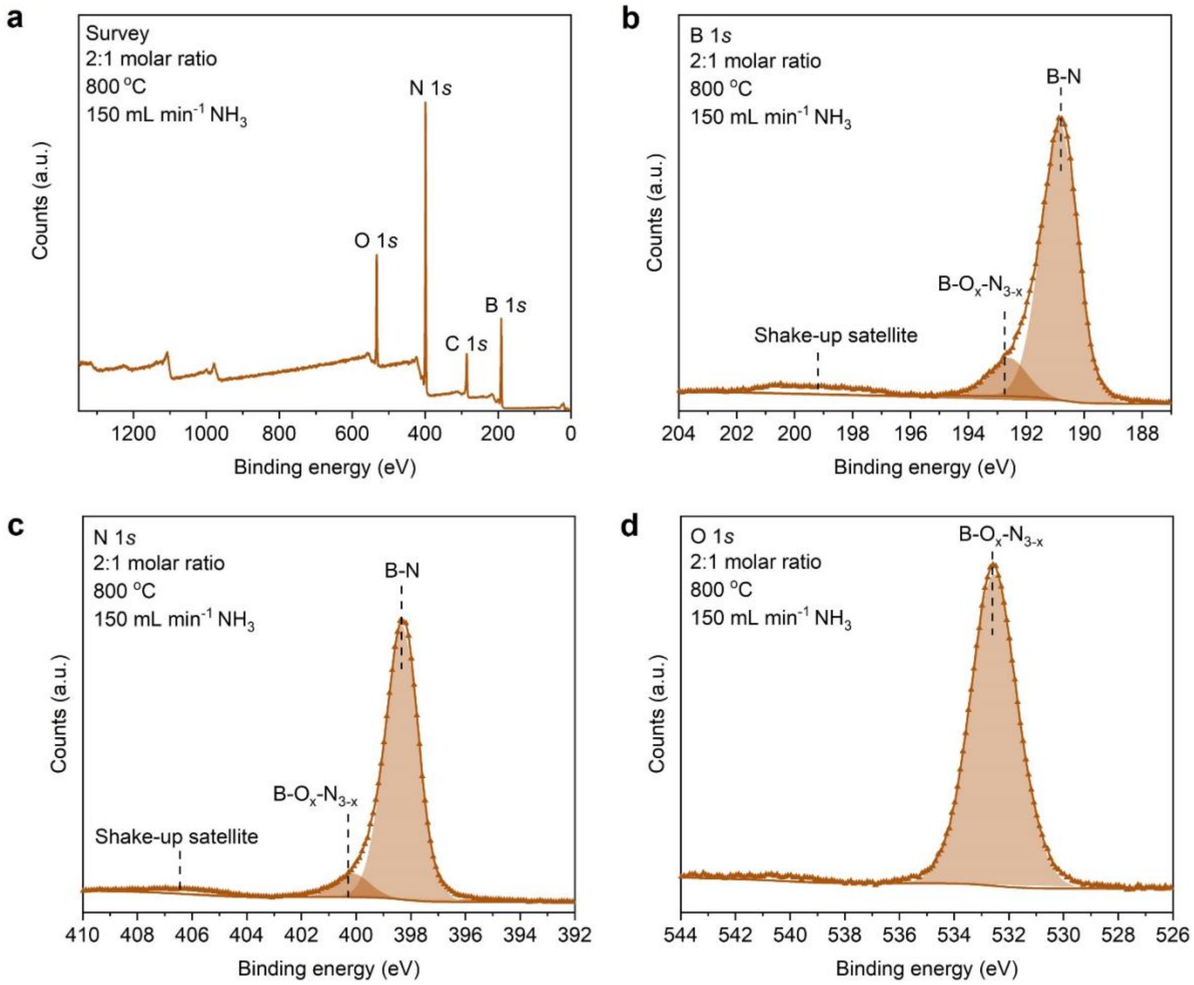

Figure S14. Chemical composition and states in BNO samples. (2:1 molar ratio, 800 °C, 150 mL min^{-1} NH_3) - Survey spectrum and high resolution XPS core level spectra for B 1*s*, N 1*s* and O 1*s* with the key characteristic peaks and corresponding binding energies highlighted for all the oxygen-doped BN materials investigated in this study.

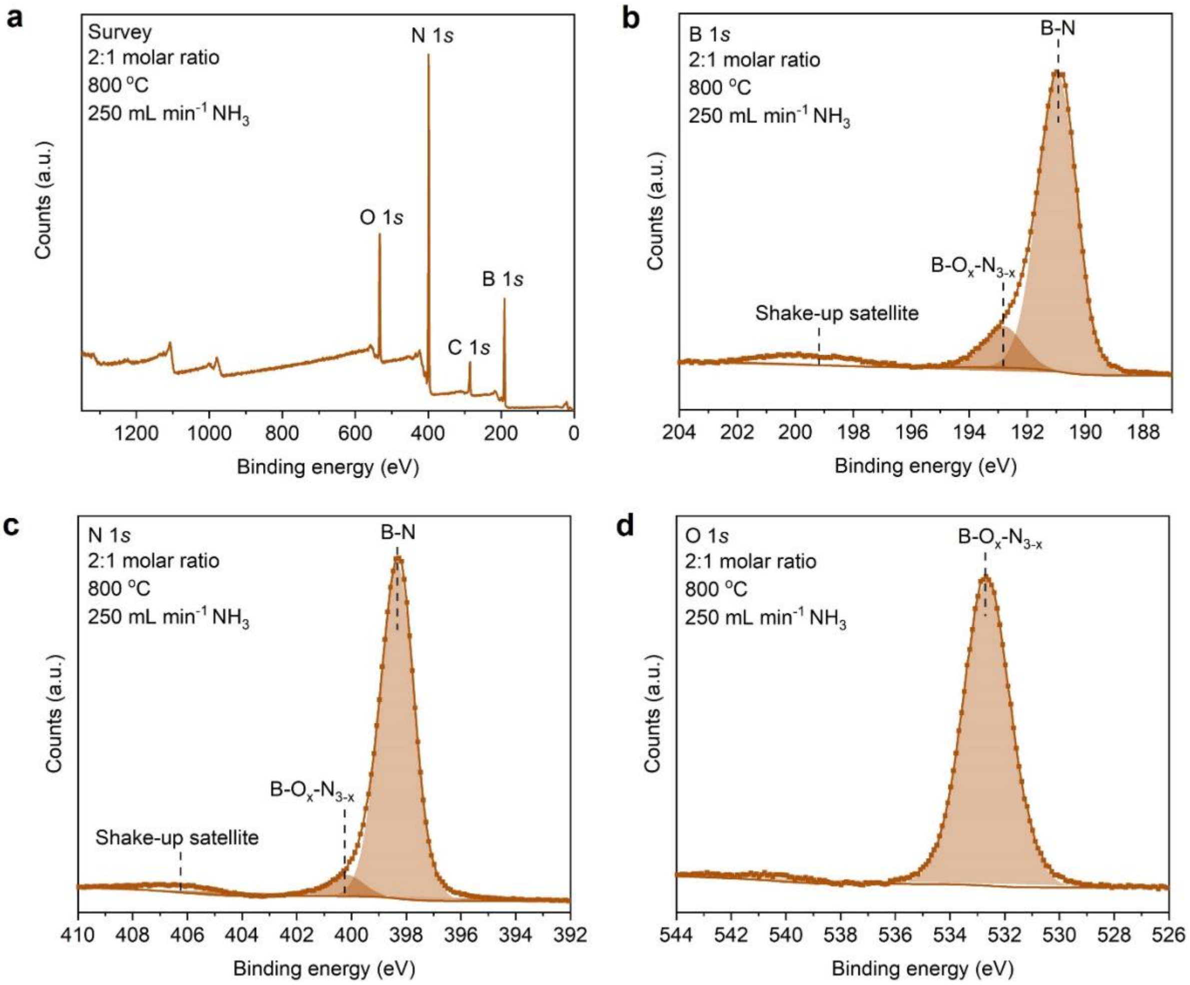

Figure S15. Chemical composition and states in BNO samples. (2:1 molar ratio, 800 °C, 250 mL min^{-1} NH_3) - Survey spectrum and high resolution XPS core level spectra for B 1*s*, N 1*s* and O 1*s* with the key characteristic peaks and corresponding binding energies highlighted.

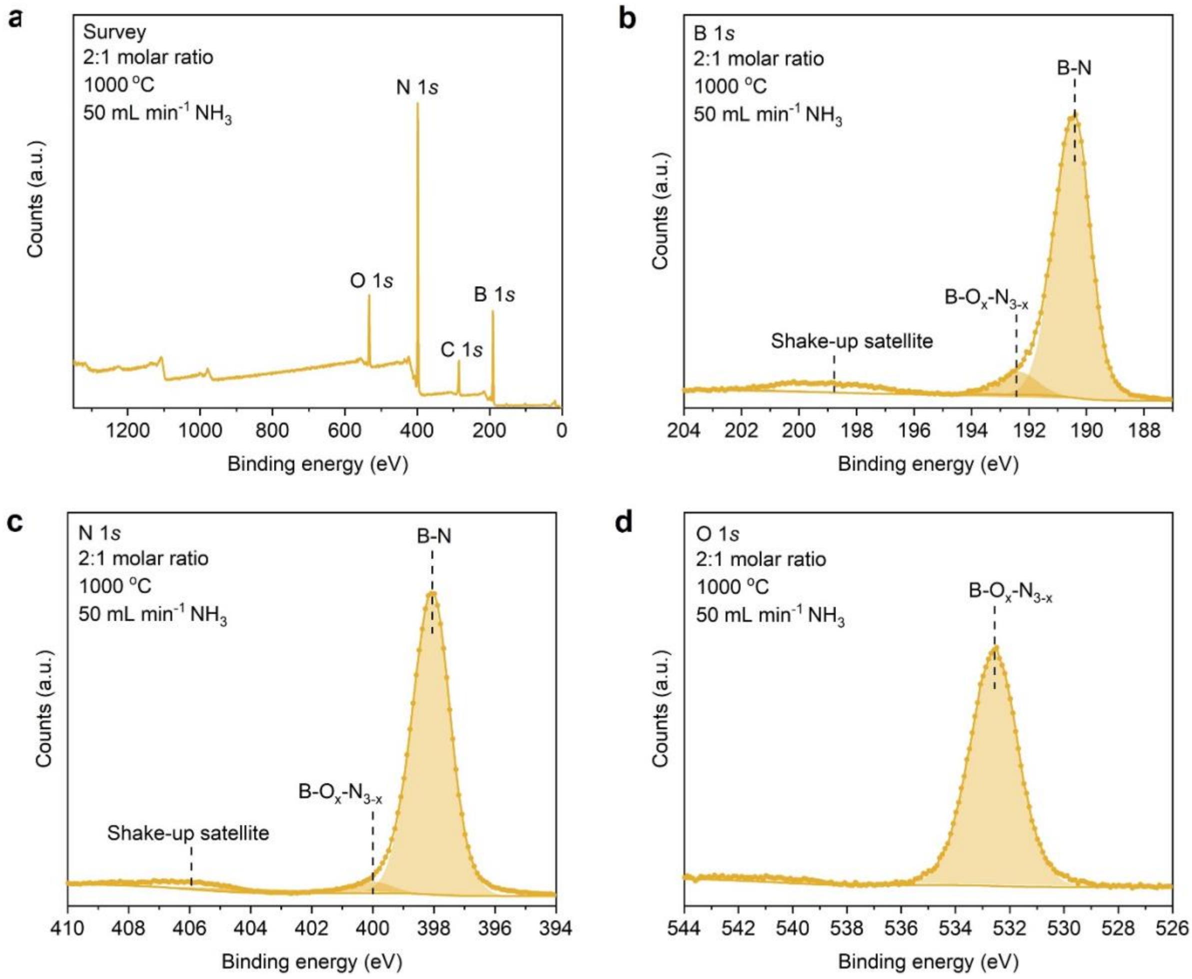

Figure S16. Chemical composition and states in BNO samples. (2:1 molar ratio, 1000 ºC, 50 mL min^{-1} NH_3) - Survey spectrum and high resolution XPS core level spectra for B 1*s*, N 1*s* and O 1*s* with the key characteristic peaks and corresponding binding energies highlighted.

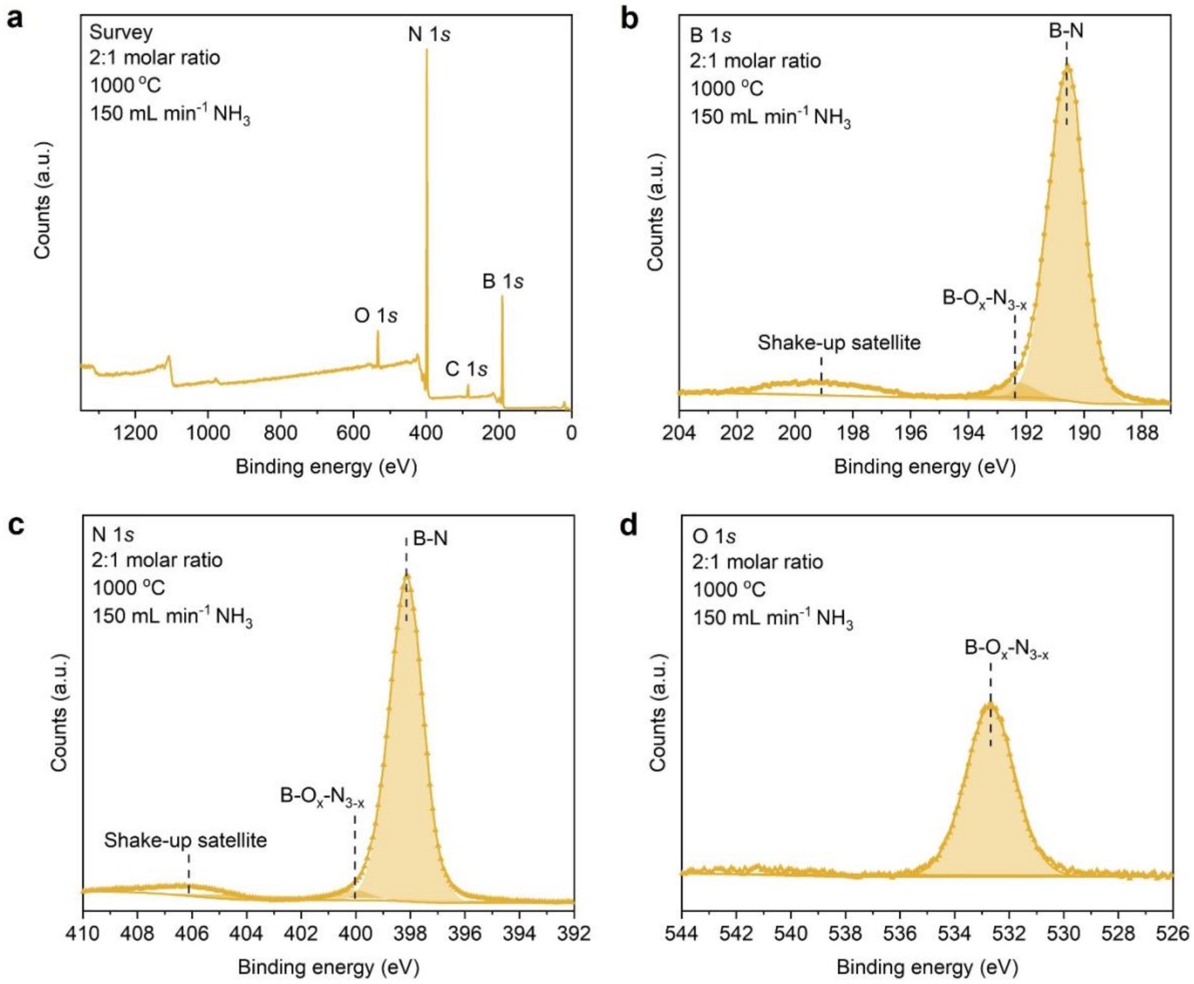

Figure S17. Chemical composition and states in BNO samples. (2:1 molar ratio, 1000 ºC, 150 mL min^{-1} NH_3) - Survey spectrum and high resolution XPS core level spectra for B 1*s*, N 1*s* and O 1*s* with the key characteristic peaks and corresponding binding energies highlighted.

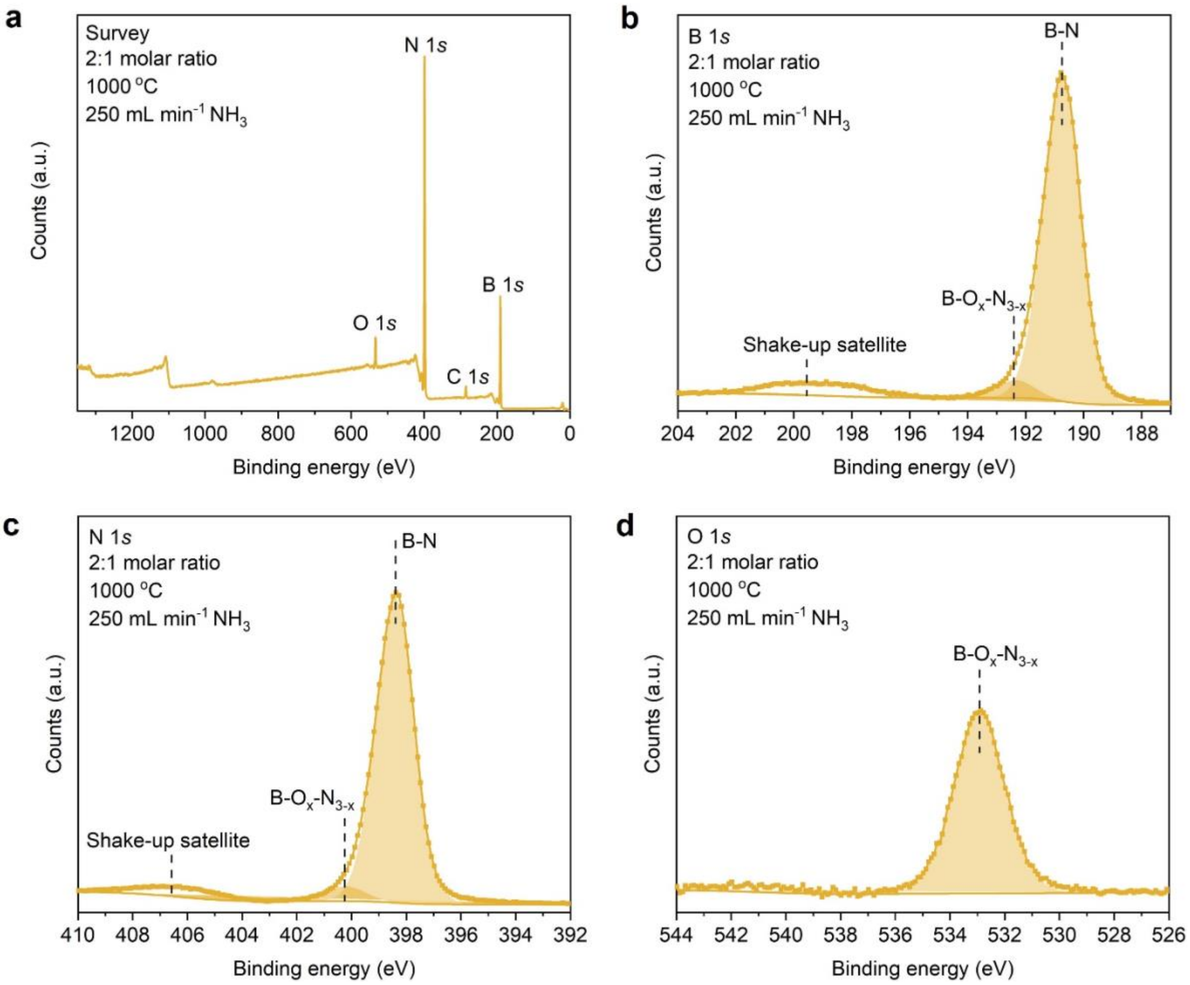

Figure S18. Chemical composition and states in BNO samples. (2:1 molar ratio, 1000 °C, 250 mL min^{-1} NH_3) - Survey spectrum and high resolution XPS core level spectra for B 1*s*, N 1*s* and O 1*s* with the key characteristic peaks and corresponding binding energies highlighted.

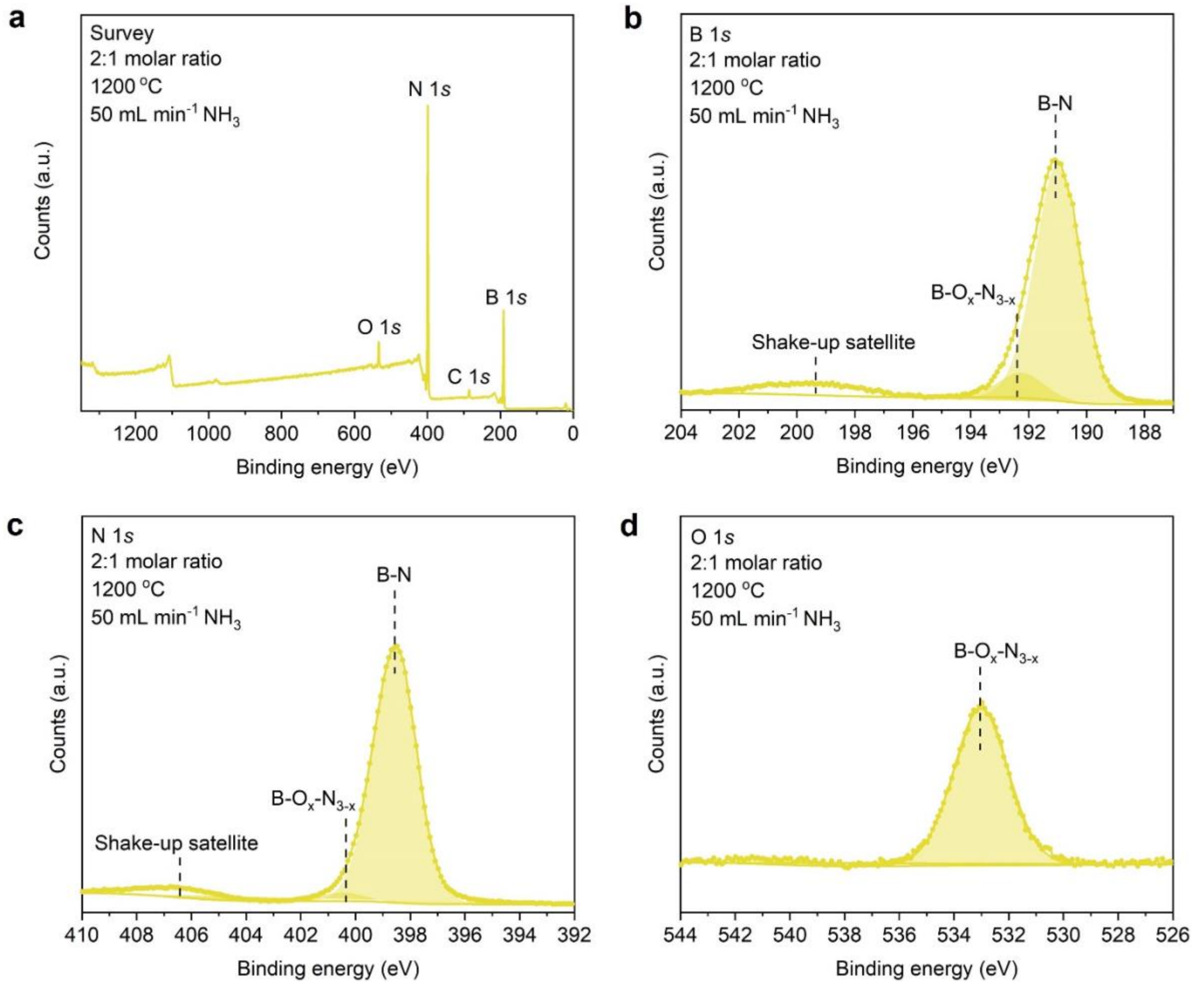

Figure S19. Chemical composition and states in BNO samples. (2:1 molar ratio, 1200 ºC, 50 mL min^{-1} NH_3) - Survey spectrum and high resolution XPS core level spectra for B 1*s*, N 1*s* and O 1*s* with the key characteristic peaks and corresponding binding energies highlighted.

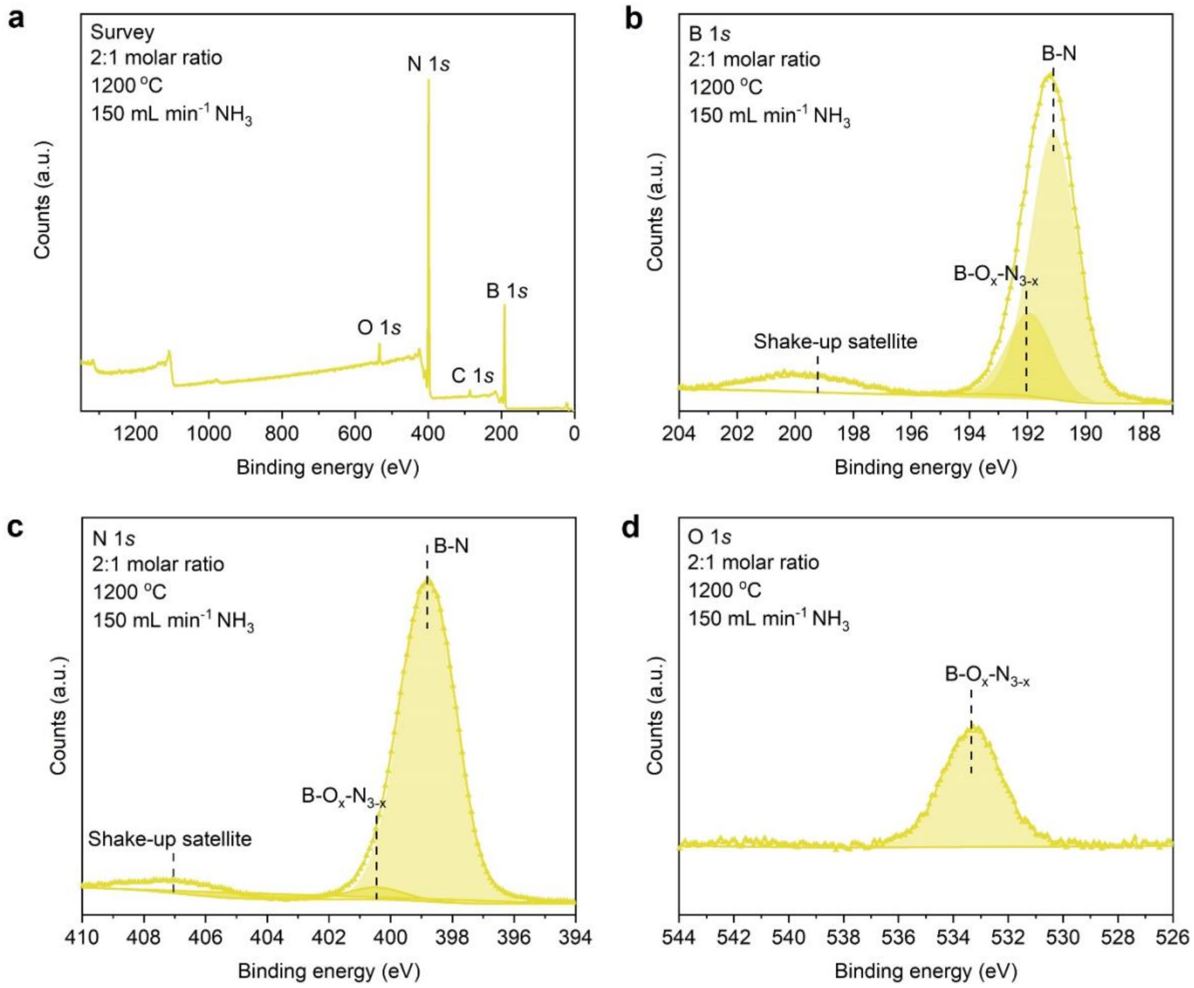

Figure S20. Chemical composition and states in BNO samples. (2:1 molar ratio, 1200 ºC, 150 mL min^{-1} NH_3) - Survey spectrum and high resolution XPS core level spectra for B 1*s*, N 1*s* and O 1*s* with the key characteristic peaks and corresponding binding energies highlighted.

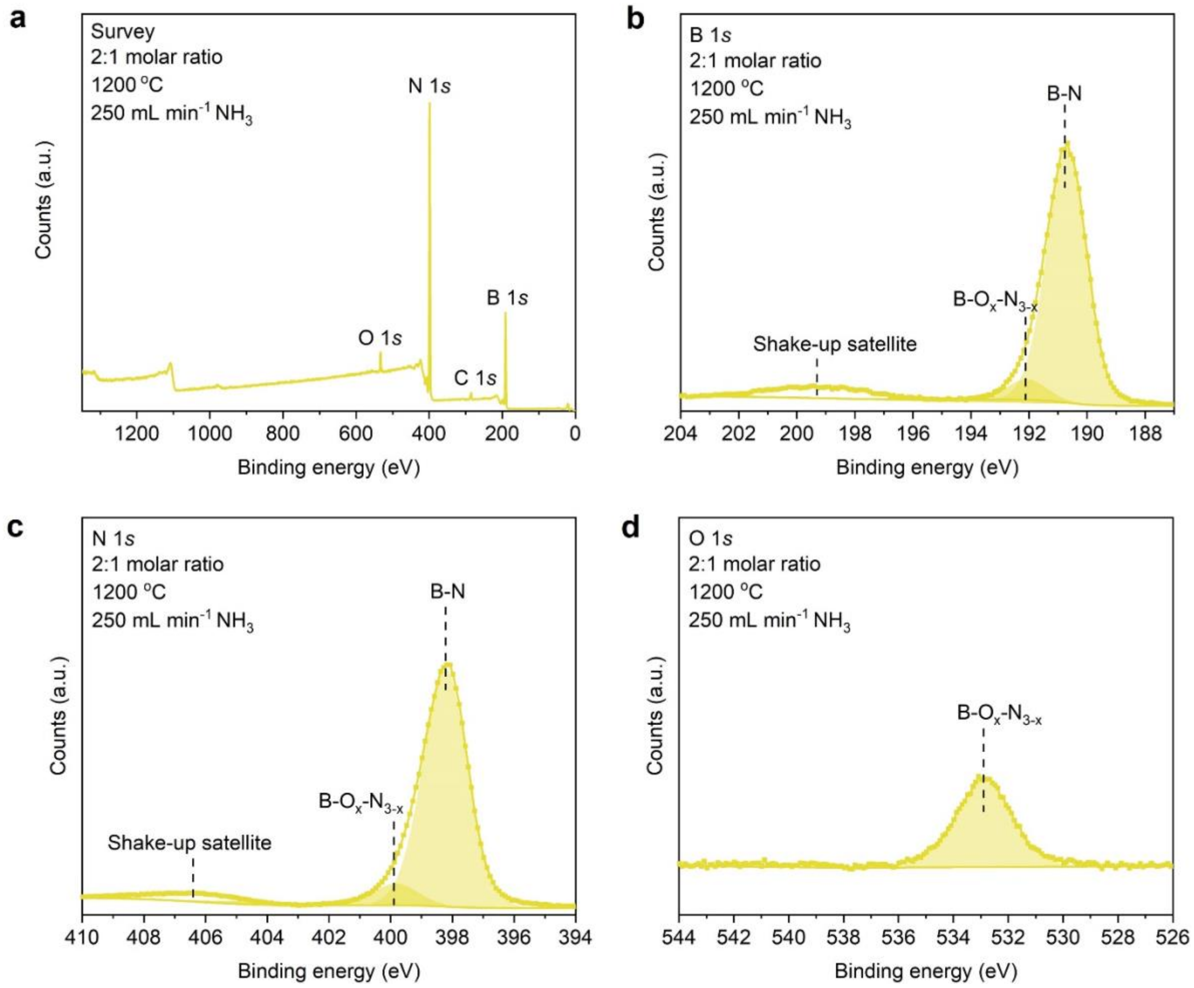

Figure S21. Chemical composition and states in BNO samples. (2:1 molar ratio, 1200 ºC, 250 mL min^{-1} NH_3) - Survey spectrum and high resolution XPS core level spectra for B 1*s*, N 1*s* and O 1*s* with the key characteristic peaks and corresponding binding energies highlighted.

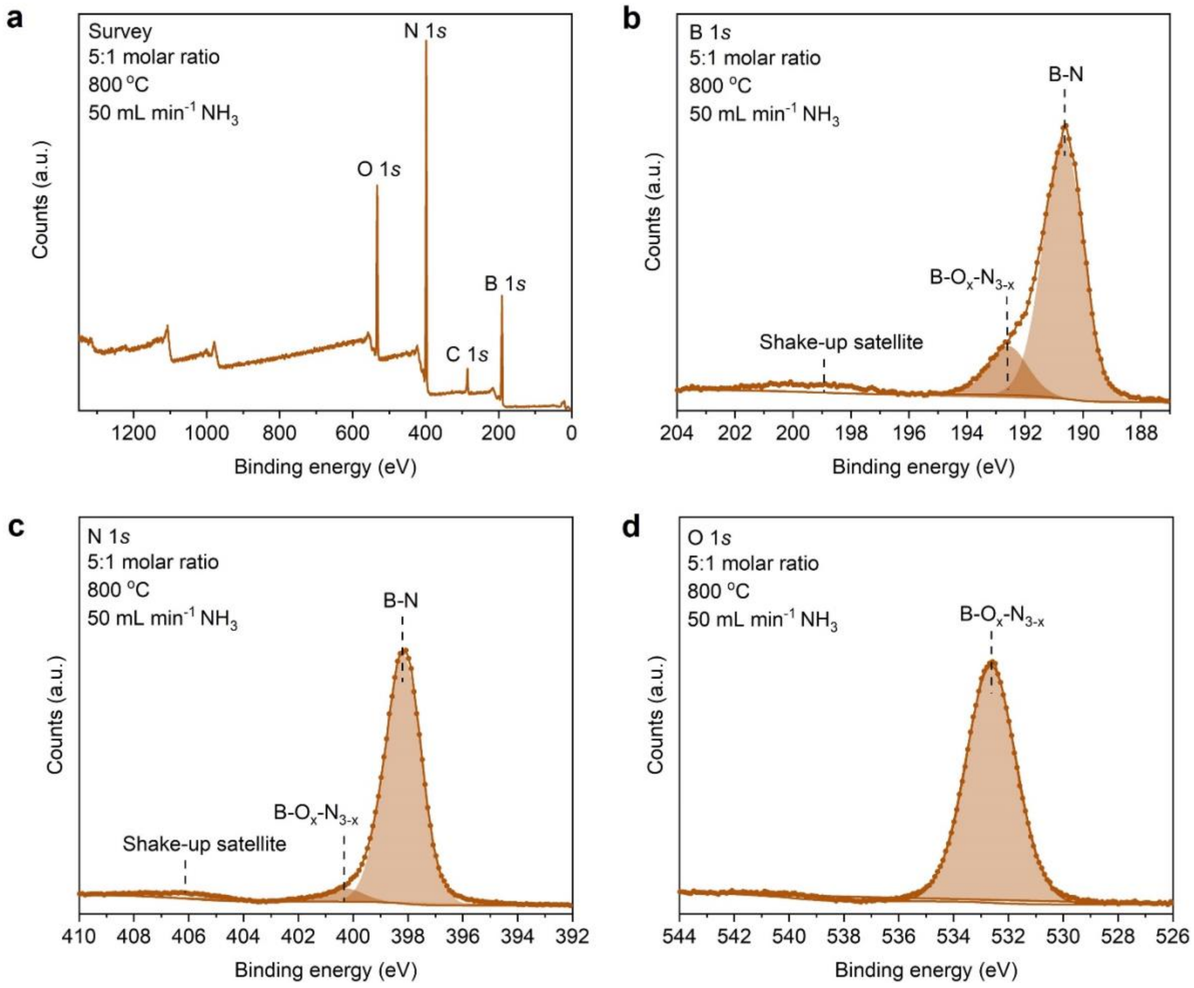

Figure S22. Chemical composition and states in BNO samples. (5:1 molar ratio, 800 °C, 50 mL min^{-1} NH_3) - Survey spectrum and high resolution XPS core level spectra for B 1*s*, N 1*s* and O 1*s* with the key characteristic peaks and corresponding binding energies highlighted.

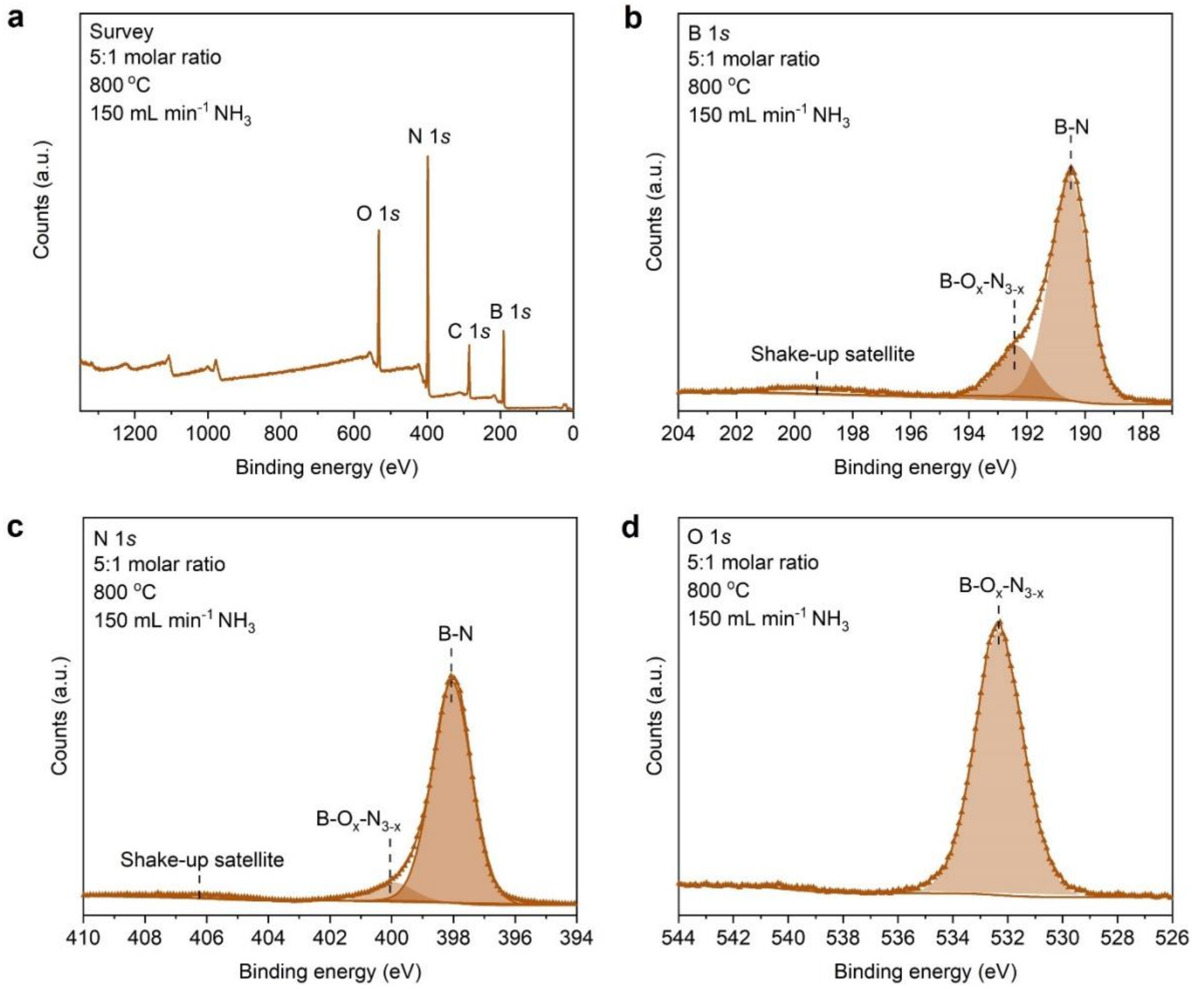

Figure S23. Chemical composition and states in BNO samples. (5:1 molar ratio, 800 ^{o}C, 150 mL min^{-1} NH$_3$) - Survey spectrum and high resolution XPS core level spectra for B 1*s*, N 1*s* and O 1*s* with the key characteristic peaks and corresponding binding energies highlighted.

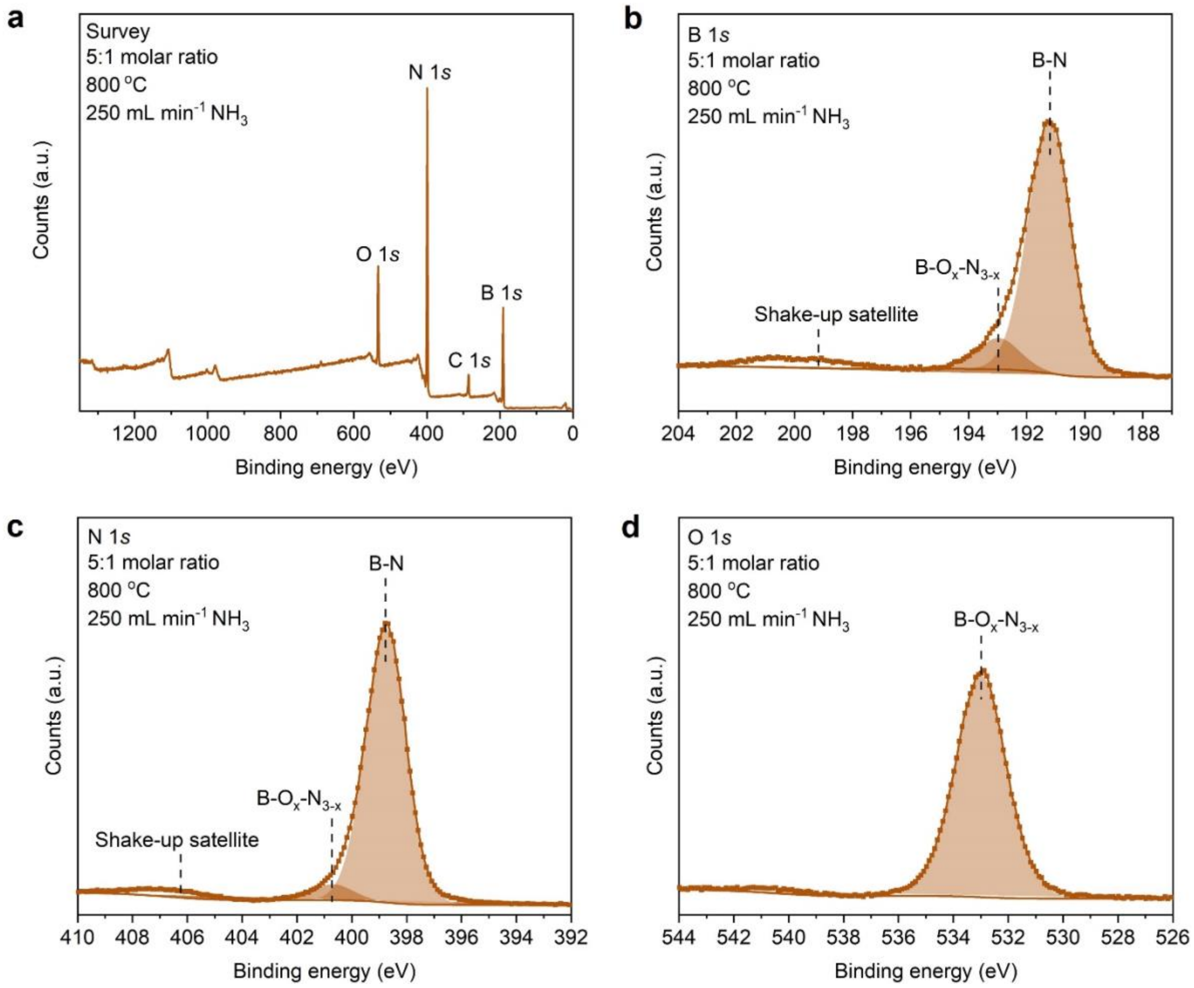

Figure S24. Chemical composition and states in BNO samples. (5:1 molar ratio, 800 ºC, 250 mL min^{-1} NH_3) - Survey spectrum and high resolution XPS core level spectra for B 1*s*, N 1*s* and O 1*s* with the key characteristic peaks and corresponding binding energies highlighted.

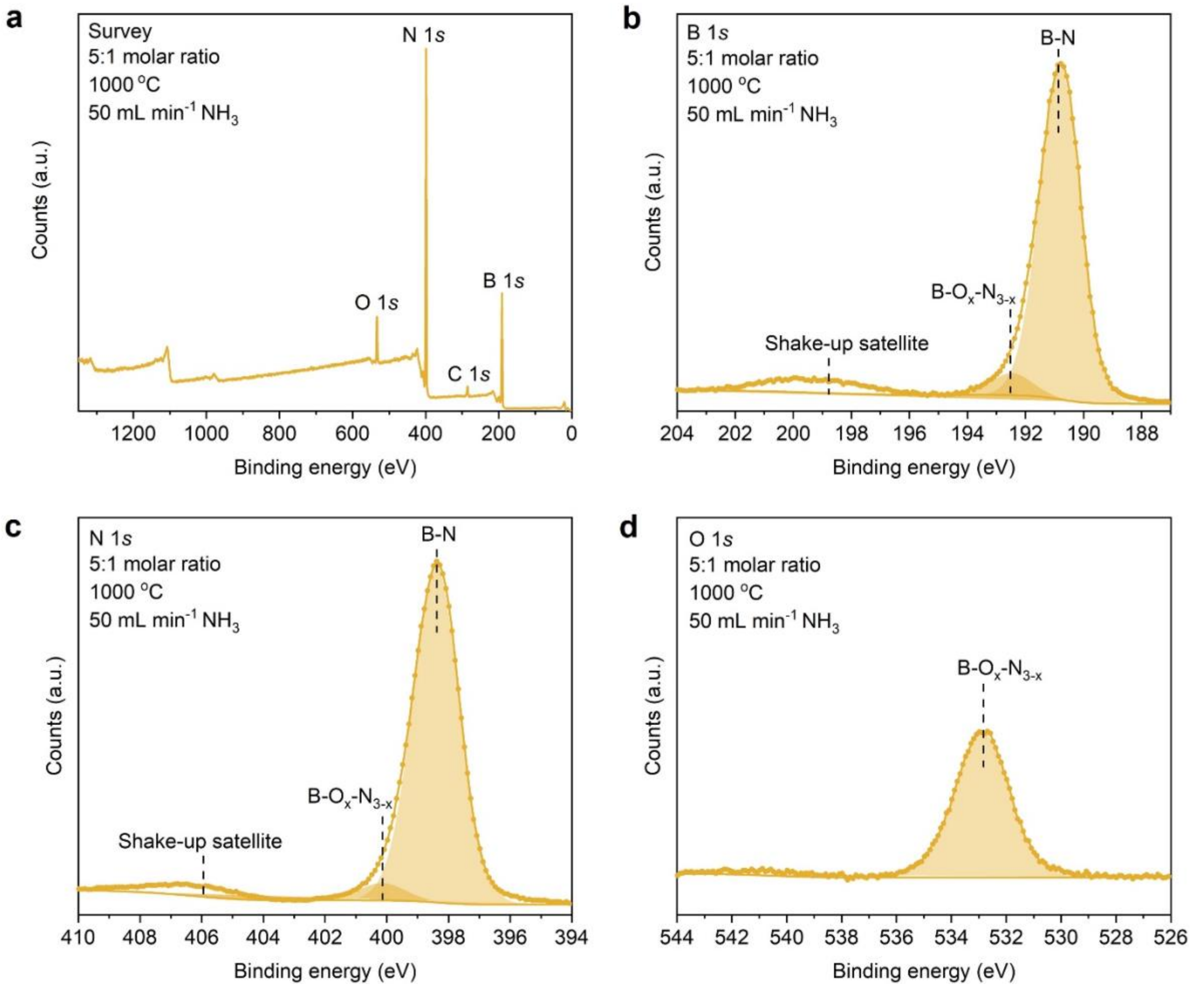

Figure S25. Chemical composition and states in BNO samples. (5:1 molar ratio, 1000 ºC, 50 mL min^{-1} NH_3) - Survey spectrum and high resolution XPS core level spectra for B 1*s*, N 1*s* and O 1*s* with the key characteristic peaks and corresponding binding energies highlighted.

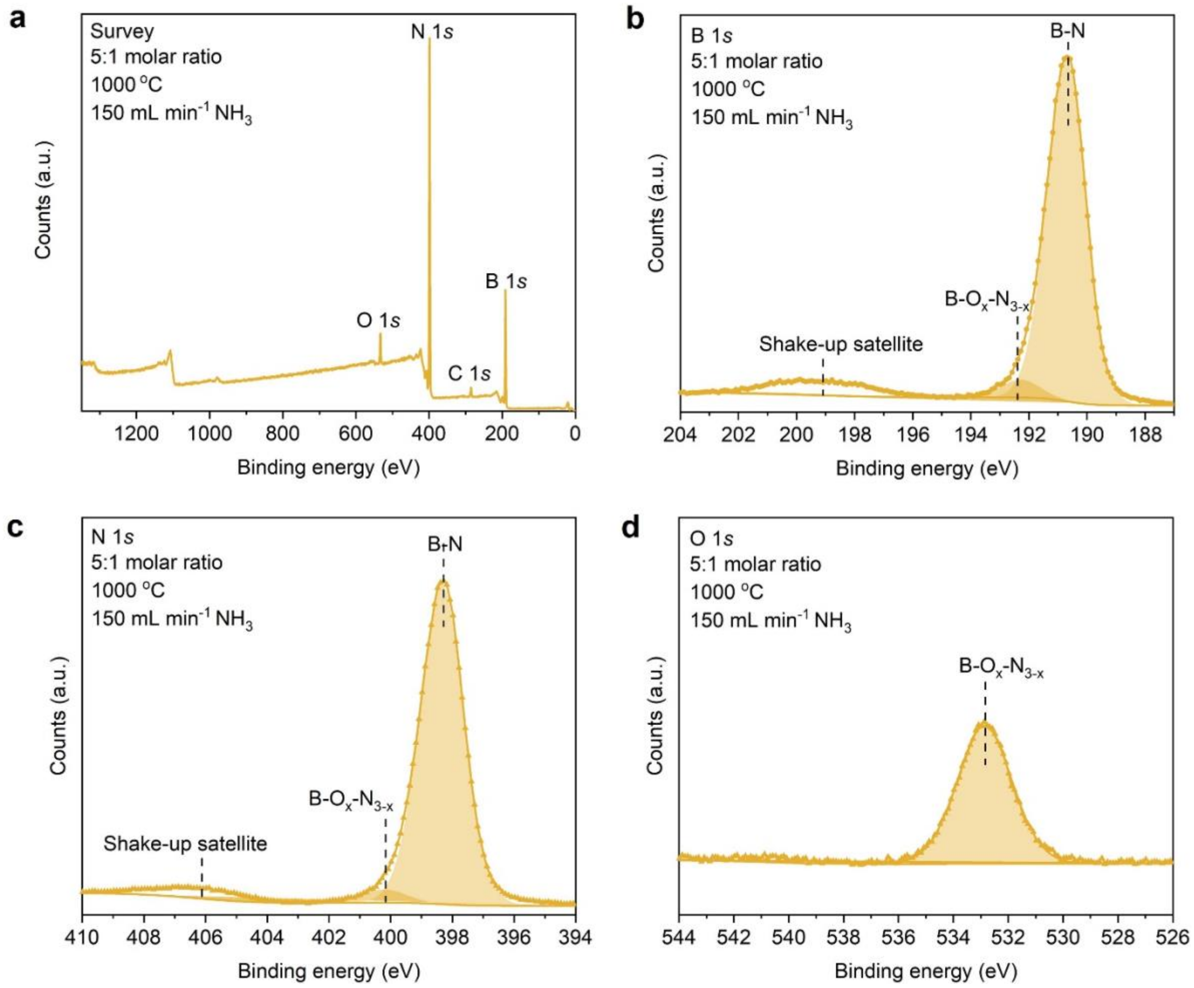

Figure S26. Chemical composition and states in BNO samples. (5:1 molar ratio, 1000 ºC, 150 mL min^{-1} NH_3) - Survey spectrum and high resolution XPS core level spectra for B 1*s*, N 1*s* and O 1*s* with the key characteristic peaks and corresponding binding energies highlighted.

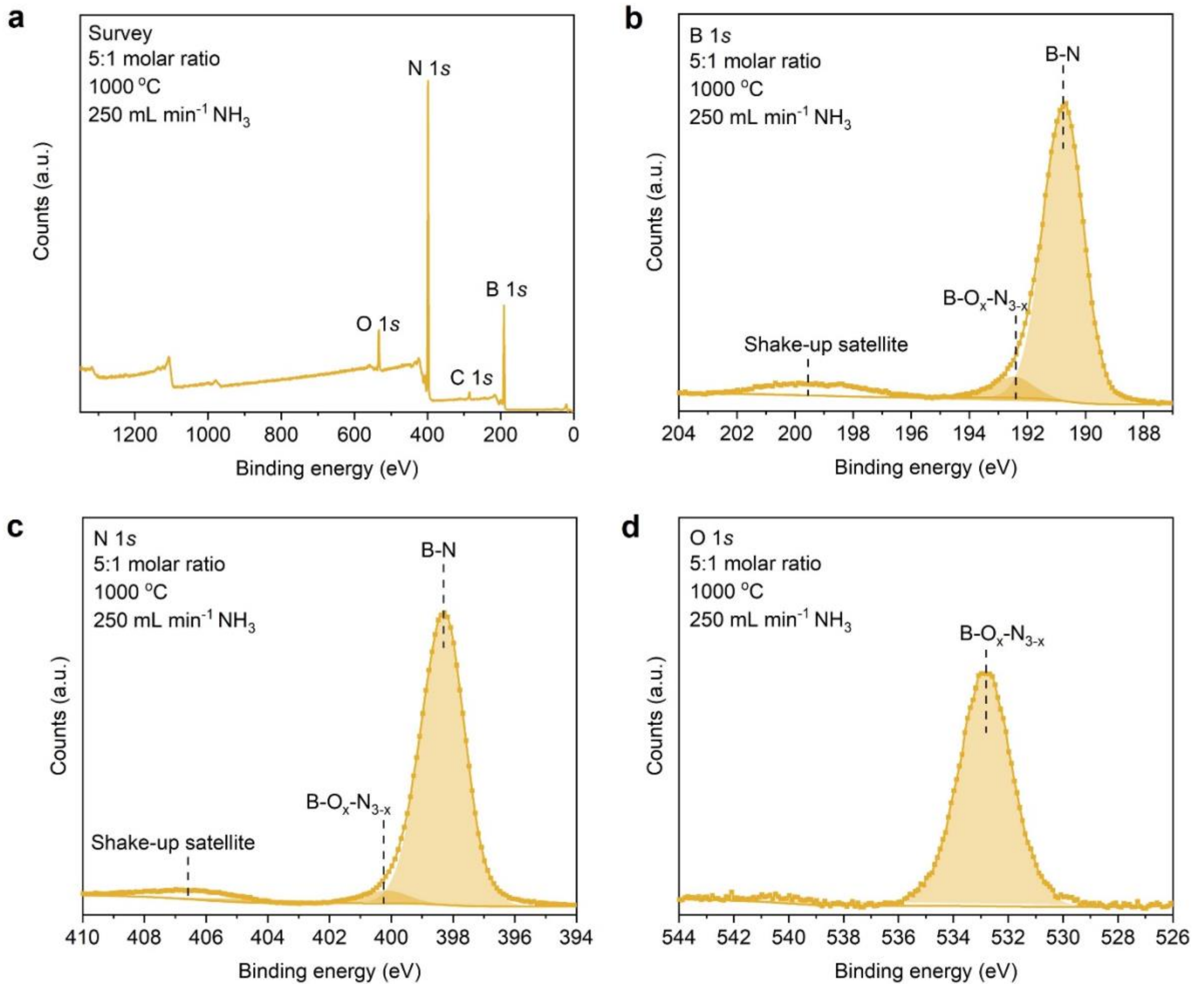

Figure S27. Chemical composition and states in BNO samples. (5:1 molar ratio, 1000 ºC, 250 mL min^{-1} NH_3) - Survey spectrum and high resolution XPS core level spectra for B 1*s*, N 1*s* and O 1*s* with the key characteristic peaks and corresponding binding energies highlighted.

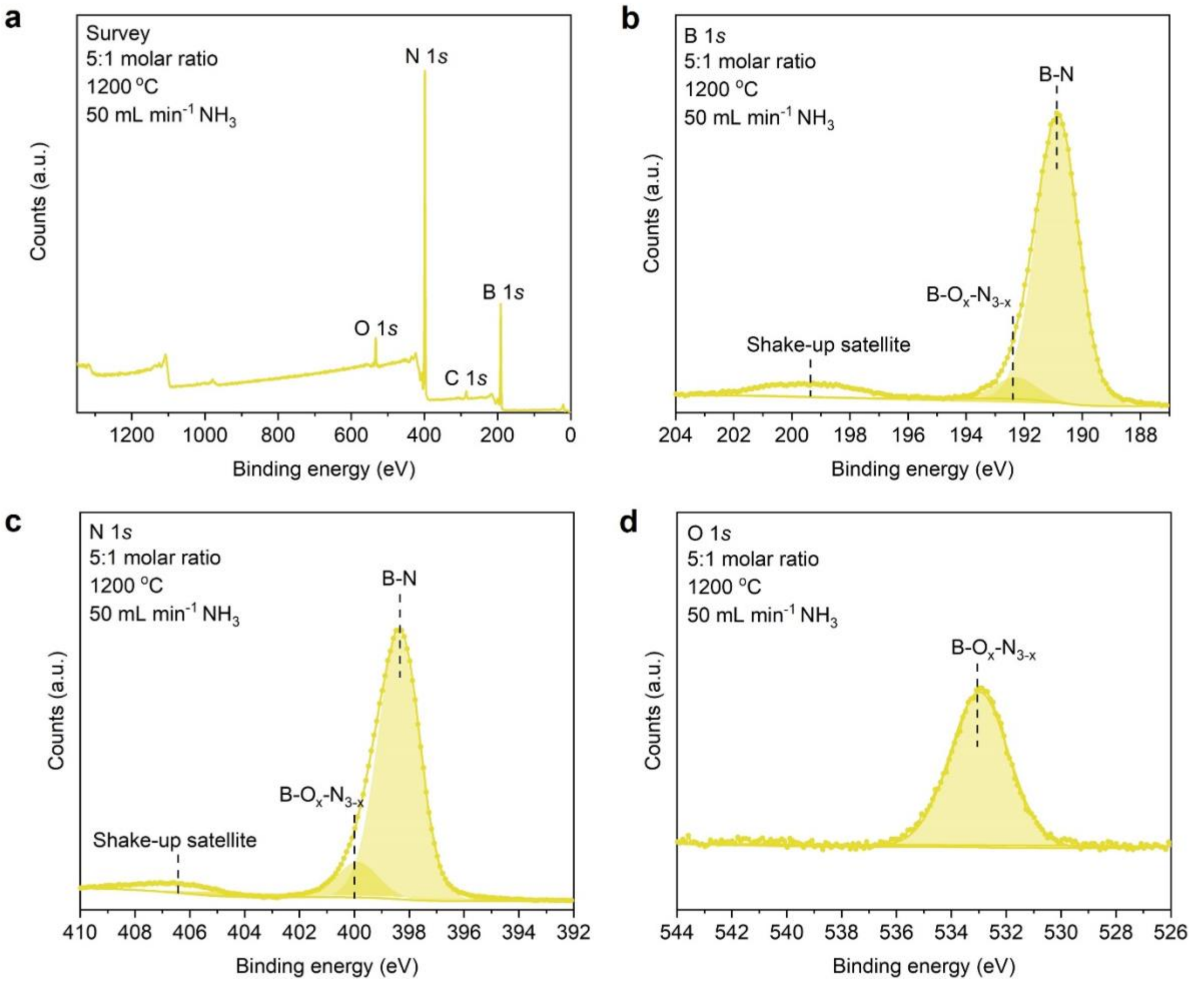

Figure S28. Chemical composition and states in BNO samples. (5:1 molar ratio, 1200 ºC, 50 mL min^{-1} NH_3) - Survey spectrum and high resolution XPS core level spectra for B 1*s*, N 1*s* and O 1*s* with the key characteristic peaks and corresponding binding energies highlighted.

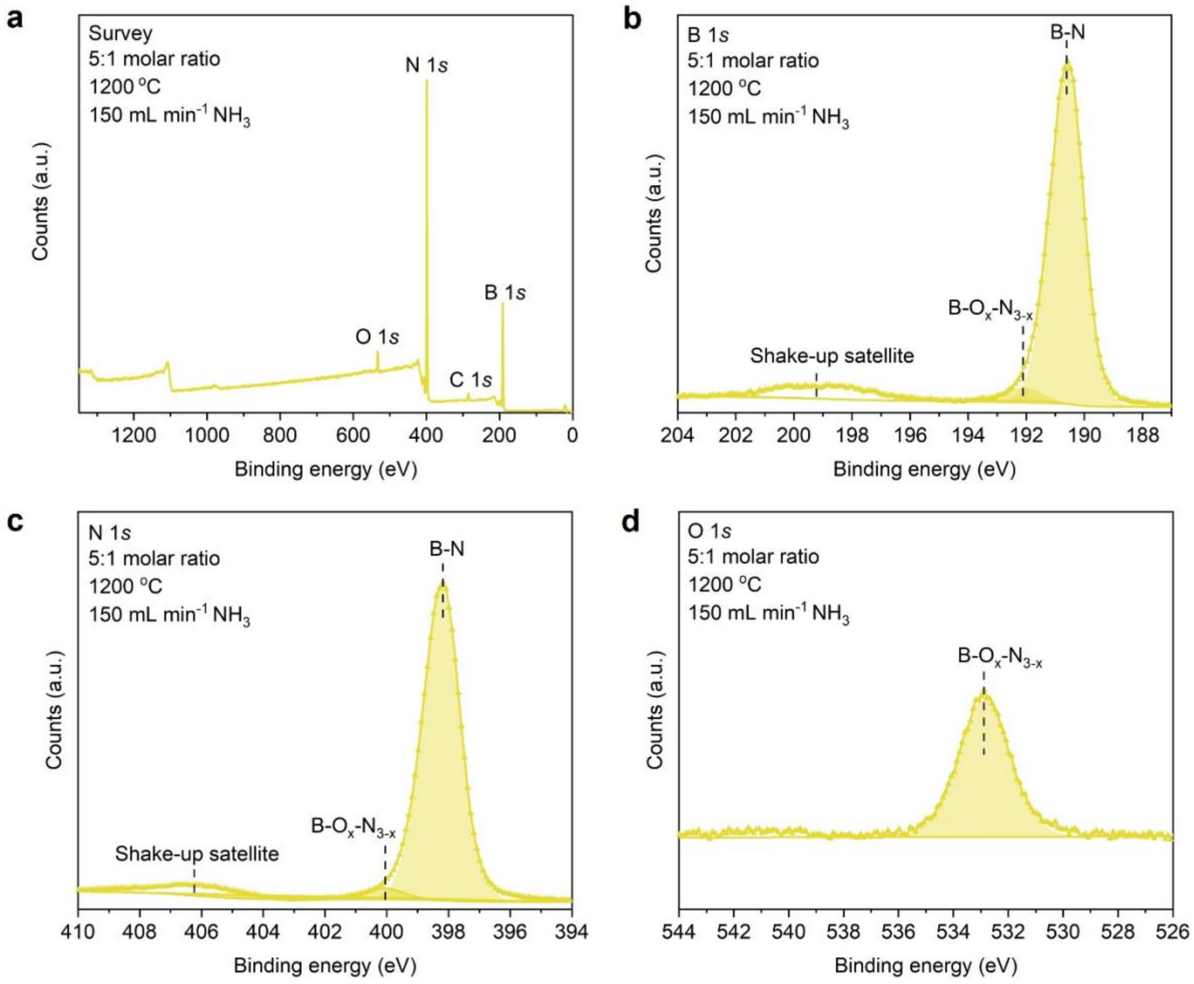

Figure S29. Chemical composition and states in BNO samples. (5:1 molar ratio, 1200 ºC, 150 mL min^{-1} NH_3) - Survey spectrum and high resolution XPS core level spectra for B 1*s*, N 1*s* and O 1*s* with the key characteristic peaks and corresponding binding energies highlighted.

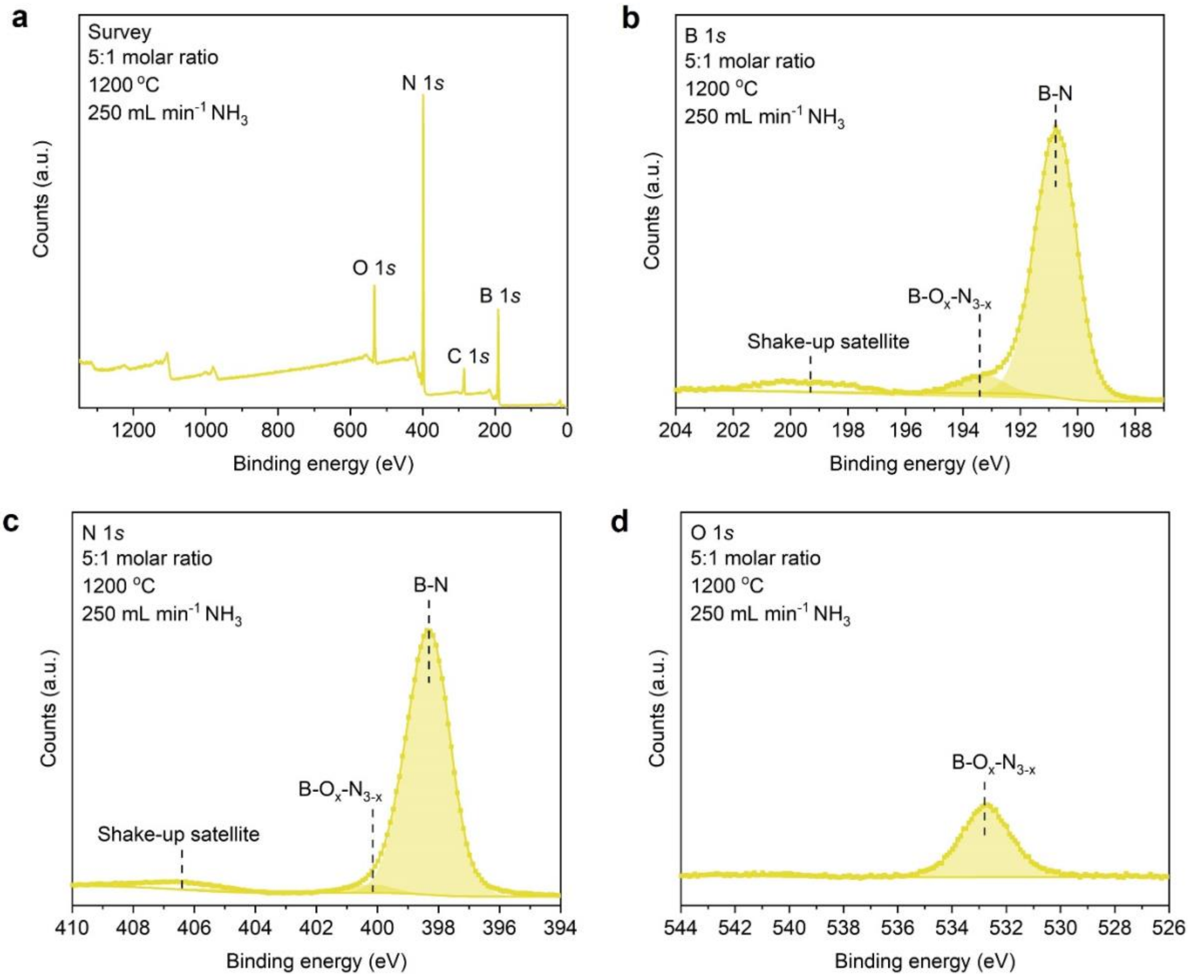

Figure S30. Chemical composition and states in BNO samples. (5:1 molar ratio, 1200 ºC, 250 mL min^{-1} NH$_3$) - Survey spectrum and high resolution XPS core level spectra for B 1*s*, N 1*s* and O 1*s* with the key characteristic peaks and corresponding binding energies highlighted.

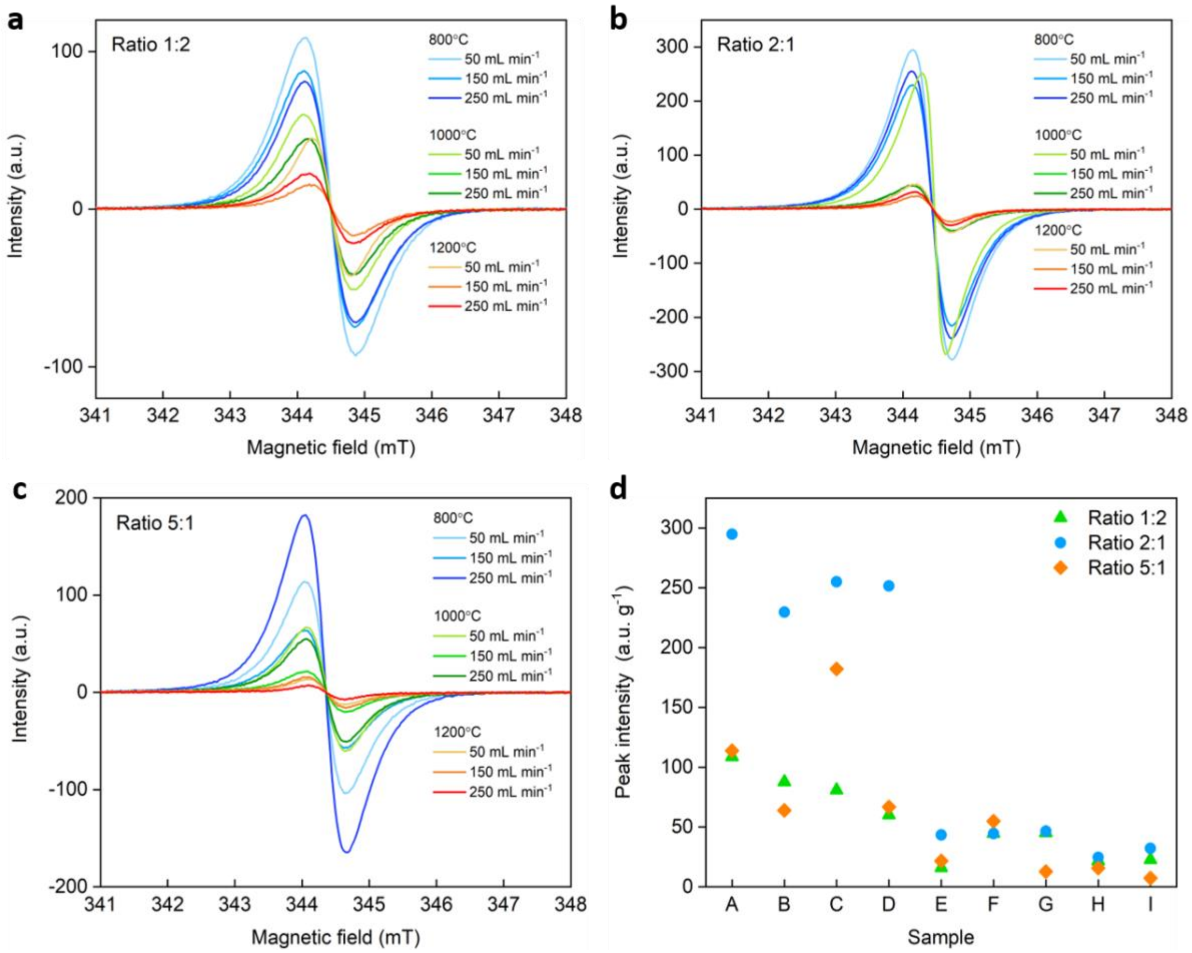

Figure S31. Room temperature X-band EPR spectra for all of the BNO samples investigated in this study.

Table S3. Summary of the oxygen contents, specific paramagnetic OB_3 intensities and band gaps of all the BNO samples investigated in this study.

Sample		Ratio (-)	Temp. (ºC)	NH_3 flowrate (mL min^{-1})	Oxygen content (at. %)	Specific OB_3 intensity (a.u. g^{-1})	Band gap (eV)
**1**	A	1:2	800	50	10.62	108.8	1.94
**2**	B	1:2	800	150	7.98	87.7	1.98
**3**	C	1:2	800	250	7.50	81.0	2.16
**4**	D	1:2	1000	50	7.01	60.1	2.69
**5**	E	1:2	1000	150	3.49	15.9	2.88
**6**	F	1:2	1000	250	2.93	44.6	2.85
**7**	G	1:2	1200	50	2.92	45.1	2.82
**8**	H	1:2	1200	150	3.44	21.7	2.89
**9**	I	1:2	1200	250	3.39	22.8	2.87
**10**	A	2:1	800	50	10.59	294.7	1.78
**11**	B	2:1	800	150	10.05	229.6	1.81
**12**	C	2:1	800	250	10.31	255.1	1.50
**13**	D	2:1	1000	50	7.38	251.6	2.26
**14**	E	2:1	1000	150	3.60	43.5	2.57
**15**	F	2:1	1000	250	3.05	44.5	2.56
**16**	G	2:1	1200	50	2.89	46.6	2.63
**17**	H	2:1	1200	150	2.13	24.7	2.77
**18**	I	2:1	1200	250	2.27	32.3	2.74
**19**	A	5:1	800	50	12.67	113.8	1.66
**20**	B	5:1	800	150	13.77	63.9	1.62
**21**	C	5:1	800	250	8.96	182.3	1.62
**22**	D	5:1	1000	50	4.27	66.8	2.41
**23**	E	5:1	1000	150	2.93	21.8	2.68
**24**	F	5:1	1000	250	4.16	55.0	2.57
**25**	G	5:1	1200	50	2.82	12.8	2.75
**26**	H	5:1	1200	150	2.15	15.7	2.82
**27**	I	5:1	1200	250	2.64	7.4	2.82

Table S4. Standard errors in the band gap measurement using UV-Vis DR spectroscopy through repeat measurements of three BNO samples.

Sample	E_G (eV) (Run 1)	E_G (eV) (Run 2)	E_G (eV) (Run 3)	Standard deviation (eV)
3	2.16	2.16	2.15	5.78×10^{-3}
4	2.69	2.69	2.68	5.78×10^{-3}
13	2.26	2.26	2.27	5.78×10^{-3}

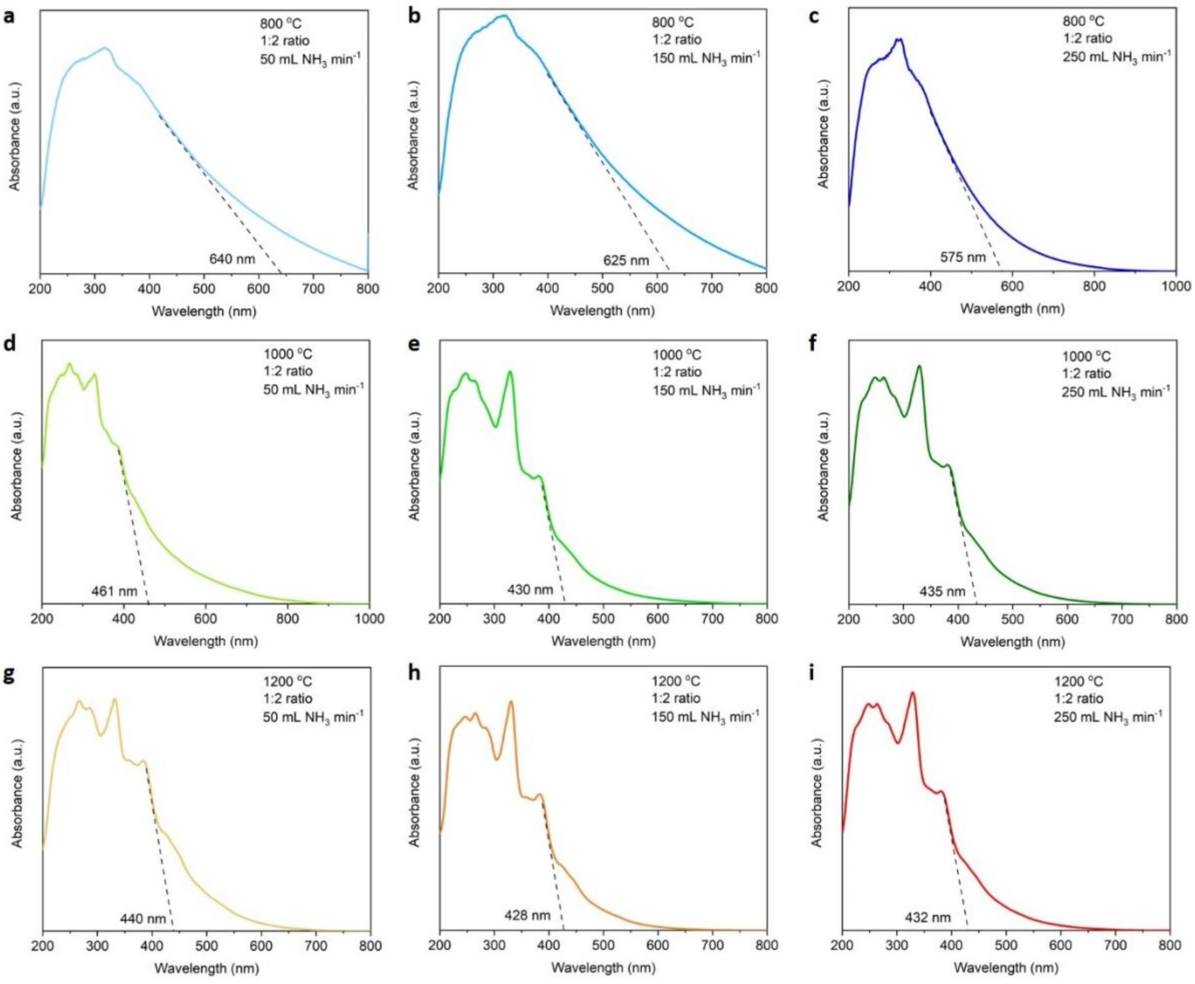

Figure S32. Absorption spectra for all of the BNO samples synthesised with a starting molar ratio of boric acid:HMTA of 1:2.

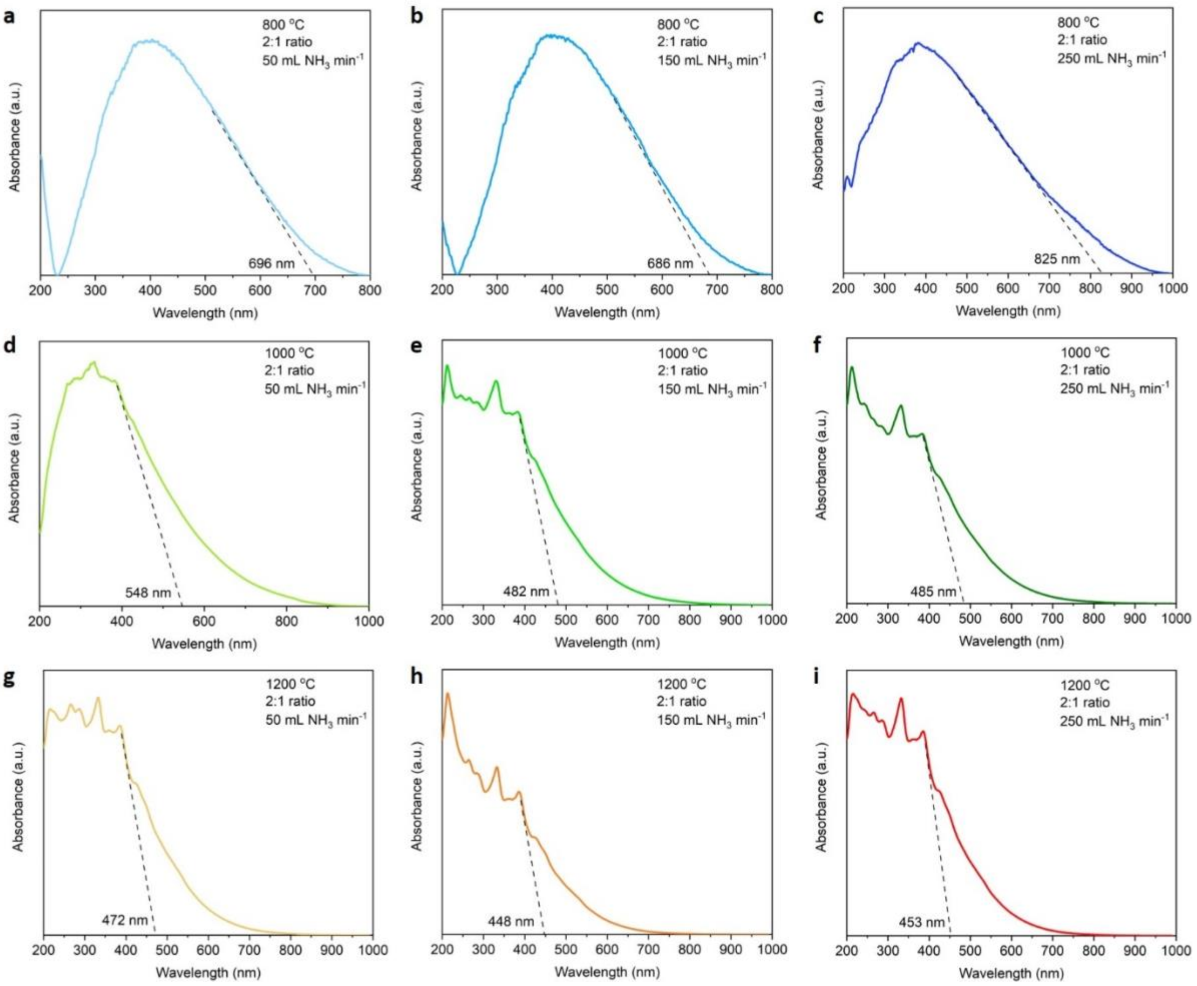

Figure S33. Absorption spectra for all of the BNO samples synthesised with a starting molar ratio of boric acid:HMTA of 2:1.

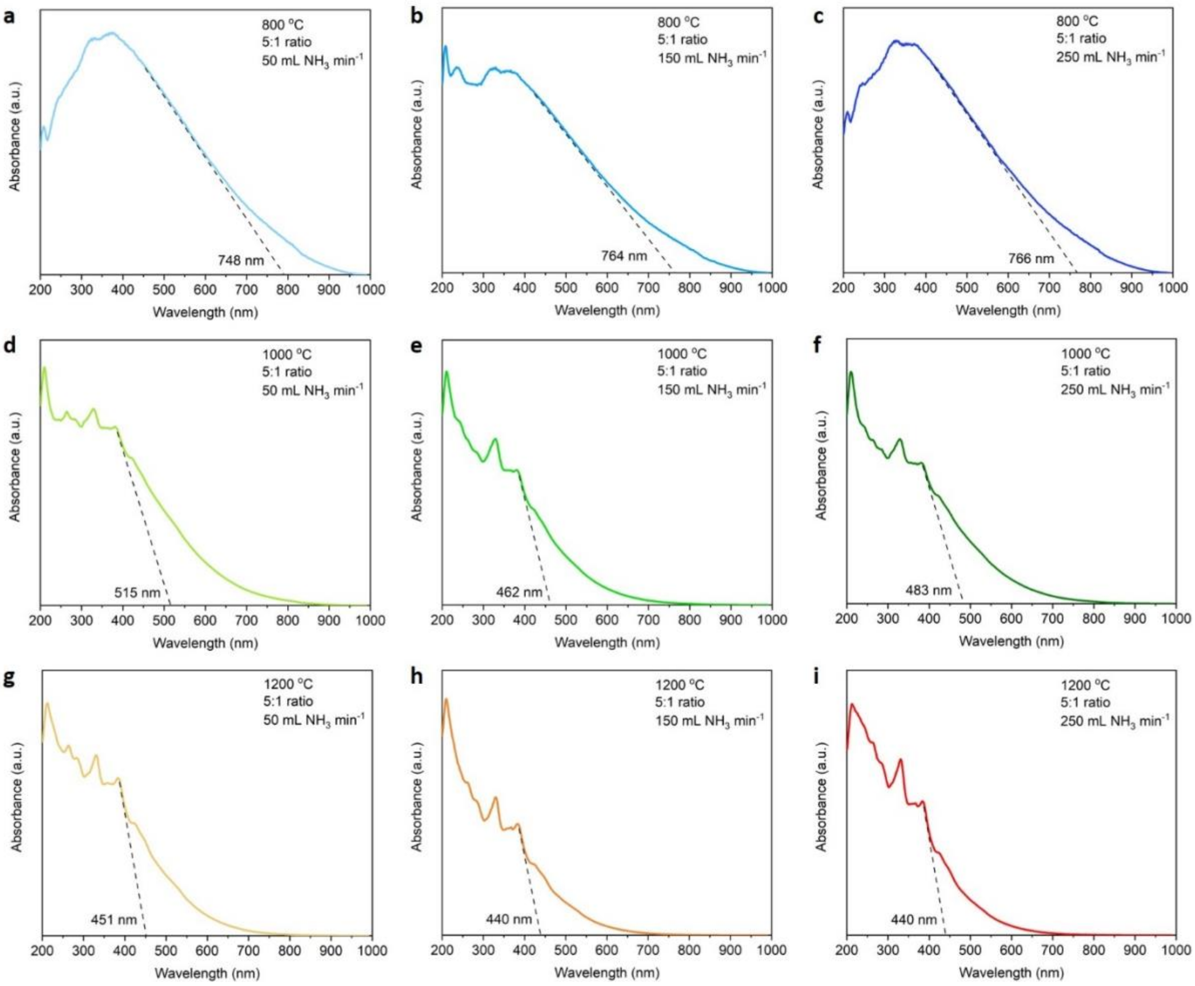

Figure S34. Absorption spectra for all of the BNO samples synthesised with a starting molar ratio of boric acid:HMTA of 5:1.

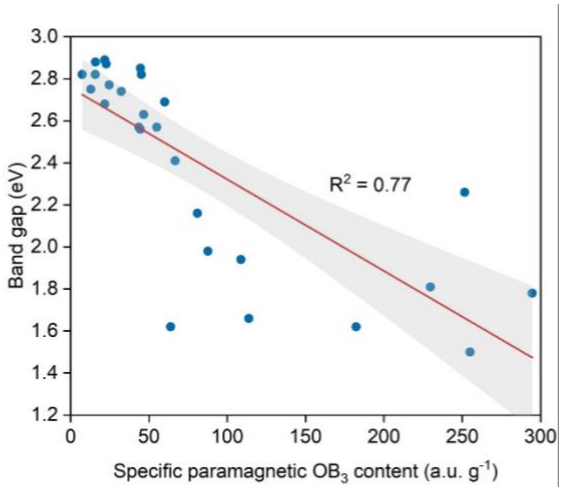

Figure S35. Scatter plot of the band gap against the paramagnetic OB_3 content (proportional to the EPR peak intensity) for all 27 BNO samples investigated in this study. Least squares linear regression was applied to obtain the line of best fit (red) and the 95% confidence intervals (grey shaded area). The coefficient of determination (R^2) illustrating the goodness of the linear fit is shown in each plot.

DOE model statistics

Stage 2 - Description of the hypothesis test

In this test, one defines a null (H_0) and an alternative (H_1) hypothesis. In essence, the null and alternative hypotheses are statements that one must either accept or reject based on the significance of the data. In this instance, we define H_0 as "the synthesis parameter does not have a statistically significant influence on the output variable of interest (oxygen content, specific paramagnetic OB_3 content or band gap)". On the other hand, we define H_1 as "the synthesis parameter has a statistically significant influence on the output variable of interest (oxygen content, specific paramagnetic OB_3 content or band gap)". To assess which statement can be accepted or rejected in the hypothesis test, the probability value (p) for each synthesis parameter in relation to the output variable is calculated in the JMP software. The p value indicates the degree to which the response follows the pattern predicted by the null hypothesis and all other assumptions used (S. Greenland, S. J. Senn, K. J. Rothman, J. B. Carlin, C. Poole, S. N. Goodman and D. G. Altman, *Eur. J. Epidemiol.*, 2016, **31**, 337-350). If $p < \alpha$ (where $\alpha = 0.05$), there is sufficiently strong evidence to reject H_0 and accept H_1 at the (1-α) confidence level. In this instance, the given synthesis parameter has a statistically significant influence on the output variable of interest. If $p > \alpha$, then there is insufficient evidence to reject H_0 and accept H_1 at the (1-α) significance level. In this case, the given synthesis parameter does not have a statistically significant influence on the output variable of interest. However, in the event that higher order (quadratic or combinatoric) parameters are statistically significant, then the individual term(s) containing higher order parameters must be included in the response surface design, even if the p value for this individual term exceeds 0.05. Omission of the individual term(s) from a higher order parameter can result in inaccurate response surface design, and hence model predictions (J. Sall, M. L. Stephens, A. Lehman and S. Loring, *JMP start statistics: a guide to statistics and data analysis using JMP*, Sas Institute, 2017). For example, the term R^2 is considered a statistically significant parameter for the specific paramagnetic OB_3 content ($p = 1.60 \times 10^{-3}$) (Table S6). As such, the term R must

be included in the response surface design even though its p value is 0.82. Similarly, as $T{\cdot}R$ is statistically significant for the band gap ($p < 0.05$) (Table S5), the term R must be included despite $p > 0.05$ for this term.

Stage 3 – Description of the studentised residuals test

The studentised residuals test is a multivariate statistical technique that evaluates whether individual data points across a large, multi-dimensional population introduces bias to the system, which could skew the regression analysis or model predictions. The software calculates the studentised residual for each point by dividing the absolute residual (difference between experimental and model values) by an estimate of its standard deviation. Upper and lower control limits (UCL and LCL) are then calculated, which are defined as the residual mean plus or minus three standard deviations (3σ). Data points lying above or below the UCL and LCL, respectively, are deemed as outliers, which could impose bias on the data set and skew the response surface design and/or model predictions.

Stage 4 – Description of the Cook's distance test

To identify influential data points, we apply the Cook's distances (Cook's D) test. This test identifies whether individual data points with large residuals do not alter the accuracy of the model predictions. If a single point has a large Cook's D, this would suggest that removing this point would significantly skew the line of best fit between the model predictions and experimental values, which would negatively affect the prediction accuracy. Ideally, one would expect a narrow distribution of Cook's D values below 1, resulting in a short box-and-whisker plot and densely populated histogram at low (< 1) values. Typically, an influential data point is identified by a Cook's D value greater than 1 (S. Chatterjee and A. S. Hadi, *Regression analysis by example*, John Wiley & Sons, 2015).

Stage 5 – Description of the Q-Q plots and Shapiro-Wilk test

The normal Q-Q graph is a probability plot of the actual residuals for the oxygen content, specific paramagnetic OB_3 content and band gap against the expected values if the residuals were to follow a perfect normal distribution. If the points lie close to the line of best fit and exhibit a linear trend, this shows that the residuals follow a normal distribution. We further quantitatively confirmed this by conducting the Shapiro-Wilk test. This is a statistical test that assesses the statistical distribution of a given data set (S. S. Shapiro and M. B. Wilk, *Biometrika*, 1965, **52**, 591-611; E. H. Chen, *J. Am. Stat. Assoc*, 1971, **66**, 760-762). We follow a similar hypothesis testing process at the 5% significance level (α = 0.05), as previously outlined for identifying the statistically significant synthesis parameters. For the Shapiro-Wilk test, we define the null hypothesis (H_0) as "the residuals for the oxygen content, specific paramagnetic OB_3 content and band gap follow a normal distribution". The alternative hypothesis (H_1) is "the residuals for the oxygen content, specific paramagnetic OB_3 content and band gap follow anything but a normal distribution". Again, if $p < \alpha$ (where $\alpha = 0.05$), there is sufficiently strong evidence to reject H_0 and accept H_1 at the (1-α) confidence level. In our case, the p values for the oxygen content, specific paramagnetic OB_3 content and band gap residuals were determined as 0.16, 0.21 and 0.06, respectively (Figures S41). As such, there is insufficient evidence to reject H_0 and accept H_1 as $p > 0.05$ in all cases. Hence, the Shapiro-Wilk test shows that the residuals for each output variable follows a normal distribution at the 95% confidence level.

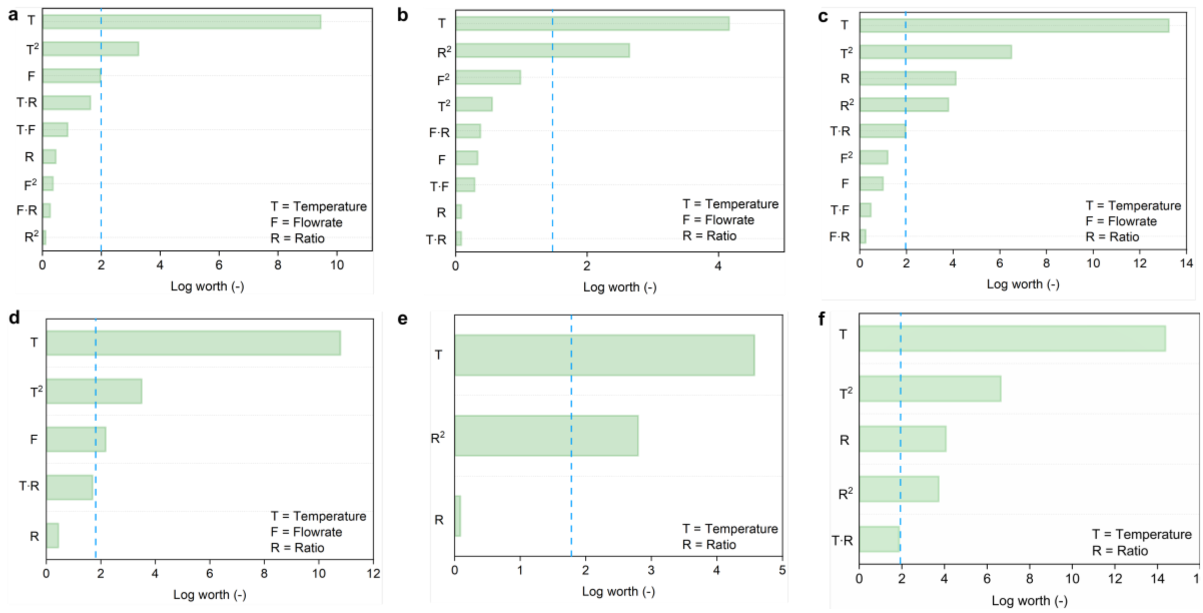

Figure S36. Effects summaries for: (**a, d**) oxygen content, (**b, e**) paramagnetic OB_3 content and (**c, f**) band gap (**a** – **c**) before and (**d** – **f**) after removing the statistically insignificant parameters.

Table S5. Summary of the *p*-values of the individual, quadratic or combinatoric synthesis parameters for the oxygen content before and after removing statistically insignificant parameters. In the "before" scenario, we consider all possible individual, quadratic or combinatoric synthesis parameters. In the "after" scenario, we have removed the parameters that are statistically insignificant (denoted by '-'). The parameters highlighted in bold are considered statistically significant at the 5 % significance level since $p < 0.05$. In the event that higher order (quadratic or combinatoric) parameters are statistically significant, the individual term(s) of these higher order parameters must be included in the model even if the *p* value for this individual term exceeds 0.05.

**Parameter**	***p* value (-) Before**	**Parameter**	***p* value (-) After**
**T**	**1.0×10^{-6}**	T	1.0×10^{-6}
**T^2**	**5.5×10^{-4}**	T^2	3.2×10^{-4}
**F**	**1.1×10^{-2}**	F	6.7×10^{-3}
**T·R**	**2.4×10^{-2}**	T·R	2.0×10^{-2}
T·F	0.14	R	0.37
**R**	**0.36**	F·R	-
F^2	0.44	F^2	-
F·R	0.55	R^2	-
R^2	0.79	T·F	-

Table S6. Summary of the *p*-values of the individual, quadratic or combinatoric synthesis parameters for the specific paramagnetic OB_3 content before and after removing statistically insignificant parameters. In the "before" scenario, we consider all possible individual, quadratic or combinatoric synthesis parameters. In the "after" scenario, we have removed the parameters that are statistically insignificant (denoted by '-'). The parameters highlighted in bold are considered statistically significant at the 5 % significance level since $p < 0.05$. In the event that higher order (quadratic or combinatoric) parameters are statistically significant, the individual term(s) of these higher order parameters must be included in the model even if the *p* value for this individual term exceeds 0.05.

Parameter	*p* value (-) Before	Parameter	*p* value (-) After
**T**	**7.0×10^{-5}**	T	3.0×10^{-5}
**R^2**	**2.3×10^{-3}**	R^2	1.6×10^{-3}
F^2	0.10	R	0.82
T^2	0.28	F^2	-
F·R	0.42	T^2	-
F	0.46	F·R	-
T·F	0.52	F	-
**R**	**0.82**	T·F	-
T·R	0.82	T·R	-

Table S7. Summary of the *p*-values of the individual, quadratic or combinatoric synthesis parameters for the band gap before and after removing statistically insignificant parameters. In the "before" scenario, we consider all possible individual, quadratic or combinatoric synthesis parameters. In the "after" scenario, we have removed the parameters that are statistically insignificant (denoted by '-'). The parameters highlighted in bold are considered statistically significant at the 5 % significance level since $p < 0.05$. In the event that higher order (quadratic or combinatoric) parameters are statistically significant, the individual term(s) of these higher order parameters must be included in the model even if the *p* value for this individual term exceeds 0.05.

**Parameter**	***p* value (-) Before**	**Parameter**	***p* value (-) After**
**T**	**1.0×10^{-6}**	T	1.0×10^{-6}
**T^2**	**1.0×10^{-6}**	T^2	1.0×10^{-6}
**R**	**8.0×10^{-5}**	R	1.3×10^{-4}
**R^2**	**1.6×10^{-4}**	R^2	2.7×10^{-4}
**T·R**	**1.1×10^{-2}**	T·R	1.7×10^{-2}
F^2	6.5×10^{-2}	F	-
F	0.10	F^2	-
T·F	0.34	F·R	-
F·R	0.57	T·F	-

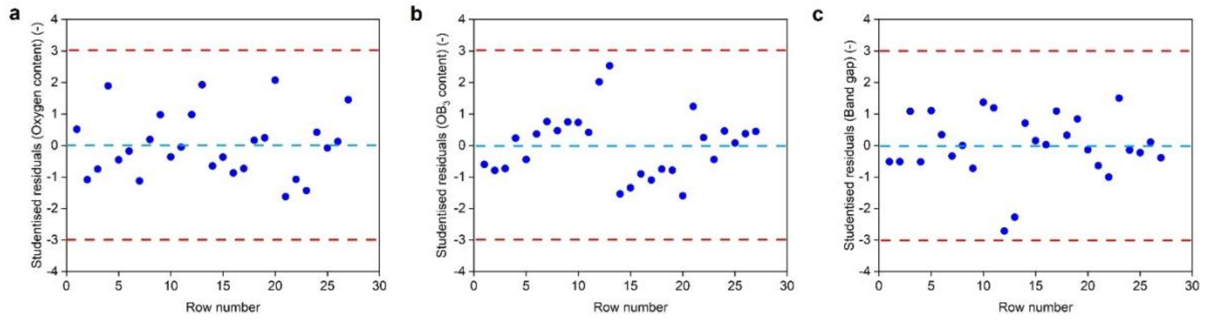

Figure S37. Studentised residuals plots for the (**a**) oxygen content, (**b**) specific paramagnetic OB_3 content, (**c**) band gap. The blue dashed line represents the ideal scenario with zero studentised residual. The red dashed lines represent the upper and lower control limits of ± 3σ, where σ denotes the standard deviation.

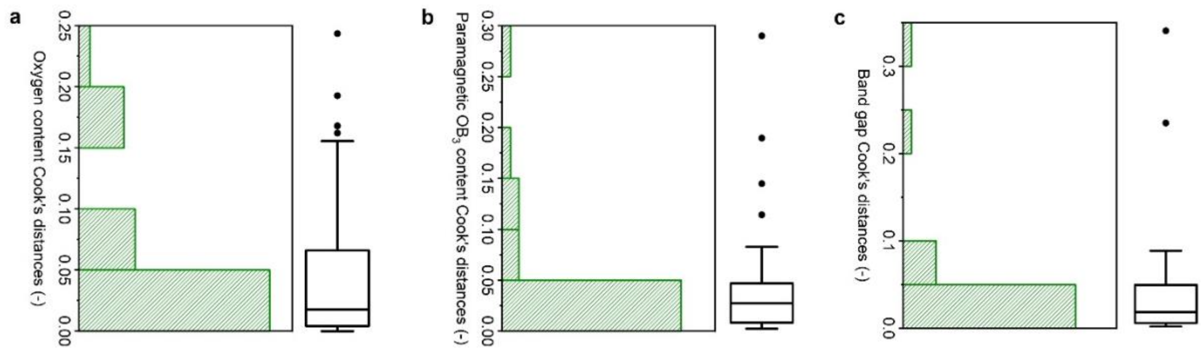

Figure S38. Histogram and box and whisker plot for Cook's distances test to identify influential data points amongst the (**a**) oxygen content, (**b**) specific paramagnetic OB_3 content and (**c**) band gap data.

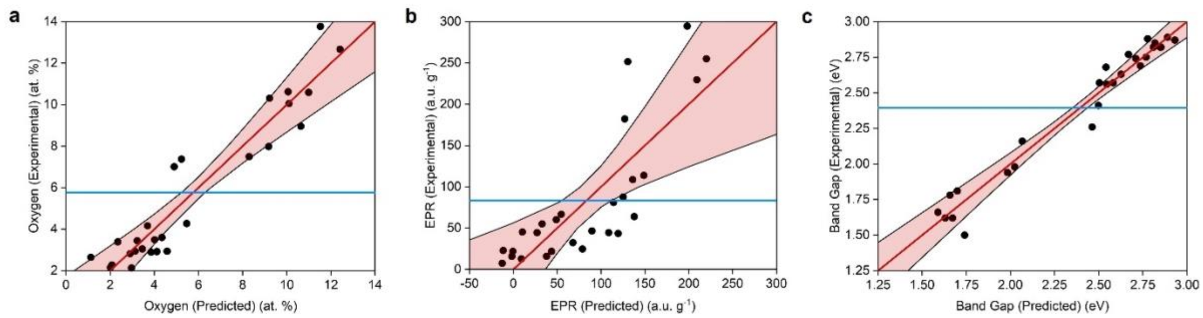

Figure S39. Leverage plots comparing the experimental values and model predictions for the (**a**) oxygen content, (**b**) paramagnetic OB_3 content and (**c**) band gap. The 95% confidence bands (grey curves) provide a visual indication of whether the test of interest (the predicted mean) is significant at the 5% level. For a model to be insignificant, the confidence bands would encompass the horizontal mean line (blue line) wholly and therefore not cross the mean line. This would mean that the model cannot distinguish between fluctuations in the predicted response owing to natural variation and fluctuations due to systematically changing the synthesis parameters. As all the confidence curves cross the mean line for oxygen content, specific paramagnetic OB_3 content and band gap, the response can be qualitatively considered significant.

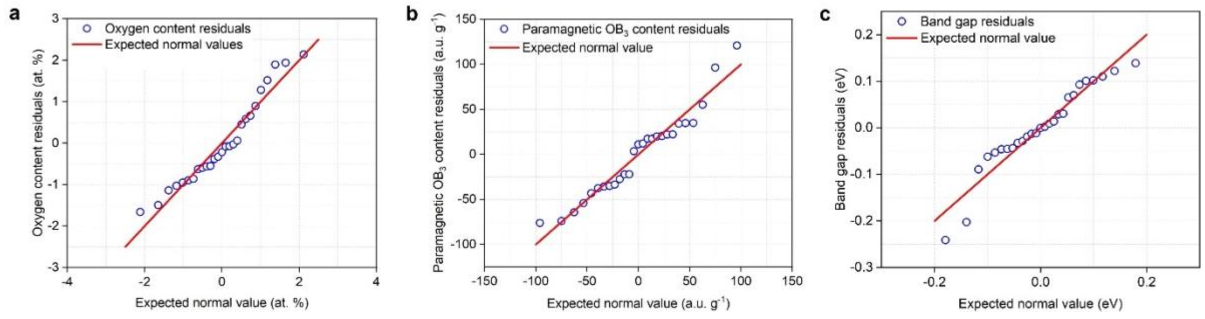

Figure S40. Normal quantile-quantile plots assessing the goodness of fit between the experimental values and model predictions for (**a**) oxygen content, (**b**) specific paramagnetic OB_3 content and (**c**) band gap, by examining whether the residuals follow a normal distribution.

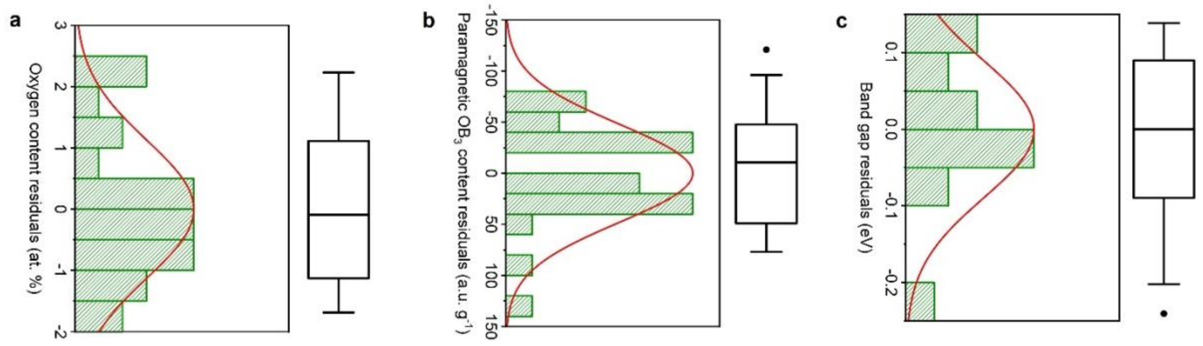

	Oxygen content Shapiro-Wilk normality	***Paramagnetic OB_3 content Shapiro-Wilk normality***	***Band Gap Shapiro-Wilk normality***
***p* values (-)**	0.16	0.21	0.06

Figure S41. Histogram and box and whisker plot for the (**a**) oxygen content, (**b**) paramagnetic OB_3 content and (**c**) band gap residuals used for the Shapiro-Wilk normality tests to assess the goodness of fit of the experimental values and model predicted values.

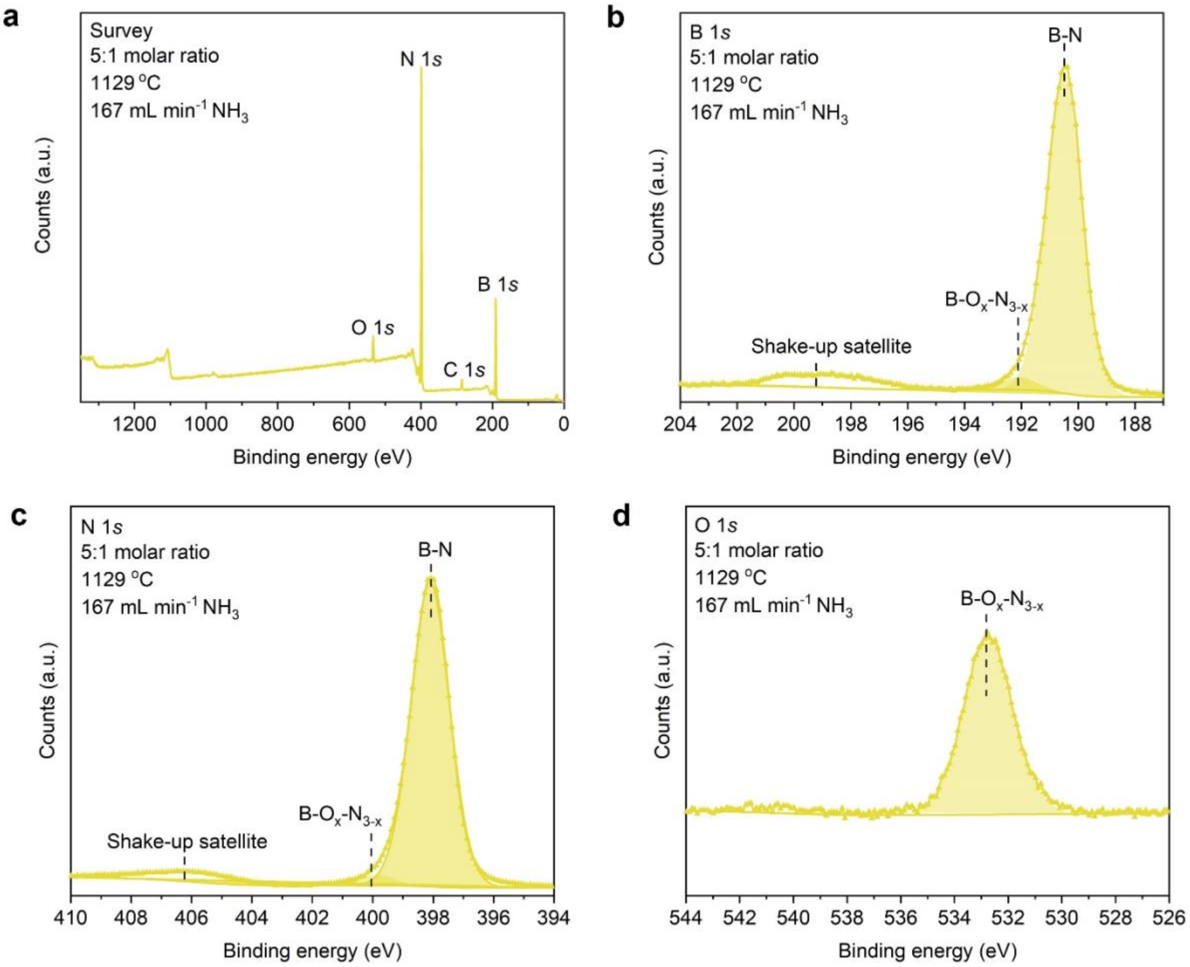

Figure S42. Chemical composition and states in low oxygen content BNO sample for model predictions. (5:1 molar ratio, 1129 ºC, 167 mL min^{-1} NH_3) - Survey spectrum and high resolution XPS core level spectra for B 1*s*, N 1*s* and O 1*s* with the key characteristic peaks and corresponding binding energies highlighted.

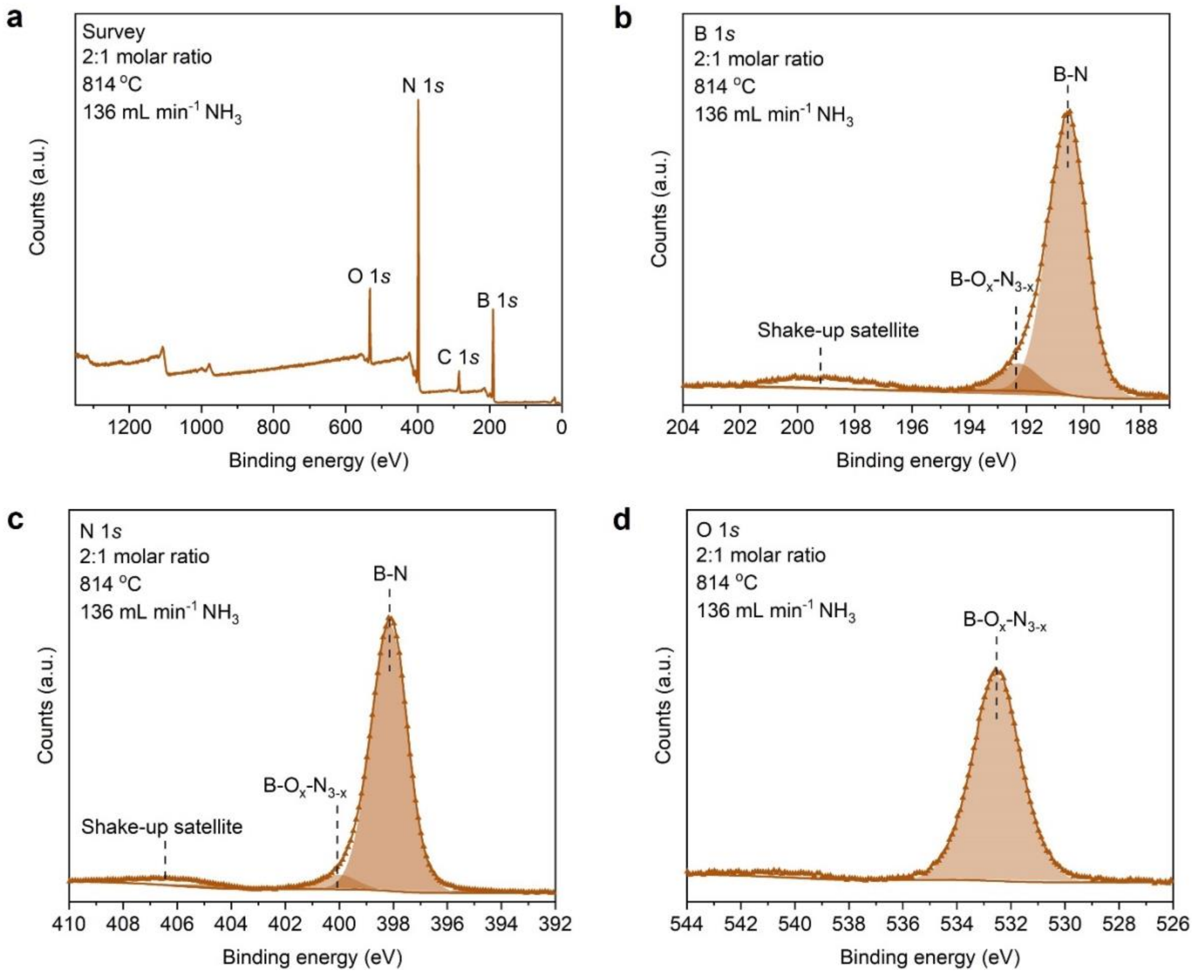

Figure S43. Chemical composition and states in low oxygen content BNO sample for model predictions. (2:1 molar ratio, 814 ºC, 136 mL min^{-1} NH_3) - Survey spectrum and high resolution XPS core level spectra for B 1*s*, N 1*s* and O 1*s* with the key characteristic peaks and corresponding binding energies highlighted.

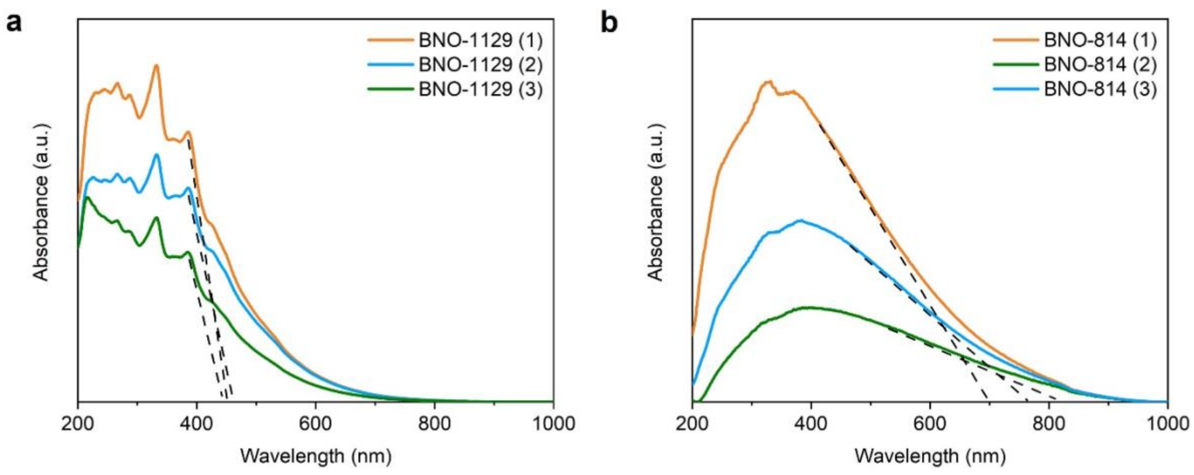

Figure S44. Absorption spectra used to determine the band gaps and verify the model predictions for the (**a**) high (BNO-1129: 1129 ^{o}C, 5:1 molar ratio, 167 mL min^{-1}) and (**b**) low (BNO-814: 814 ^{o}C, 2:1 molar ratio, 136 mL min^{-1}) band gap BNO samples. An average of the three band gaps, determined from the absorption edge, was calculated with a standard error.
